# Multifunctional nanoparticle-mediated combining therapy for human diseases

**DOI:** 10.1038/s41392-023-01668-1

**Published:** 2024-01-01

**Authors:** Xiaotong Li, Xiuju Peng, Makhloufi Zoulikha, George Frimpong Boafo, Kosheli Thapa Magar, Yanmin Ju, Wei He

**Affiliations:** 1 School of Pharmacy, China Pharmaceutical University, Nanjing, 2111198 PR China; 2 Xiangya School of Pharmaceutical Sciences, Central South University, Changsha, 410013 PR China; 3 Shanghai Skin Disease Hospital, Tongji University School of Medicine, Shanghai, 200443 China

**Keywords:** Cancer, Drug discovery, Immunology, Cardiovascular diseases, Medical research

## Abstract

Combining existing drug therapy is essential in developing new therapeutic agents in disease prevention and treatment. In preclinical investigations, combined effect of certain known drugs has been well established in treating extensive human diseases. Attributed to synergistic effects by targeting various disease pathways and advantages, such as reduced administration dose, decreased toxicity, and alleviated drug resistance, combinatorial treatment is now being pursued by delivering therapeutic agents to combat major clinical illnesses, such as cancer, atherosclerosis, pulmonary hypertension, myocarditis, rheumatoid arthritis, inflammatory bowel disease, metabolic disorders and neurodegenerative diseases. Combinatorial therapy involves combining or co-delivering two or more drugs for treating a specific disease. Nanoparticle (NP)-mediated drug delivery systems, i.e., liposomal NPs, polymeric NPs and nanocrystals, are of great interest in combinatorial therapy for a wide range of disorders due to targeted drug delivery, extended drug release, and higher drug stability to avoid rapid clearance at infected areas. This review summarizes various targets of diseases, preclinical or clinically approved drug combinations and the development of multifunctional NPs for combining therapy and emphasizes combinatorial therapeutic strategies based on drug delivery for treating severe clinical diseases. Ultimately, we discuss the challenging of developing NP-codelivery and translation and provide potential approaches to address the limitations. This review offers a comprehensive overview for recent cutting-edge and challenging in developing NP-mediated combination therapy for human diseases.

## Introduction

**Fig. 1:**
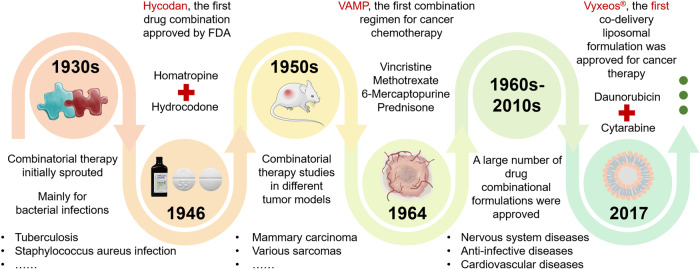
Timeline mapping the historical development and advancement of combinatorial therapies. Parts of the figure were drawn using Servier Medical Art licensed under a Creative Commons Attribution 3.0 Unported License (https://creativecommons.org/licenses/by/3.0/)

Combined therapy, a management model that involves two or more active compounds, is playing an increasing role in combating human diseases.^1^ Clinical mainstream diseases, including cancer, cardiovascular disorder, inflammatory bowel disease (IBD), lung diseases, rheumatoid arthritis (RA), and metabolic disorders, have complex microenvironments and interconnected pathological pathways, so many conventional monotherapies always have moderate efficacy. Given the advantages, such as targeting multiple signaling pathways, elevated treatment efficacy, reduced administration dose and side effects, and decreased drug resistance,^2,3^ combinatorial treatments are promising strategies to combat major diseases (Fig. 1). Moreover, the combined therapy represents a new approach for “drug repurposing” regarding using approved drugs for new therapeutic purposes, allowing reduced business risk and development costs.^4^ Nonetheless, the cocktail-drug combinations could also potentially cause the treatment outcomes, e.g., antagonism and increased drug toxicity, due to the restrictions, including drugs’ pharmacokinetic difference, asynchronous tissue biodistribution, poor barrier penetration, and intracellular delivery.^5^ For instance, the combined use of small molecular drugs and active proteins demonstrates effective efficacy to regular cells’ performance in vitro. However, dosing their cocktail combination frequently shows suboptimal therapeutic efficacy because of the protein degradation by the livers and poor internalization by cells.

**Fig. 2:**
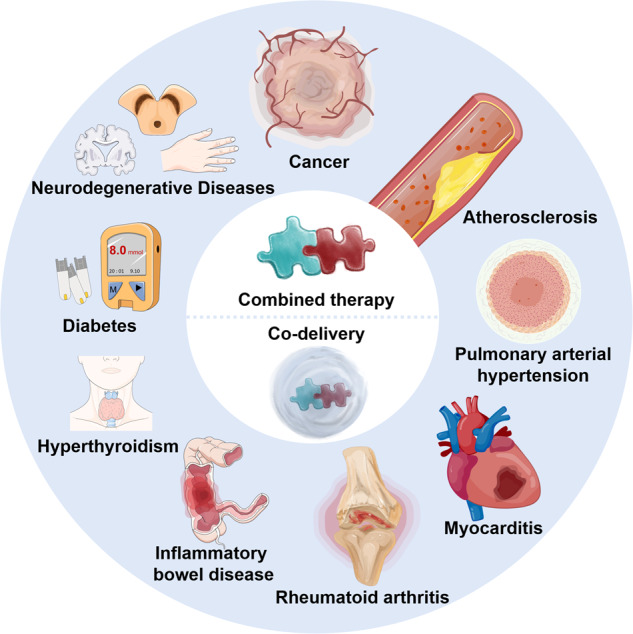
Combinatorial therapy and NP-codelivery therapy strategies for human diseases. Parts of the figure were drawn using Servier Medical Art licensed under a Creative Commons Attribution 3.0 Unported License (https://creativecommons.org/licenses/by/3.0/)

Multifunctional NP-based drug delivery systems (DDSs) are emerging as a robust approach to improve the combined therapy as they can load the active agents into one carrier, improve drug solubility, protect the drug from decomposition, alter the biodistribution, elevate tissue penetration, avoid rapid clearance, prolong half-life, and reduce off-target effects. More importantly, these DDSs enable the simultaneous or spatial delivery of two or more drugs, allowing the consistent pharmacokinetic performance of different drugs and maximizing synergistic effects.^6–11^ E.g., responsive-release DDSs, such as enzyme- and pH-triggered NPs, can release their payloads in sequence and allow precise delivery to different lesion sites or organelles.^12–15^ Additionally, the asynchronous release of the two drugs from DDSs after endocytosis could magnify the synergy since they have a spatiotemporal inconsistency in the intracellular target. E.g., biological drugs constantly need increased time to demonstrate their activity post uptake compared with active compounds. A co-delivery system assembled from drug crystals and microRNAs enabled sustained release of the drug over time and, whereas, rapid release of the biologics, improving the synergy to kill cancer cells or alleviate inflammation.^16,17^ Also, these NP preparations can be given *via* several routes, including oral, injection, transdermal, and inhalation, thereby increasing the potentiality of clinical use.^18^ Up to now, a liposomal formulation (Vyxeos^®^) co-loading with daunorubicin (DNR) and cytarabine (ara-C) was approved in 2017 for treating acute myeloid leukemia (t-AML) and myelodysplasia-related AML,^19^ demonstrating the breakthrough of multifunctional NP-mediated combining therapy. This review introduces the complex pathological mechanisms for some clinically critical diseases and therapeutic targets and discusses combinatorial therapy strategies used in the clinic. Primarily, we highlight NP-codelivery therapy and its directions and challenges (Fig. 2).

## Multifunctional NPs

**Fig. 3:**
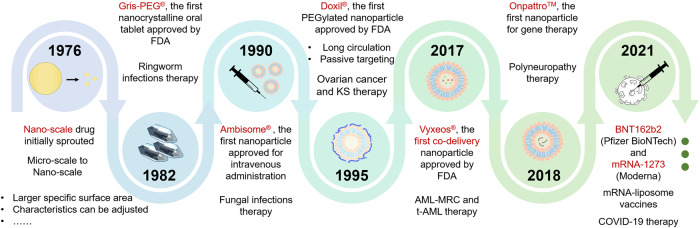
Timeline mapping the historical development and advancement of multifunction NPs. Parts of the figure were drawn using Servier Medical Art licensed under a Creative Commons Attribution 3.0 Unported License (https://creativecommons.org/licenses/by/3.0/)

Multifunctional NPs used in drug delivery has grown by leaps and bounds in recent decades (Fig. 3) due to their advantages, such as improving drug solubility and penetration and reducing drug dosage and side effects. In the early 1970s, scientists realized that intravenous injection of drug suspensions with a particle size of tens of microns was hazardous for embolism.^20^ In 1976, Peter, the pioneer of the concept of NPs, first reported NPs. This concept inspired the researchers, allowing drug therapy a qualitative leap from micro- to nano-scale.^21^ In particular, this progress is not only a change in particle size. Compared with micron-sized particles, NPs have a larger specific surface area, and the characteristics of materials used to construct particles can be adjusted according to the nanoscale size and shape of NPs.^22^ Traditionally, NPs are defined as ultra-dispersed solid supramolecular structures with particle sizes usually smaller than 500 nm; and if it is too large, it is quickly cleared by the reticuloendothelial system (RES). However, it is worth noting that too small particles (usually below 10 nm) are rapidly excreted by the kidneys.^23^

At the early stage, the approved NPs were mainly used to treat liver diseases or infectious diseases because they predominantly accumulated in the liver or were uptaken by the RES. The groundbreaking precedent of nano-formulation is the NP-based nanocrystalline oral tablet, Gris-PEG^®^, marketed in 1982 for treating ringworm infections. The maximal plasma concentration of griseofulvin increased by twice due to the release enhancement. In 1990, the first liposomes (Ambisome^®^) were permitted to treat fungal infections.^24^ Two other liposomes, Epaxal^®^ and Abelcet^®^, were launched to treat hepatitis A and invasive severe fungal infections, respectively, following five years.^25,26^ In 1995, a new liposomal formulation, PEGylated doxorubicin liposomes (Doxil^®^), was launched. PEGylated modification allows reduced serum attachment and RES uptake and prolonged blood circulation time and strengthens passive targeting and EPR effect to treat cancer.^27,28^ Likewise, Oncaspar^®^, L-asparaginase pegylated enzyme NPs, was approved to combat acute lymphocytic leukemia.^29^ After then, researchers began to design various multifunctional NPs, such as conjugating ligands for active targeting and incorporating/surface-wrapping temperature-sensitive, pH-sensitive or photosensitive polymers in NPs for responsive release.^30,31^ Numerous NPs were reported in the past twenty-five years, yet few have been translated. Nonetheless, the NP application had a breakthrough recently, demonstrated by the approval of the co-loaded liposome Vyxeos^®^ in 2017, LNP (Onpattro^TM^) in 2018^19^ and the LNP COVID-19 vaccine (mRNA-1273 and Comirnaty^®^) in 2021. Launching Onpattro^TM^ is a critical milestone for nucleic acid delivery using NPs.^32^ So far, more than 90 nanomedicine have been approved for clinical use, indicating the bright application potential of NPs.^33^ Given the breakthrough in drug delivery, NPs are demonstrating increasing attention in combination therapy and are considered a potent tool to improve the combined treatment.

## The models for evaluating combination effects

Combining multiple drugs may cause additive, synergistic, or antagonistic effects, representing similar, greater, or lesser responses compared to the individual drugs.^34^ Two or more drugs work together on a complex biological network rather than one target to achieve synergistic treatment.^35^ Predominantly, the synergistic effect obtains through pharmacodynamic (PD) or pharmacokinetic (PK) interactions.^36^ PD synergy refers to the therapeutic outcome of drug combination by targeting different pathways, such as enzymes, substrates, metabolites, ion channels, signaling cascades, etc.^37^ Asbjørn et al. reported a general pharmacodynamic interaction model (GPDI) to assess docetaxel-SCO-101 combination synergy.^38^ They concluded that GPDI could quantify the interaction through maximal effects and potency. GPDI demonstrated that the combination enabled 60% potency increase against drug-resistant MDAMB-231 TNBC cells compared to docetaxel. Gabriel et al. also found that cytarabine synergied with the WEE1 inhibitor (adavoxetine) through PD interaction. The two drugs acted on leukemia cell-related metabolite pathways, such as gluconeogenesis, amino acids, nucleotides, glutathione and electron transport.^39^ PK synergy refers to affecting the absorption, bioavailability, distribution or metabolism of drugs through interaction. For instance, oral administration of taxane isolated from Taxus chinensis (a mixture of various pharmaceutical ingredients containing 17.2% paclitaxel (PTX)) could significantly increase the concentration and systemic exposure of PTX in rat blood and extend the drug’s retention.^40^ The underlying synergy mechanisms may result from the “herbal compatibility” that could compromise the activity of P-gp and CYP3A4. Always, NPs allow synergistic effects by several factors, such as improving solubility, PK consistency and diseased-site accumulation of two drugs.^41^ E.g., cytarabine and daunorubicin in liposomal combination Vyxeos® demonstrated modest differences in PK performance while exhibiting significant differences in free combination.^42^

**Fig. 4:**
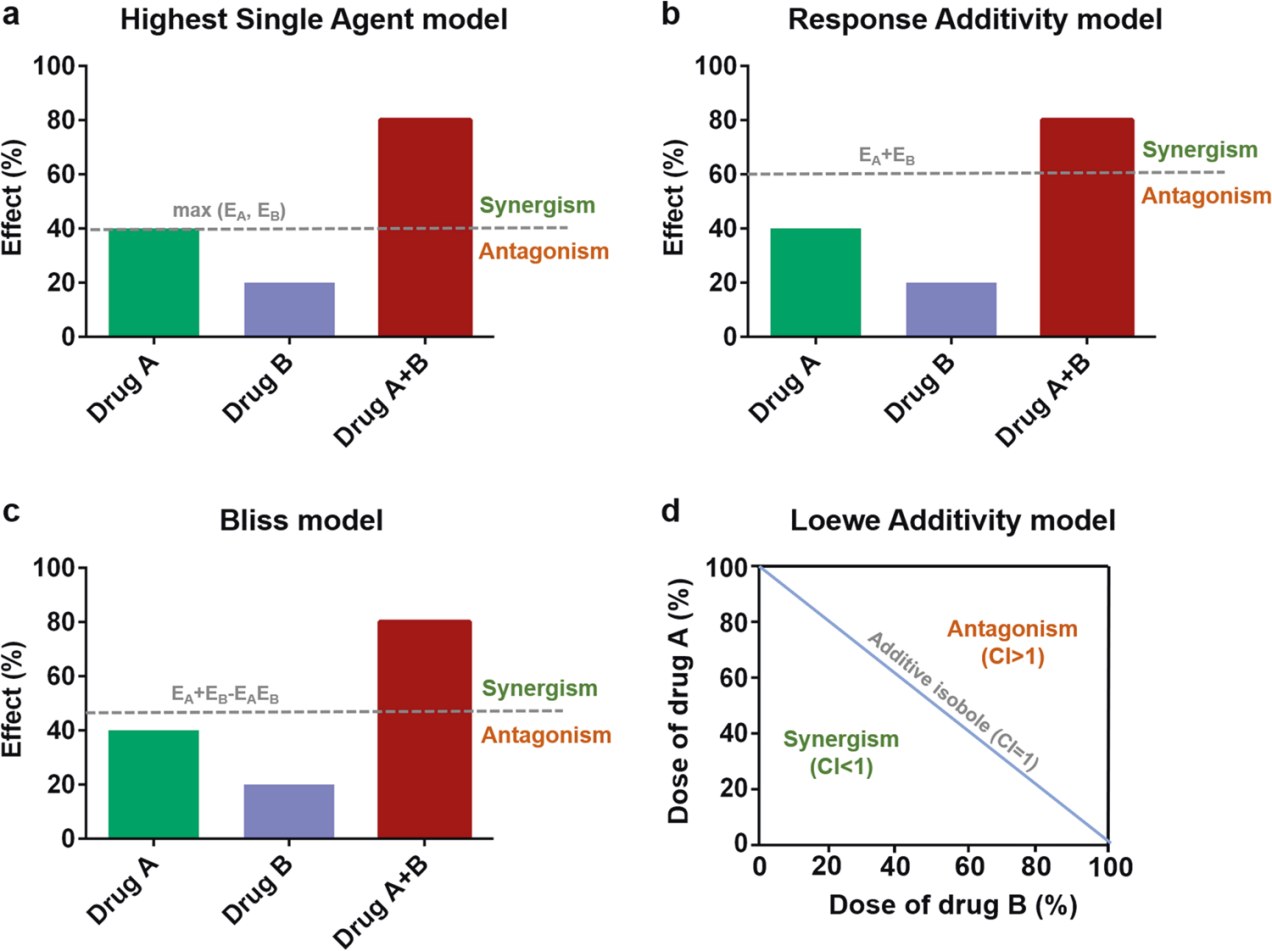
Schematic diagram of the models for evaluating combination effects. Effect-based models: **a** Highest Single Agent model : CI = max (EA, EB)/EAB, the significance of a positive combination is given by the *P* value of the statistical test compared to the HSA. **b** Response Additivity model : CI = (EA + EB)/EA, the drug combination is positive when EAB is greater than the sum of the individual effects EA and EB. **c** Bliss Independence model: CI = (EA + EB – EAEB)/EAB, the drug combinations based on the assumption that drugs act independently on distinct action sites. d Concentration-based model: **d** Loewe Additivity model : CI= a/A + b/B, this flexible model provides isobol representation in addition to the algebraic analysis

Usually, the combinatory effect is evaluated by measuring the combination index (CI) that indicates a synergistic (CI < 1), antagonistic (CI > 1), or additive (CI = 1) combination.^43^ Patients may experience significant toxicity if a multi-component combination is not carefully and accurately examined. There are sets of reference models based on different mathematical principles that have been developed to corroborate the benefits of drug combinations over their monotherapies.^44^ Those approaches can be divided into effect-based and concentration-based models (Fig. 4).

Effect-based methods, such as the Highest Single Agent (HSA), Response Additivity and Bliss Independence models, directly compare the response EAB resulting from the combination of two drugs, respectively named A and B, administered at doses of a and b to their individual effects EA and EB.^45^ The HSA model calculates CI by the formula: CI = max (EA, EB)/EAB, and the significance of a positive combination is given by the *P* value of the statistical test compared to the HSA. The Response Additivity model assumes that a drug combination is positive when EAB is greater than the sum of the individual effects EA and EB. CI can be calculated as CI = (EA + EB)/EAB. However, this strategy assumes that drugs have linear-dose–effect curves which is not the general case. The most popular effect-based model is the Bliss independence model.^46^ Bliss model evaluates the drug combinations based on the assumption that drugs act independently on distinct action sites but lead to a typical result. The CI is calculated as CI = (EA + EB – EAEB)/EAB. However, it presumes that the drugs have exponential dose–effect curves, which may result in misleading interpretations.^43^ Also, it does not take into consideration drug interactions.

In contrast, concentration-based methods predict the effects of drug combinations based on their non-linear dose-response curves and assume that the effects of the combined drugs are additive but not necessarily independent. The Loewe additivity model is the most widely used dose-based strategy (Fig. 4). The CI is calculated as CI= a/A + b/B. This flexible model provides isobol representation in addition to the algebraic analysis. Nevertheless, dose-based models require large amounts of data which might be expensive or difficult to get^45^. The zero interaction potency model was recently proposed as a hybrid approach between the Bliss and the Loewe Additivity models to evaluate drug combinations.^45^

Overall, each model has advantages and limitations, and the choice of model depends on the characteristics of the drug and the target illness. The investigation of drug combinations requires different approaches since no reference model appropriate for all biomedical applications is available so far. Numerous software based on different models has been developed, such as CompuSyn, CalcuSyn, Synergyfinder, COMBIA, and Combenefit.^46^

## Cancer

Cancer is a heterogeneous disorder stamped by the undistinguishable growth and the proliferation of abnormal cells, causing a patient’s death. Solid tumors comprise stromal cells (including fibroblasts and inflammatory cells), cancer cells, and infiltrating immune cells impacted in an extracellular matrix and nourished with a vascular network.^47–49^ The first-line treatment approach for most cancers is chemotherapy.^50^ Although conventional chemotherapies can elevate patient survival rates, they also possess various restrictions, e.g., drug-resistance development, disproportionate toxicity, little targeting, and unwanted side effects. Since the first four-drug combination therapy was approved in 1964, many studies confirmed that drug combination could improve the treatment outcomes, such as suppressing tumors and prolonging patient survival. Additionally, amid some new treatment strategies, nanotechnology is playing an increasing role in encompassing treatment&diagnosis, identifying biomarkers, and understanding cancer progression.^51–54^

### Targets for cancer therapy

**Fig. 5:**
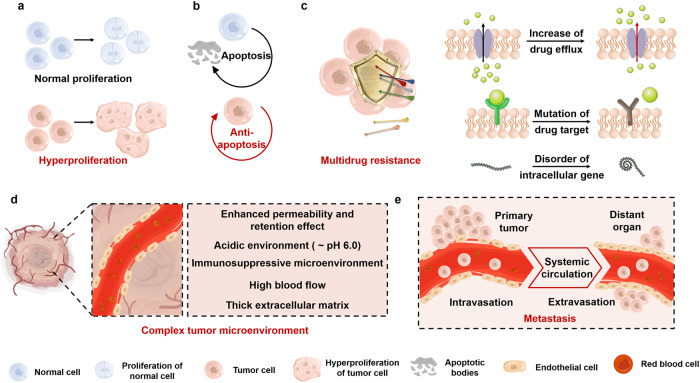
Schematic illustration of pathological features of tumor and therapeutic approaches against cancer. **a** Hyperproliferation. Compared with normal cells, the proliferation rate of tumor cells is greatly increased. **b** Anti-apoptosis. The cell cycle of normal cells includes an apoptotic phase, whereas the anti-apoptotic ability of tumor cells promotes their unlimited proliferation. **c** Multidrug resistance. Tumor cells achieve multidrug resistance by increasing drug efflux, mutating drug targets, and disordering intracellular genes. **d** Tumor-specific microenvironment includes enhanced permeability and retention effect, acidic environment, immunosuppressive microenvironment, high blood flow and thick extracellular matrix. **e** Metastasis. Tumor cells can migrate to distant tissues through systemic circulation, leading to cancer metastasis. Parts of the figure were drawn using Servier Medical Art licensed under a Creative Commons Attribution 3.0 Unported License (https://creativecommons.org/licenses/by/3.0/)

In as much as monotherapy treatment is often used to treat cancers, combinatorial treatments targeting specific cell-sustaining and cancer-inducing pathways are the mainstays and most efficient.^55,56^ Traditional chemo-based monotherapy treatments usually damage cancerous and healthy cells since chemotherapy targets all proliferating cells. Also, conventional monotherapeutic techniques can be highly toxic and significantly compromise patients’ immune systems, increasing their disease susceptibility.^57,58^ Nevertheless, combining therapy can actively target tumors and their microenvironment by disrupting different signaling proteins contributing to cancer’s initiation and sustaining (Table 1). These pathways are essential in cancer, intertwined with refractory characteristics that lead to excessive tumor growth, decreased tumor cell apoptosis, drug resistance, metastasis and tumorigenesis (Fig. 5).^5,59,60^

**Table 1:** Clinical research on combining and co-delivering strategies for cancer

Combining or co-delivery drugs	Duration	Patient numbers	Efficacy	Study Phase	References	Additional information
Prednisone + Abiraterone Acetate vs. Prednisone + Placebo	3.8 years	Abiraterone Acetate (*n* = 143) Placebo (*n* = 71)	Significantly prolonged the time to PSA. (*P* = 0.0002)	Phase 3	–	NCT01695135
Docetaxel + Sunitinib vs. Docetaxel	2.8 years	Sunitinib (*n* = 296) Docetaxel (*n* = 297)	Significantly increased the percentage of participants’ objective responses with CR and PR. (*P* = 0.0018)	Phase 3	–	NCT00393939
ADT + Abiraterone Acetate + Prednisone vs. ADT + Placebo	5.4 years	Combine (*n* = 597) Placebo (*n* = 602)	Significantly improved PFS and OS. (*P* < 0.0001)	Phase 3	^485,486^	LATITUDE NCT01715285
Lapatinib + Trastuzumab vs. Lapatinib	4.5 years	Trastuzumab (*n* = 148) Lapatinib (*n* = 148)	Prolonged PFS, improved or maintained near-term HRQOL, 4.5-month median OS.	Phase 3	^487,488^	EGF104900 NCT00320385
Anastrozole + Fulvestrant vs. Anastrozole	4 years	Fulvestrant (*n* = 349) Anastrozole (*n* = 345)	Increased long-term survival.	Phase 3	^489^	NCT00075764
Erlotinib + Sunitinib vs. Erlotinib + Placebo	18 weeks	Sunitinib (*n* = 13) Placebo (*n* = 17)	Well tolerated	Phase 2	^490^	–
PD-1/PD-L1 inhibitor + Lung Cancer Fang No. 1 vs. PD-1/PD-L1 inhibitor	3.2 years	*n* = 40	Decreased tumor markers, and elevated immune level (*P* < 0.05). 22.5% increase in DCR.	–	^491^	–
Gemcitabine and Cisplatin + Bevacizumab vs. Gemcitabine and Cisplatin	2 years	*n* = 50	The total effective rate increased by 20%, the two-year survival rate increased by 22%, and the incidence of adverse reactions decreased.	–	^492^	–
Azacitidine + Ivosidenib vs. Azacitidine + Placebo	2 years	Ivosidenib (*n* = 72) Placebo (*n* = 74)	Significantly increased event-free survival. (*P* = 0.002)	Phase 3	^493^	NCT03173248
Nab-Paclitaxel + Atezolizumab vs. Nab-Paclitaxel + Placebo	2 years	*n* = 451	Significantly prolonged PFS (*P* = 0.002)	Phase 3	^494^	NCT02425891
Liposome formulation of irinotecan and floxuridine CPX-1	28 days	*n* = 33	Anti-tumor efficacy against advanced solid tumors	Phase 1	^495^	–
CPX-351: Daunorubicin and Cytarabine liposomes vs. 7 + 3: Daunorubicin and Cytarabine	Treatment period 30 days; follow-up 5 years.	CPX-351 (*n* = 153) 7 + 3 (*n* = 156)	After 5-year follow-up, the improved overall survival with CPX-351 vs. 7 + 3	Phase 3	^19,100,496^	NCT01696084
Carboplatin + Paclitaxel + Veliparib vs. Carboplatin + Paclitaxel + Placebo	4 years	Veliparib (*n* = 765) Placebo (*n* = 375)	Significantly prolonged PFS. (*P* < 0.001)	Phase 3	^497^	NCT02470585
Nivolumab + Ipilimumab vs. Ipilimumab or Nivolumab	5 years	Combine (*n* = 313) Ipilimumab (*n* = 311) Nivolumab (*n* = 313)	Combine showed superior OS at 5 years, PFS, and ORR, with a better safety profile than other groups.	Phase 3	^498,499^	NCT01844505

*PSA* prostate-specific antigen progression, *CR* complete response, *PR* partial response, *ADT* androgen deprivation therapy, *PFS* progression-free survival, *OS* overall survival, *HRQOL* health-related quality of life, *DCR* disease control rate, *CPX-351* co-loaded liposomes of daunorubicin and cytarabine with a 1:5 molar ratio; 7 + 3, a routine of 7-day cytarabine and 3-day daunorubicin, *ORR* objective response rate

#### Hyperproliferation pathways

Autocrine growth factors are effector substances commonly found in cancers. These growth factors enhance malignant characteristics through pro-proliferation activities *via* the assistance of autocrine growth loops.^61^ Amongst the numerous growth factors, the most prevalent and major ones in cancers include epidermal growth factor, insulin-like growth factor-2, tumor-growth factors, 5-hydroxytryptamine, and vascular-endothelial-growth factor (VEGF), etc.^61–64^ Cancer may arise due to extreme proliferation if these factors cannot answer the deleterious controlling indicators.

Stimulated by growth factors, tumor cells initiate kinase-mediated signaling events to increase nutrient uptake, including glucose, amino acid, and lipid. Due to the large influx of glucose into proliferating cells, only a small fraction of glucose is fully oxidized in the normal tricarboxylic acid cycle. The remaining glucose is converted to lactate through glycolysis and secreted, resulting in an acidic and hypoxic tumor microenvironment (TME).^65,66^ This characteristic provides a basis for the design of pH-sensitive and reactive oxygen species (ROS)-sensitive DDSs. Cells have adapted a systemic pathway to deal with high oxidative intrinsic and extrinsic stress *via* an antioxidant response termed the Nrf2-Kelchlike ECH-related protein-1 (Keap1) signaling.^67^ Keap1 is an oxidant sensor and electrophile, which gradually promotes Nrf2 degradation under dormant conditions. Nrf2 is vigorously located in the nucleus to induce an anti-oxidative reply in intense oxidative pressure because of reactive oxygen species or the build-up of carcinogens.^68^ Tumorigenicity is regulated by two means of the Nrf2 antioxidant reply, either *via* Keap1-dependent and Keap1-independent mechanisms or *via* stimulating the development and cancer-cell survival, which are already inducted since Nrf2 and the anti-oxidative reply aids tumors in dealing with oxidative stress.^69^ Hence, the Nrf2 and its anti-oxidative response could be a suitable target for combinatorial therapy. At the same time, mitochondria are the central organ of cell metabolism. ROS or metabolic enzymes, i.e., α-ketoglutarate-dehydrogenase, pyruvate dehydrogenase and glycerol-3-phosphate dehydrogenase, can be the targets for regulation.^70,71^

#### Anti-apoptotic pathways

Apoptosis is defined as programmed cell death in the human body. Two key apoptosis pathways occur in humans, the intrinsic and the extrinsic.^72^ B-cell lymphoma 2 (Bcl-2) protein, a member of the Bcl family, enables cell proliferation by constraining adaptors that are needed for apoptosis motivation and caspase cleavage, inducing the nuclear and cell fragmentation that is apoptosis characteristics.^73^ A study indicated that Bcl-2, utilized as a prognosis indicator in non-small-cell lung cancer, correlated with unfavorable histology in neuroblastoma and overexpression in prostate cancer.^74,75^ So, the researchers claimed that treatment strategies targeting these anti-apoptotic or pro-survival proteins could escalate anticancer efficacy. The extrinsic way contains various signaling proteins, such as death receptors and ligands, APO-1/Fas (CD95), tumor necrosis factor-alpha (TNF-α)/TNFR1, Apo3L/DR3, Apo2L/DR4, and Apo2L/DR5 that are parts of the TNF gene superfamily. These death receptors activate intracellular signaling, split and stimulate caspase-3 and -8, causing apoptosis.^76,77^

#### Drug efflux pathways

Cells can also efflux drugs after ingesting them. The efflux is mainly refereed by the ATP-binding cassette (ABC) transporter family. Eliminating the use of ATP-driven energy by cytotoxic agents and targeted anticancer drugs could combat the excretion of drugs from cancer cells. Over ten human ABC superfamily transporters have been identified, of which nearly 50 members have been divided.^78,79^ P-glycoprotein (P-GP/ABCB1), the first member of this family to be identified in the mid-1970s, is the glycoprotein responsible for regulating drug permeability. In addition, the structures and functions of a series of efflux proteins represented by multidrug resistance-related protein 2 (MRP2/ABCC2) and breast cancer resistance protein (BCRP/ABCG2) have become increasingly clear.^80^ According to the structures of different ABC superfamily transporters, finding their natural inhibitors or designing new chemical structures for competitive inhibition is the first choice to reduce drug efflux. The deeper cycle pathways of cells can jointly regulate it, but it must be ensured that these regulators can precisely fight tumor cells and reduce the threat to the healthy ones.

#### Immune checkpoints and cytokines

Contrary to conventional immune system function, the immune system shows a catalytic character in cell carcinogenesis’s initiation and transformation stages. For the dysfunction of the immune system, the first-generation target that has achieved clinical application is the immune checkpoints.^81^ T cells play the most crucial role among the various immune cells infiltrating tumor sites. Naive T cells examine the microenvironment and are activated when recognizing tumor antigens. After proliferating and differentiation, they can attack and destroy cells expressing the relevant antigens.^82^ However, this processing pathway is highly complex and involves many reverse inhibitory molecules, including immune checkpoints.^83,84^ Two immune checkpoints achieved clinical application are CTLA-4 and PD-1, interfering with co-stimulation and T-cell antigen receptor-mediated signaling, respectively.^85^ Immune checkpoint inhibitors, alone or in combination, can improve the suppressive effect of the tumor environment on T cell production, restoring immunosuppression and achieving effective treatment.

Unlike immune checkpoints, cytokines directly control tumor-cell growth through antiproliferative or pro-apoptotic effects and act on tumor cells indirectly by stimulating immune cells. Cytokines include four subclasses of chemokines, interferon (IFN), interleukin (IL), and TNF. IL-2, IFN-α, and TNF are typical examples already used clinically.^86^ However, maintaining their stability is difficult to guarantee because they are small molecular proteins with a molecular weight between 8 to 12 kD.^87^ Moreover, functional carriers are needed to strengthen their delivery to achieve targeting and avoid erroneous activation of normal cells.

### Strategies for combinatorial cancer therapy

Tumors are divided into benign and malignant tumors according to their ability to invade and metastasize. Surgical resection to completely resect the tumor is the main strategy for benign tumors. In contrast, the treatment selection of malignant tumor relies on the disease-developing stage. Surgical treatment that can radically resect local lesions is often utilized for the early stage.^88^ Drug chemotherapy or radiotherapy serves as an adjuvant therapy, depending on pathological staging, immunohistochemistry results and lymphatic metastasis.^89,90^ In addition, precision therapies, such as biological immunotherapy, gene therapy and targeted therapy, can be combined to control cancer development without causing damage to normal tissues.^91^

#### Inhibiting proliferation and promoting apoptosis

Liposome-mediated DDS is the most commonly used multifunctional carrier to alleviate tumor cell hyperproliferation and anti-apoptosis. Liposomes possess the particle-size advantage shared by nanocarriers and can passively target tumor sites through the enhanced permeability and retention (EPR) effect across the hyperproliferative tumor vascular epithelium.^92^ Liposomes have higher biocompatibility and efficiency and lower immunogenicity than inorganic NPs.^93^ Moreover, liposome-based smart DDSs, such as pH- and temperature-sensitive liposomes, have been shown to promote the controlled and sustained release of drugs to targeted sites and enhance the pharmacodynamic and pharmacokinetic profiles of therapeutic payload with little toxicity.^94,95^ Various liposomal products, such as Myocet, Doxil, Lipo-dox, DaunoXome and Marqibo, were initially marketed for treating cancer. These liposomal preparations encapsulate DNR, doxorubicin (DOX) and vincristine sulfate individually.^19,96,97^ Notably, a co-delivery liposomal formulation containing DNR and ara-C (Vyxeos^®^) with a 1:5 molar ratio was approved for clinical use (Table 1). The formulation demonstrates a substantial anti-leukemia outcome with tolerable toxicity in patients of a wide range of ages suffering from acute myeloma leukemia, indicating the rationality of the combination therapy.^98–100^ Specifically, Vyxeos^®^ demonstrated over a 6-week therapeutic effect, twofold longer than the ordinary cocktail combination.^101^ The enhanced treatment effects were predominantly ascribed to prolonged half-life and specific uptake. The uptake of the drugs by leukemia cells is increased by 2–9 fold compared to the normal bone marrow cells.

The co-delivery NPs often improve the drugs’ cytotoxicity to tumor cells compared with the cocktail combination. Whereas “Guard” drugs in combination with another cytotoxic drug can modulate the dose to achieve different treatment effects using the small distinctions between normal and cancerous cells^102,103^. For instance, DOX, a p53 inducer, has significant cytotoxicity at a high dose, while a low dose of DOX triggers G1-G2 detention in normal cells.^102^ After DOX “blocks” healthy cells, another cytotoxic drug could precisely kill cancer cells, and this G1/G2 blockade reduces the side effects of the combination therapy on healthy cells.

Besides the co-delivery of multiple chemotherapeutic drugs, liposomes can also co-deliver gene and small molecular drugs. Li et al. designed liposomes to co-delivering VEGF siRNA and etoposide (ETO).^104^ This system inhibits tumor cell proliferation by silencing VEGF gene expression and synergistically kills tumor cells through the pro-apoptotic effect of ETO. In particular, the co-delivery system wrapped a polymer coating of PEGylated histidine-grafted chitosan-lipoic acid on the surface of cationic liposomes, allowing negatively charged and improving the stability in blood circulation. Whereas this coating was triggered by the acidic environment of the tumor site, enabling the liposomes to have a positive charge and improve penetration and lysosomal escape. The combined delivery system allowed drug protection tumor-cell targeting and significantly inhibited tumor growth and angiogenesis compared with other controls. This modification might provide a direction for traditional gene-associated co-delivery systems that commonly suffer side effects due to electropositivity.

ROS in TME, a class of highly bioactive molecules that act as second messengers in cell signaling and regulate growth factors, is crucial for various tumor biosynthetic processes.^105^ Accumulating evidence indicates that ROS possesses dual roles in cells as oncogenic and antiproliferative factors in the cancer-progress stage.^106^ At the early stage, oxidative stress (OS) initiates the pathological transformation of the physiological signaling network to induce cell oncogenic mutations; at the later stage, they drive cell proliferation by promoting the mitotic signaling cascade; when the tumor progresses to the advanced stage, ROS exceeds the critical value, promoting DNA double-strand breaks and the 8-oxodG formation and leading to apoptosis.^107,108^ Despite the paradox, the ROS pathway still provides a strategy for developing safe and effective anticancer therapies.^109^ As a result, targeting ROS in tumor cells using responsive drug delivery systems is a commonly reported approach. The ROS level in tumor cells is approximately 10-fold higher than the normal cells.^110^ Tang et al. reported reduction-sensitive cleavable PEG and octa-arginine (R8)-modified liposomes (CL-R8-LP) to co-deliver DOX and the P-gp inhibitor verapamil (VER). The PEG coating helped maintain the NP stability and prolong blood circulation. After entering the tumor cells, the ROS broke the disulfide bond, exposing the positive charge of R8 and facilitating aggregation, lysosome escape and intracellular drug release; finally, the intracellular VER inhibited nuclear P-gp-mediated drug efflux transport and improved nucleus delivery of DOX, killing cells by inducing apoptosis and necrosis. CL-R8-LP showed higher DOX cellular uptake efficiency and synergistic antitumor effect with reduced toxicity in MCF-7/ADR and MCF-7/ADR tumor cells.^111,112^ Recently, Wang et al. found that co-loading a ROS-stimulated paclitaxel (PTX) prodrug and a low-molecule weight PD-1/PD-L1 suppresser (BMS-202) into the liposomal cores enabled superior tumor-targeting a ROS-triggered PTX release and prolonged release of BMS-202 after cell entering.^113^ The liposomal formulation demonstrated promising chemo-immunotherapy due to the time-differentiated treatment of the two drugs.^113^ ROS-mediated pro-oxidative therapy is another potential strategy against cancer, elevating intracellular ROS to a toxic threshold and activating ROS-induced cell death pathways.^114^ For instance, Yuan et al. reported a ROS-responsive cinnamaldehyde (CA)-based poly(thioacetal). The polymer contained ROS-responsive thioacetal (TA) and ROS-producing CA and could self-amplify chain-shattering polymer degradation. The endogenous ROS as a triggering agent accelerated TA chain cleavage and CA release, generating additional ROS by disrupting mitochondrial function and inducing rapid polymer degradation. Modifying the polymer using DOX could enhance chemo-immunotherapy by collaboratively amplifying tumor cells’ oxidative stress and immunogenic cell death (ICD).^115^

**Fig. 6:**
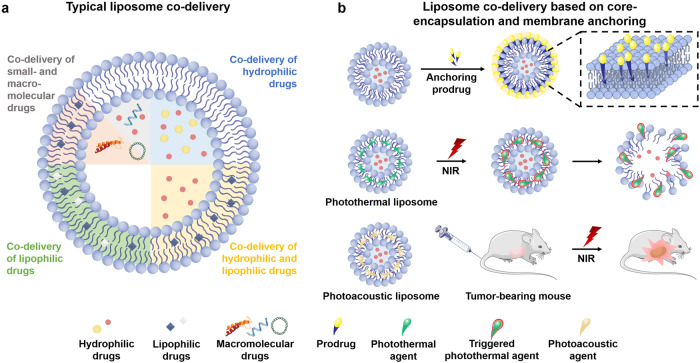
Liposome-based co-delivery. **a** Typical liposome co-delivery loading drugs in cores or lipid membranes. **b** Liposome co-delivery based on core-encapsulation and membrane anchoring. One drug is loaded in the aqueous cores, while other active compounds, e.g., prodrug and photothermal agents, could be anchored on the liposomes through various interaction forces, such as H-bonding, hydrophobic force and *π*-*π* stacking. Parts of the figure were drawn using Servier Medical Art licensed under a Creative Commons Attribution 3.0 Unported License (https://creativecommons.org/licenses/by/3.0/)

Noticeably, anchoring a prodrug, such as hyaluronic acid (HA)-PTX, HA-oridonin and cholesterol-mitoxantrone, onto drug-loaded liposomes may represent a potential approach to improve the tumor targeting for combination therapy.^116–119^ E.g., by anchoring HA-PTX prodrug onto thermosensitive liposomes loading with a water-soluble MMP inhibitor marimastat into the aqueous cores, dual-targeted thermosensitive NPs were developed for targeting tumor cells and the TME.^117^ The results indicated that surface anchoring improved liposome drug-loading ability and elevated liposome’s targeted ability to the tumor cells and MMP-2 by the local thermal treatment. Similarly, HA-oridonin prodrug was anchored onto the checkpoint blockade (anti-CTLA)-loaded thermosensitive liposomes for combinatorial cancer therapy *via* targeting cancer cells and regular T cells. The data showed that the co-delivery boosted anti-tumor immunotherapy by lessening immune suppression of cancer cells and lymphocyte activation. Furthermore, the mechanism study revealed that the two drugs acted synergistically by decreasing cancer-cell THBS1 secretion and breaking THBS1-CD47 interaction.^120^ In addition, liposomes combining photothermal or imaging compounds with drugs were extensively reported for cancer diagnosis and treatment (Fig. 6).^121–124^

Antibody-drug conjugates (ADCs) composed of antibodies, linkers and payloads, are another promising approach for combinatorial cancer therapy.^125^ ADCs act like a bullet, directing cytotoxic drugs to malignant tumors while sparing normal tissue.^126^ Since the first ADC drug was approved in 2000, 14 and over 80 ADCs have been marketed and are under the clinical trial phase, potentially affecting the direction of cancer treatment.^127–129^ Commonly used payloads include microtubule inhibitors, DNA damaging agents and DNA transcription inhibitors. Microtubules target rapidly dividing cells and are more effective at inhibiting cell proliferation. The DNA-related agents target the nucleus DNA and induce apoptosis. RC48 is a human epidermal growth factor 2-ADC (HER2-ADC) consisting of Hertuzumab, olestatin derivatives and a cleavable linker.^130^ The ADC targets HER2 antigens on cancer cells with high specificity and enters cells through clathrin and caverin internalization. The liner cleavage in the cytoplasm or lysosomes allows olistatin release into the cytoplasm, terminating the cell cycle and inducing apoptosis of tumor cells. RC48 indicates more potent cytotoxicity at low concentrations and higher efficacy and safety in treating gastric- and breast cancers by adopting random and uniform cysteine coupling, compared with other HER2-ADCs.^131^ Calicheamicin is a highly cytotoxic DNA-damaging agent that causes the release of toxic catabolites due to its acid instability.^132^ Wiedemeyer et al. designed a calimycin linker drug LD19.10 conjugated to a SEZ6-targeting antibody (ABBV-011) to treat non-small cell lung cancer. They found that ABBV-011 was stable within 14 days in the PDX mouse model and could interact with the minor groove of DNA molecules and induce DNA fission, reducing off-target toxicity.^133^ Increasing ADCs enter clinical trials because of their extended serum half-life and practical efficacy. However, given the side effects caused by the off-target and premature drug release, exploring reliable connection key technology and DDSs involvement may overcome the drawbacks.

#### Reversing multidrug resistance (MDR)

MDR is a critical hindrance in cancer treatment and is induced by multiple factors, such as increased efflux of drugs, mutation of drug target proteins, and intracellular gene disorders. NP-codelivery therapy is promising to alleviate MDR *via* targeted delivery, simultaneously affecting two or more signal pathways.^134–136^ Polymer-based NPs are frequently used to improve the co-delivery and combat MDR. Overexpressing the drug efflux transporter P-GP significantly contributes to MDR. A recent report indicated that CA XII cooperates with P-GP secretion in drug-resistant cancer cells to exert drug resistance.^137^ The results displayed that the CA XII inhibitors, either small molecules or antibodies, significantly inhibited cell resistance when combined with chemotherapeutic agents targeting P-GP substrate therapy. The cocktail administration often leads to asynchrony therapeutic effects because of the differences in the physicochemical features of drugs and the pharmacokinetic alterations. Chen et al. designed cationic core-shell NPs to co-deliver DOX and pDNA using amphiphilic chitosan derivatives.^138^ They found that the low-dose co-loaded DOX increased the pDNA transfection efficacy by 74% in T293 cells, likely owing to DOX’s activation of nuclear factor-κB (NF-κB). However, the dosing with high DOX doses allowed significant cytotoxicity rather than the synergistic effect on promoting transfection. As a result, the drug proportion in the co-delivery system is essential to the synergy.

The order and timing of drug delivery also affect efficacy against MDR cancer due to the complexity of signaling pathways. For example, in advance, ligating the apoptotic signaling network by erlotinib, an EGFR kinase inhibitor, significantly enhanced the ability of a DNA damage-inducing agent (DOX) to kill cancer cells.^139^ For the RNA/drug co-delivery, the P-gp inhibition by RNA needs to work in advance.^140^ Lee et al. reported a light-responsive mesoporous silica nanoparticle (PMSN) for sequential release P-gp short-hairpin RNA (shRNA) and photocaged prodrug of DOX stimulated by external light, which shRNA anchored onto PMSN and DOX was loaded into the inner pores.^141^ They found that the intracellular release of shRNA and DOX could be controlled by 405 and 365 nm light irradiations that allowed specific cleavage of coumarin and o-nitrobenzyl ester. The results indicated that the co-delivery could extend drug retention and improve chemotherapeutic effects against MDR liver cancer.

#### Inhibiting tumor metastasis

Metastasis, an essential hallmark of cancer death, leads to the development of secondary tumors because of the failure of tumor cells to be killed entirely at the original primary tumor site^142^. Immunotherapy can detect and monitor disseminated- and circulating tumor cells more accurately than chemotherapy for primary tumors, showing its great potential in treating metastatic tumors. Immunomodulatory therapies, such as tumor vaccines, cytokines and immune checkpoint blockers, have been approved for treating over 50 cancer types.^143–145^ Always combined formulation is required for cancer immunotherapy. NP-based co-delivering immuno-stimulatory components and antigens represent a promising immunotherapy regime, owing to its ability to stimulate an immune reply of antigen-targeting. Sun et al. developed a cyclodextrin-based nanoformulation co-loaded with ginsenoside Rg3 and quercetin.^146^ This nanoformulation synergistically induced ICD and changed “cold” tumors into “hot” ones.^147^ In vivo results showed that its combination with anti-PD-L1 prolonged the median survival time of mice over twice and effectively inhibited liver metastasis compared to monotherapy. Moreover, NPs can simultaneously load with several drugs, potentially stimulating multiple immune pathways. Recently, a mesoporous silica NP vaccine, encapsulating tyrosinase-related protein 2 (TRP2) peptide and two different toll-like receptors agonists (CpG oligonucleotide and monophosphoryl lipid A), was reported to treat B16 melanom.^148^ The mesoporous silica NPs protected the peptide TRP2 from decomposition and delivered the three ingredients to dendritic cells, provoking effective TRP2-specific CD8^+^ T cell responses. The study in vivo indicated that the vaccine could attenuate lung metastasis and prolong the animals’ median survival rate *via* comprehensively regulating host immune responses linking CD4^+^ and CD8^+^ T cells and macrophages.

#### “Drug-repositioning” strategy

“Drug repositioning” is a popular therapeutic approach in cancer therapy.^149^ Exploring the potential of non-cancer-treated drugs for cancer treatment may help improve the cancer therapy regime because the drug candidates have acceptable safety and identified pharmacokinetic profiles.^150^ The rapid high-throughput development enables the omics data to grow exponentially and significantly promote drug repositioning on cancer.^150–152^ For instance, aspirin is commonly used for anti-inflammation and antiplatelet action; however, several studies have identified its potency in preventing and treating various cancers.^153^ Wang et al. designed chitosan NPs co-loading with 5-fluoropyrimidine (5-Fu) and aspirin.^154^ They found that non-toxic aspirin concentrations increased the sensitivity of hepatocellular carcinoma cells to 5-Fu by enhancing the 5-Fu-mediated accumulation of cells in the G1 phase. Meanwhile, aspirin acted collaboratively by suppressing the cyclooxygenase 2 (COX-2)/NF-κB signaling pathway.

## Atherosclerosis (AS)

AS is a cardiovascular disease (CVD) caused by lipid accumulation and other blood components in the arterial intima. The smooth muscle cell (SMC) proliferation and the collagen-fiber growth lead to atheromatous lipid-enriched necrosis injuries, vascular wall sclerosis, and inflammation is demonstrated when the plaque forms.^155^ Various CVDs can be caused by AS, such as peripheral vascular disease, coronary artery disease, ischemia, and stroke^138,156^. Several factors, such as hypercholesterolemia, hyperhomocysteinemia, hypertension, diabetes mellitus, genetic abnormalities, chlamydia, pneumonia infection, as well as various lifestyles like smoking cigarettes, not exercising regularly, and stress, have been determined to be the major risk factors linked to the AS development.^157,158^

### Targets for AS therapy

Functional and structural alterations in the cell lines, including SMCs, endothelial cells, T-lymphocytes, monocytes/macrophages, foam cells and platelets, lead to the initial development of AS plaques.^159–162^ Sustained high levels of low-density lipoprotein (LDL) infiltration in blood vessels lead to aggregation, the introduction of ROS and immune cells, and the production of pro-atherogenic lesions by LDL particles. Leukocytes adhere to endothelial cells, followed by monocyte extravasation into the intimal space and differentiation into macrophages by platelet factor 4 (CXCL4). Differentiated macrophages take up lipid proteins and disrupt cellular homeostasis to derive lipid-rich foam cells.^163^ Platelet activation and aggregation promote the expression of trending factors CCL5 and soluble CD40L and the release of IL-1β and have the ability to express adhesion factors to form aggregates and secrete inflammatory factors.^164^ In the late stage of AS, inflammatory stimulation promotes the apoptosis of macrophages and produces MMPs, leading to the degradation of the fibrous cap. The increased instability of vulnerable atheromatous plaques, which eventually rupture and form a thrombus, is also a significant cause of ischemic events.^165^ Even though AS occurs in different bodies, the mechanisms before these events are similar. Studies have shown that the core of AS pathogenesis is based on excessive LDL and the resulting other mechanisms, such as oxidative stress, vascular inflammation, and cell proliferation.^166–168^ Statin drugs, cholesterol-lowering compounds, have been widely accepted as an imperative therapy for treating AS.^169,170^ However, their undesirable effects, such as liver damage and muscle pain, make it necessary to develop combining therapies.^171^ Various combining strategies against AS are summarized in Table 2.

**Table 2:** Clinical research on combining and co-delivering strategies against AS

Combining or co-delivery drugs	Duration	Patient numbers	Efficacy	Study Phase	References	Additional information
Aspirin + Rivaroxaban vs. Aspirin + Placebo	3.2 years	Rivaroxaban (*n* = 9152) Placebo (*n* = 9126)	Primary outcome events of CVD occurred in fewer patients in the Rivaroxaban than in the placebo group. (*P* < 0.001)	Phase 3	^500,501^	NCT01776424
Ezetimibe + Bempedoic acid vs. Ezetimibe + Placebo	17 weeks	Bempedoic acid (*n* = 88) Placebo (*n* = 181)	Bempedoic acid reduced LDL-C by 28.5% greater than the placebo group. (*P* < 0.001)	Phase 3	^502^	NCT03001076
Statin + Ezetimibe + Niaspan vs. Statin + Placebo	2 years	*n* = 51	Non-HDL-C was significantly reduced at 12-month triple therapy vs. monotherapy. (*P* = 0.01)	Phase 4	^503^	NCT00687076
Atorvastatin + Ezetimibe vs. Atorvastatin + Placebo	12 weeks	Ezetimibe (*n* = 255) Placebo (*n* = 248)	Decreased LDL-C. (*P* < 0.01)	Phase 3	^504^	–
Evacetrapib + Statins vs. Evacetrapib	12 weeks	Statins (*n* = 41) Evacetrapib (n = 39)	A combination of evacetrapib and statin decreased LDL-C. (*P* < 0.001)	Phase 2	^505^	NCT01105975
Atorvastatin + Lovaza vs. Atorvastatin + Placebo	16 weeks	Lovaza (*n* = 123) Placebo (*n* = 122)	Significantly reduced median non-HDL-C levels. (*P* < 0.001)	Phase 3	^506^	NCT00435045
Cilostazol + L-Carnitine vs. Cilostazol + Placebo	0.5 year	L-Carnitine (*n* = 80) Placebo (*n* = 83)	There was an increase in PWT of 37.9% for L-carnitine, compared with 20.9% for placebo.	Phase 4	^507^	NCT00822172
Bempedoic acid + Ezetimibe vs. Bempedoic acid or Ezetimibe	12 weeks	Combine (*n* = 108) Bempedoic acid (*n* = 110) Ezetimibe (*n* = 109)	Significantly lowered LDL-C. (*P* < 0.001)	Phase 3	^508^	NCT03337308
LMT + Alirocumab vs. LMT + Placebo	62 weeks	Alirocumab (*n* = 209) Placebo (*n* = 107)	(a) A 48% reduction in LDL-C from baseline (pretreatment) to 24 weeks. (*P* < 0.0001) (b) Significant reductions in non–HDL-C, total cholesterol, apolipoprotein B, and lipoprotein. (P < 0.0001) (c) A greater portion of patients achieved LDL-C < 70 mg/dL. (*P* < 0.0001)	Phase 3	^509^	ODYSSEY COMBO I NCT01644175
LMT + Alirocumab vs. LMT + Placebo	89 weeks	Alirocumab (*n* = 1553) Placebo (*n* = 788)	(a) Combination of LMT and Alirocumab reduced LDL cholesterol levels by 62% in high-risk patients. (*P* < 0.001) (b) During the 80 weeks of follow-up, the Combination of LMT and Alirocumab reduced the rate of major adverse cardiovascular events by 48%. (*P* = 0.02)	Phase 3	^510^	ODYSSEY Long Term NCT01507831

Primary outcome event of CVD, death, stroke, or myocardial infarction; LDL-C: low-density lipoprotein cholesterol; Non-HDL-C, non–high-density lipoprotein cholesterol; PWT, peak walking time; LMT, lipid-modifying therapy; The rate of main adverse cardiovascular events includes as follows, nonfatal myocardial infarction, a composite end point of death from coronary heart disease, or unsteady angina needing hospitalization, or fatal or nonfatal ischemic stroke

### Strategies for combinatorial AS therapy

#### Combining therapy strategies

**Fig. 7:**
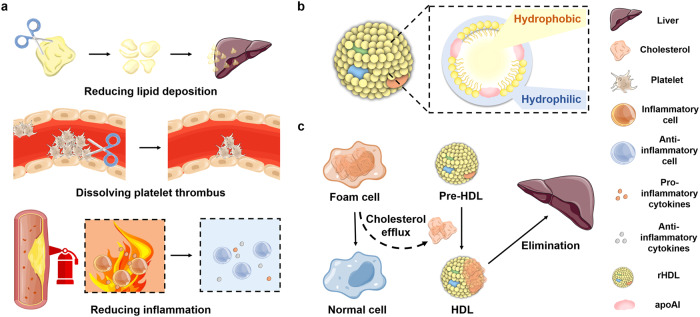
**a** The therapy strategies for AS include reducing lipid deposition, dissolving platelet thrombus and reducing inflammation. **b** The structure of rHDL. rHDL mainly comprises phospholipids and apoAI; the structure includes a hydrophobic core and a hydrophilic shell. **c** RCT process of HDL. Pre-HDL turns into HDL by combining cholesterol, promotes the transformation of foam cells into normal cells, and transports cholesterol to the liver for elimination. Parts of the figure were drawn using Servier Medical Art licensed under a Creative Commons Attribution 3.0 Unported License (https://creativecommons.org/licenses/by/3.0/)

The primary therapy pathways for AS are shown in Fig. 7. Reducing lipid uptake and promoting cholesterol efflux are the most direct procedures to delay AS progress and development.^168^ Statins could effectively inhibit cholesterol absorption, lower LDL levels, prevent AS progression, and reduce cardiovascular event risk.^172–174^ Many recent investigations focused on statin-combination therapy. The ezetimibe-statin combination strategy is the most commonly used (Table 2). Ezetimibe is a Niemann-Pick C1-like 1 inhibitor that inhibits cholesterol absorption in the intestine. Its co-administration with statins reduced systemic LDL levels by more than 20%.^175–177^ Adding ezetimibe to statin treatment significantly decreases the risk of cardiovascular events and further reduces residual risk in patients already receiving maximally or maximally tolerated statin remedy and in patients with diabetes.^178–180^ Similarly, involving an inhibitor of protein convertase subtilisin/kexin type 9 (PCSK9) to statin treatment, which can lower plasma LDL levels, demonstrated good therapeutic effects.^181^ Compared to statin treatment alone, the combination strategy reduced LDL levels by over 50%.^182^ However, it should be noted that this therapy might bring the risk of residual inflammation to the patients.^183^

Antiplatelet-anticoagulation therapy is another AS-treatment strategy. Coagulation appears to be involved in AS primarily by activating protease-activated receptors. Dual antiplatelet therapy, including the traditional anticoagulant aspirin in combination with an ADP inhibitor or the platelet P2Y12 ADP blocker (prasugrel and ticagrelor), has been used for coronary artery disease patients.^184–186^ However, hemorrhage is the most severe adverse reaction of the modified strategy. To avoid counteracting the efficacy of combination therapy due to hemorrhage adverse events, several researchers combined the use of antiplatelet and anticoagulant, reducing thrombotic events, stabilizing plaque, inhibiting inflammations, and minimizing bleeding risk.^186^ With the in-depth understanding of AS pathogenesis, many new drugs have emerged in an endless stream. Several new combination strategies, i.e., the combinations of ezetimibe-lomitapide or -PCSK9 inhibitors, demonstrated promising potential against AS in patients not statin tolerant.^187,188^

Although lipid-lowering therapy and antithrombotic treatment are the primary treatment strategies for AS patients, the potential risk of cardiovascular inflammation affects the prognosis.^189^ The CANTOS trial found that adding anti-inflammatory therapy to the AS treatment displayed hopeful treatment outcomes.^190^ Moreover, a clinical phase III study indicated that interventions targeting NLRP3 inflammasome-IL-1β using canakinumab and colchicine could reduce the recurrence rate of cardiovascular events in patients with previous myocardial infarction, confirming the necessity of adding anti-inflammatory therapy to the AS treatment strategy.^191^

#### NP-mediated co-delivery

The most widely utilized co-delivery systems for AS treatment are high-density lipoprotein (HDL) /HDL mimicking NPs and liposomal NPs. They could be an efficient carrier for drug delivery and combat AS by reversing cholesterol transport and alleviating inflammatory and oxidation effects.^192,193^ (Fig. 7) The most extensively studied drug carriers are rHDL NPs, reconstituted from apolipoprotein A-I (apoA-I) and phospholipids.^194^ Furthermore, various functionally modified rHDL, such as HA-coated HDL-NPs and integrin-targeted NPs, was developed to treat AS.^195–197^ Recently, He et al. designed a β-cyclodextrins (β-CD)-anchored rHDL, in which β-CD was utilized to efflux intracellular cholesterol.^198–200^ First, the interaction between β-CD and drug-loaded discoidal-rHDL (d-rHDL) was investigated using the shuttle/sink model.^198^ They uncovered that β-CD could enhance macrophage-cholesterol outflow and move it to d-rHDL. Their combined use promoted intracellular drug delivery and inhibited intracellular-lipid deposition and inflammatory-cytokine release. Consequently, they anchored β-CD to simvastatin-loaded d-rHDL (ST-d-rHDL) for combined therapy. The results showed that either the combination of ST and d-rHDL or β-CD and ST-d-rHDL synergistically affected cholesterol removal and inflammation inhibition. The mechanism study indicated that β-CD-ST-d-rHDL inhibited the secretion of the inflammatory factor TNF-α through the mevalonate pathway and alleviating the inflammatory response.^200^ The findings show that d-rHDL can be used as a drug carrier and active agent acting collaboratively with other anti-AS drugs. Furthermore, they cross-linked the aforementioned β-CD-anchored rHDL, NP^3^_ST_, with HA-ferrocene (HA-Fc) conjugates through multivalent host-guest interactions and prepared the nanoassemblies HA-Fc/NP^3^_ST_.^199^ HA-Fc/NP^3^_ST_ responded to high levels of ROS at the lesion site and disassembled and penetrated deeper into the plaque. In vivo anti-AS study showed that HA-Fc/NP^3^_ST_ significantly inhibited plaque growth (the plaque size was half that of the saline group), reduced lipid deposition by 63%, and lowered systemic inflammation levels. Additionally, HDL could deliver small RNAs to endothelial cells.^201,202^ Wiese et al. utilized HDL to deliver locked-nucleic acid (LNA) miRNA inhibitors of miR-92a-3p and miR-489-3p to aortic endothelium in vivo.^203^ The results suggested that treatment with HDL alone affected 50% of AS-related genes and reduced the area of necrosis of lesions, whereas the dual LNA altered an entirely new set of genes, reducing AS lesion areas.

HDL enables cholesterol efflux through a cholesterol receptor or activating the macrophage liver X receptors (LXRs) to achieve targeted enhanced reverse cholesterol transport (RCT). However, systemic LXRs activation leads to excess lipogenesis accumulation in the liver and side effects, such as hepatic lipogenesis and hypertriglyceridemia.^204,205^ Guo et al. developed synthetic HDL (sHDL) derived from phospholipid-reconstituted apoA-I peptide (22a) to deliver LXRs agonists and promote cholesterol efflux by activating macrophage LXRs.^206^ The 12-nm sHDL allowed AS-plaque targeting and reduction of hepatic lipogenesis. After long-term treatment, the hepatic LXR expression was not increased in the sHDL group; however, the BCA1 mRNA expression was significantly increased in leukocytes—however, the mechanism of sHDL targeting AS the lesions was not explored.

Targeting the inflammatory cascade and polarization of macrophages in a pro-inflammatory direction can be a promising strategy against AS.^207^ Sheng et al. developed zeolitic imidazolate framework-8 (ZIF-8) NPs loaded with losartan potassium LP (LP@ZIF-8) for plaque-targeting using the EPR-like effect.^208^ ZIF-8 is a material that could facilitate autophagic activity in foam cells, stimulate RCT, and regulate lipid activity. ZIF-8 could disassemble due to the weak acid microenvironment (pH 5.5) in diseased aortic tissue releasing the encapsulated LP and downregulating ROS and the inflammatory factors (IL-1β, IL-6, TNFα). In AS-model mice, LP@ZIF-8 was synergistic in lipid clearance and anti-inflammation, significantly reducing the total plaque area and inflammatory damage. Recently, redox-responsive NPs for co-delivering simvastatin and ticagrelor were developed.^209^ The redox-responsive nanoprodrug of simvastatin (TPTS) was synthesized by conjugating α-tocopherol polyethylene glycol derivatives and statin pharmacophore hydroxylactone ring with thioketal. The second drug, ticagrelor, was encapsulated using the self-assembly property of TPTS. In the induced RAW264.7 inflammatory cell model, the codelivery system exerted a synergistic effect to inhibit polarization and reduce oxidative stress levels. In vivo studies indicated that modifying CREKA peptide allowed the NPs to target the plaque, ROS-stimulated releasing simvastatin, α-tocopherol and ticagrelor in atherosclerotic plaques, effectively inhibiting inflammation.^209^ Interestingly, He et al. reported a co-delivery system against inflammation at AS lesions by loading anti-miR155 onto baicalein nanorods and then layering with sialic acid (SA) for macrophage targeting.^17^ The 150-nm targeted nanorods efficiently delivered anti-miR155 to the cytosol, polarizing M1 to M2 and reducing the production of inflammatory factors. In vivo studies have shown that nanorods can target plaque and reduce blood pressure by more than 40% by increasing the diameter of the arterial lumen, inhibit the release of inflammatory factors (typically, TNF-α was reduced by nearly ten times after combined treatment), reduce lipids and promote M2 polarization, ultimately relieve AS. The system realized the co-delivery of biopharmaceutical and chemical drugs. The drug-loading capacity was as high as 80%, and the targeting of SA coating significantly improved the transfection efficiency. Furthermore, the co-delivery system entered the cell *via* caveolar endocytosis, reducing the endo-lysosome’s gene degradation. These advantages bode well for the future development of the co-delivery system.

Damage and inflammation of the plaque microenvironment donate plaque advancement.^210^ Li and He et al. developed pH-sensitive liposome loading with the anti-inflammatory oridonin and plaque-collagen protector (marimastat) for AS treatment.^211^ The results demonstrated that the liposome administration enabled effective anti-AS efficacy in high-fat diet-Apoe^−/−^ mice by reducing the pro-inflammatory cytokine secretion, shrinking the lesion region, and decreasing the plaque-collagen degradation.

## Pulmonary arterial hypertension (PAH)

PAH is a rather advanced disorder, portrayed by average pulmonary arterial pressure growth of >25-mm Hg under static conditions or >30-mm Hg in exercise.^212^ The PAH development leads to right ventricular hypertrophy, which, if not careful, results in heart failure and death. PAH occurs in patients with scleroderma, congenital heart disease, down syndrome, liver and lung disorders, HIV and COVID-19, and portal hypertension.^213–215^ There is no known cure for PAH, managed only by monotherapy centered on oxygen therapy, calcium channel blockers, prostaglandins, diuretics and vasodilators, and lifestyle modifications.^216^ Combinatorial PAH therapies have been recommended for patients with inadequate clinical responses to monotherapy.

### Pathways for PAH development

**Fig. 8:**
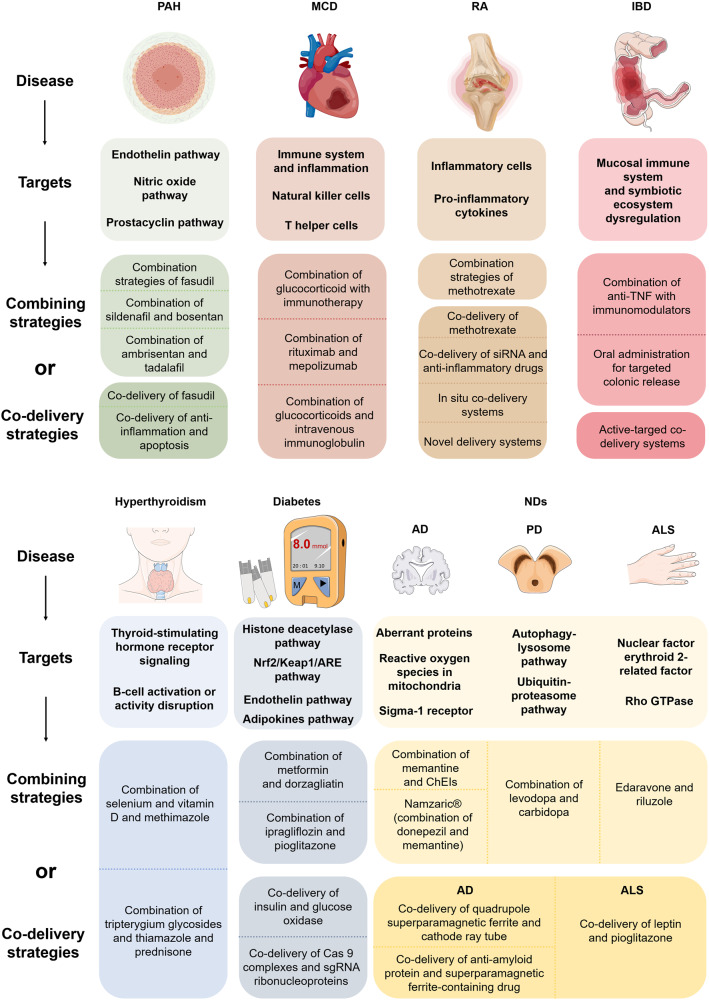
Targets and combining strategies for PAH, MCD, RA, IBD, hyperthyroidism, diabetes and NDs therapy. PAH, MCD, RA, and IBD are inflammation-associated diseases. For treating PAH and RA, fasudil- and MTX-based NP codelivery is the most frequently reported, respectively. For the MCD treatment, the combination of glucocorticoids and immunotherapy is often used. For IBD therapy, NP-codelivery is developed to target the inflammatory sites and increase drug availability and therapeutic efficacy, aiming to reduce the administration frequency and adverse side effects. For diabetes treatment, the typical case is the co-delivery of GLP-1 and DPP4 inhibitors. A combination of tripterygium glycosides and chemical compounds is promising to combat hyperthyroidism. For ND therapy, NP codelivery primarily aims to overcome the BBB barrier, i.e., mesoporous silica NPs for co-delivering leptin and pioglitazone. Parts of the figure were drawn using Servier Medical Art licensed under a Creative Commons Attribution 3.0 Unported License (https://creativecommons.org/licenses/by/3.0/)

Traditional PAH-associated therapies target three vasodilation-related signaling pathways: endothelin, nitric oxide (NO), and prostacyclin.^217^ Endothelin is a potent vasoconstrictor that stimulates vasoconstriction, proliferation, and fibrosis of smooth muscle cells. The endothelin receptor antagonists include ambrisentan and bosentan.^218^ Endothelin-1 (ET-1) works by two receptors, Endothelin-A and -B. Patients with PAH disorder have increased ET-1 levels typically found in the lungs and circulation, thus allowing ET-1 to be a promising treatment target.^219–221^ The NO pathway is targeted through phosphodiesterase-5 (PDE5) inhibitors, including tadalafil, sildenafil, riociguat and soluble guanylate cyclase (sGC) stimulator.^222^ Restoring cGMP levels is central to therapy in the NO-sGC-cyclic guanosine monophosphate (cGMP) axis. In health, NO triggers the vasodilator cyclic guanosine cGMP production and promotes vasodilation. In PAH disease conditions, however, patients typically have reduced circulating endogenous NO, facilitating the disease process.^223^ Since cGMP is rapidly degraded when PDE5 is expressed, blocking the action of PDE5 could potentially restore it to normal levels, dilating blood vessels and improving patient symptoms.^224^ Furthermore, because topical sGC activators are not limited by reducing endogenous NO levels, the NO pathway can be directly targeted with sGC stimulators to enhance cGMP activity.^225^ Prostacyclin analogs target the prostacyclin pathway using epoprostenol, iloprost, treprostinil and beraprost.^226,227^ PAH patients also have decreased prostacyclin synthase, with low prostacyclin produced in the pulmonary artery endothelial cells, decreasing cyclic adenosine monophosphate levels and leading to overproliferation and vasoconstriction of smooth muscle cells.^228,229^ Prostacyclin circulations by prostacyclin analogs induce vasodilation of pulmonary arterioles and constrain platelet aggregation and the proliferation of smooth muscle cells.^230,231^ Exogenous prostacyclin analogs supplemented with endogenous prostacyclin analogs are an effective treatment for PAH.^232–234^ (Fig. 8).

### Strategies for combinatorial PAH therapy

#### Combining therapy strategies

Compared to monotherapy, combining therapy is a more valued preference for managing patients with PAH as it can simultaneously target the instability of several critical biological routes in the pulmonary arteries and alleviate indications associated with PAH disorder.^235–237^ (Fig. 8) However, combining therapy only for traditional vasodilation often marginally increases the therapeutic effect in clinical trials and meta-analyses, and it is challenging to reduce mortality.^238–240^ The commonly used combined regimens are summarized in Table 3. COMPASS-2 is a PAH clinical test with a principal morbidity/mortality (M/M) termination, which combined sildenafil and bosentan for eight years.^241–243^ As crucial as this trial was, it could not reach its endpoint.^244^ The AMBITION trial tested the efficacy and safety of preliminary combinatorial treatment with ambrisentan and tadalafil. The treatment failure was reduced by 50% using the combination treatment.^245^

**Table 3:** Clinical research on combining and co-delivering strategies against PAH, MCD, RA, IBD, metabolic disorders and ND diseases

Disease	Combining or co-delivering drugs	Duration	Patient numbers	Efficacy	Study Phase	References	Additional information
**PAH**	Epoprostenol + Sildenafil vs. Epoprostenol + Placebo	2.6 years	Sildenafil (*n* = 134) Placebo (*n* = 131)	A placebo-adjusted increase of 28.8 meters (95% CI, 13.9 to 43.8 meters) in the 6-minute walk distance occurred in patients in the sildenafil group.	–	^511^	–
	Macitentan + Tadalafil + Selexipag vs. Macitentan + Tadalafil + Placebo	4 years	Selexipag (*n* = 123) Placebo (*n* = 122)	The risk for disease progression (to the end of the main observation period) is reduced with initial triple versus initial double therapy.	Phase 4	^512^	TRITON NCT02558231
	Sildenafil + Bosentan vs. Sildenafil + Placebo	7.2 years	Bosentan (*n* = 159) Placebo (*n* = 175)	17% risk reduction for time to first morbidity/mortality event. (*P* = 0.25)	Phase 4	^513^	COMPASS-2 NCT00303459
	3 or 10 mg Macitentan vs. placebo (63.7% receiving study drug combined with other therapy—PDE5, inhaled or oral Prostanoid)	3.8 years	Macitentan (*n* = 492) Placebo (*n* = 250)	10-mg macitentan dose reduced 45% the risk of M/M events. (*P* < 0.001)	Phase 3	^514^	SERAPHIN NCT00660179
	Selexipag (80% combining with ERA, PDE5, or both)	4.3 years	*n* = 1156	40% risk reduction of M/M event. (*P* < 0.0001)	Phase 3	^515^	GRIPHON NCT01106014
	Tadalafil + Ambrisentan vs. monotherapy with either agent	3.7 years	Tadalafil + Ambrisentan (*n* = 302) Ambrisentan (*n* = 152) Tadalafil (*n* = 151)	50% risk reduction of clinical failure. (*P* = 0.0002)	Phase 3	^516^	AMBITION NCT01178073
	Treprostinil + Beraprost vs. Treprostinil + Placebo	6.8 years	Beraprost (*n* = 137) Placebo (*n* = 136)	A reduced number of participants experienced clinical worsening.	Phase 3	–	NCT01908699
	Sildenafil + Sitaxsentan vs. Sildenafil + Placebo	2.3 years	Sitaxsentan (*n* = 91) Placebo (*n* = 92)	6MWD increased significantly at week 12. (*P* = 0.0104)	Phase 3	–	NCT00795639
	Sitaxsentan + Sildenafil vs. Sitaxsentan + Placebo	1.8 years	Sildenafil (n = 64) Placebo (n = 67)	PEP not met. 6MWD increased significantly at week 12. (*P* = 0.0049)	Phase 3	–	NCT00796666
	Treprostinil (50% combining with ERA, PDE5, or both)	4.2 years	Treprostinil (*n* = 174) Placebo (*n* = 176)	PEP not met. 6MWD increased at week 12.	Phase 3	^517^	FREEDOM-C NCT00325442
	1.5 mg or 2.5 mg Riociguat vs. Placebo (50% of participants pre-treated with an ERA or a Prostacyclin analog)	3.5 years	Riociguat (*n* = 317) Placebo (*n* = 126)	The change in 6MWD increased 36% with Riociguat, compared with the placebo, and both PVR and NT-proBNP levels decreased significantly. (*P* < 0.0001)	Phase 3	^518^	NCT00810693
	Epoprostenol + Sildenafil vs. Epoprostenol + Placebo	3 years	Sildenafil (*n* = 134) Placebo (*n* = 133)	6MWD improved or maintained in 59%, 44%, and 33% of patients at 1, 2, and 3 years, respectively.	Phase 3	^519^	OLE NCT00159861
**MCD**	Prednisone + Azathioprine vs. Prednisone + Placebo	0.5 year	Azathioprine (*n* = 43) Placebo (*n* = 42)	Compared with baseline, a combination of prednisone and azathioprine significantly improved left ventricular ejection fraction and decreased left-ventricular dimensions and volumes.	–	^274^	TIMIC
	Immunoglobulin + Ciclosporin vs Immunoglobulin	3 years	Immunoglobulin + Ciclosporin (*n* = 86) Immunoglobulin (*n* = 87)	The combination of immunoglobulin and ciclosporin reduced the incidence of coronary artery abnormalities. (*P* = 0.01)	Phase 3	^279^	KAICA CCT-B-2503
	Gamma globulin + Creatine phosphate+ Routine treatment vs. Routine treatment	0.5 years	Gamma globulin + Creatine phosphate + Routine treatment (*n* = 62) Routine treatment (*n* = 59)	The combination significantly increased the response rate (*P* < 0.05) and improved cardiac function. (*P* < 0.05),	–	^280^	–
**RA**	Methotrexate + MP-435 vs. Methotrexate + Placebo	1.8 years	MP-435 (*n* = 50) Placebo (*n* = 49)	The combination significantly increased the response rate of ACR 20, and decreased the incidence of serious adverse events.	Phase 2	–	NCT01143337
	Methotrexate + 300 mg, 150 mg, 75 mg, 25 mg Secukinumab vs. Methotrexate + Placebo	1.2 years	Secukinumab (*n* = 186) Placebo (*n* = 50)	PEP was not met. Symptom alleviation after long-term treatment with 150 mg of secukinumab.	Phase 2	^520,521^	NCT00928512
	Methotrexate + 20 mg, 40 mg Adalimumab vs. Methotrexate + Placebo	1 year	Adalimumab (*n* = 419) Placebo (*n* = 200)	(a) Meeting ACR20 Response Criteria: 63% and 61% Adalimumab, 30% Placebo. (*P* ≤ 0.001) (b) Achieving more comprehensive disease control	Phase 3	^522^	DE019 NCT00195702
	Methotrexate + Adalimumab vs. Methotrexate + Placebo	1.6 years	Adalimumab (*n* = 515) Placebo (*n* = 517)	Achieving the sLDA.	Phase 4	^523^	OPTIMA NCT00420927
	Adalimumab + Methotrexate vs. Adalimumab or Methotrexate	2 years	Adalimumab + Methotrexate (*n* = 268) Adalimumab (*n* = 274) Methotrexate (*n* = 257)	The combination significantly improved physical functioning and HRQOL in patients. (*P* < 0.0001)	Phase 3	^524^	PREMIER NCT00195663
	Methotrexate + Golimumab vs. Methotrexate + Placebo	48 weeks	Golimumab (*n* = 132) Placebo (*n* = 132)	The combination significantly improved the response of ACR 20 and DAS 28. (*P* < 0.001)	Phase 3	–	NCT01248780
	Methotrexate + 100, 150 mg Peficitinib vs. Methotrexate + Placebo	52 weeks	100 mg Peficitinib (*n* = 175) 150 mg Peficitinib (*n* = 174) Placebo (*n* = 170)	The combination significantly improved ACR 20 response. (*P* < 0.001)	Phase 3	^525^	NCT02305849
	Methotrexate + Baricitinib vs. Methotrexate + Placebo	52 weeks	Baricitinib (*n* = 488) Placebo (*n* = 489)	The combination significantly improved ACR 20 response and mTSS. (*P* < 0.001)	Phase 3	^526^	NCT01710358
	Methotrexate + Certolizumab Pegol vs. Methotrexate + Placebo	52 weeks	Certolizumab Pegol (*n* = 660) Placebo (*n* = 219)	The combination significantly achieved more patients with sREM and sLDA. (*P* < 0.001)	Phase 3	^527^	NCT01519791
**IBD**	Azathioprine + Infliximab vs. Azathioprine + Placebo	0.7 year	Infliximab (*n* = 169) Placebo (*n* = 170)	The combination s attained significantly higher rates of corticosteroid-free clinical remission and mucosal healing. (*P* < 0.001)	Phase 3	^528^	SONIC NCT00094458
	5-Aminosalicylic Acid + Budesonide vs. 5-Aminosalicylic Acid + Placebo	8 weeks	Budesonide (*n* = 255) Placebo (*n* = 255)	The combination s allowed higher clinical and endoscopic remission. (*P* = 0.049)	Phase 3	^529^	NCT01532648
**Hyper-thyroidism**	Atorvastatin + Methylprednisolone vs. Methylprednisolone	0.75 years	*n* = 500	The combination improved the outcome of Graves’ orbital disease in patients with moderate to severe active eye disease with hypercholesterolemia.	Phase 2	^530^	NCT03110848
	Methimazole + selenium + calcifediol vs. Methimazole	0.8 years	*n* = 30	The combination improved the early efficacy of hyperthyroidism.	-	^374^	EUDRACT2017-005050-11
	Rituximab + thioamide antithyroid drug (ATD)	2 years	*n* = 27	Rituximab can assist ATD treatment to relieve Graves’ hyperthyroidism in young people.	Phase 2	^531^	ISRCTN20381716
	Rituximab + antithyroid drug	2 years	*n* = 27	The combination improved remission of Graves’ hyperthyroidism in young patients.	Phase 2	^532^	ISRCTN20381716
	Mycophenolate + methylprednisolone vs. methylprednisolone	0.7 years	Mycophenolate *n* = 83 Methylprednisolone *n* = 81	The combination improved the remission rate of patients with active moderate-to-severe Graves’ orbitopathy.	–	^533^	MINGO EUDRACT2008-002123-93
**Diabetes**	Aspirin + Rivaroxaban vs. Aspirin + Placebo	3 years	No diabetes mellitus (*n* = 11356) Diabetes mellitus (*n* = 6922)	The combination showed especially advantageous in individuals with diabetes mellitus. (2.7% vs. 1.0%; *P* = 0.001)	Phase 3	^534^	NCT01776424
	Metformin + Vildagliptin vs. Metfromin + Placebo	5 years	Combination treatment group (*n* = 998) Metformin monotherapy group (*n* = 1003)	The combination decreased in the relative risk for time to initial treatment failure was seen in the early (hazard ratio 0:51; 95 percent confidence interval. (0:45–0:58; *p* = 0.0001)	Phase 4	^535^	NCT01528254
	Empagliflozin + Loop diuretics vs. Empagliflozin + Placebo	6 weeks	*n* = 23	The combination increased the 24 h urine volume without increasing urinary sodium.	Phase 4	^536^	NCT03226457
	Dorzagliatin + Metformin vs. Placebo + Metformin	4 years	*n* = 767	The combination produced efficient glycemic control with a good tolerance and safety profile in T2D patients. (*P* < 0.0001)	Phase 3	^409^	NCT03141073
**AD**	ChEIs + Memantine	4 years	*n* = 382	The combination decreased cognitive and functional degeneration.	–	^537^	–
	Rivastigmine + Memantine	0.5 year	*n* = 150	The combination maintained global and cognitive function and behavioral outcomes.	Phase 4	^538^	NCT00305903
	Masupirdine + Donepezil + Memantine vs. Placebo	0.5 year	Masupirdine (*n* = 375) Placebo (*n* = 189)	Concurrent administration of masupirdine adversely affected with memantine so necessary for further research on masupirdine.	Phase 2	^539^	NCT02580305
**PD**	Levodopa-carbidopa intestinal gel (LCIG)	1.2 year	*n* = 39	The combination reduced the number of non-motor symptoms and motor fluctuations in advanced PD patients.	Phase 3	^540^	NCT01736176
	Carbidopa (25 mg) + Levodopa (100 mg) + Entacapone (200 mg)	0.7 year	*n* = 493	The combination improved symptoms, without raising the risk of motor problems.	Phase 3	^541^	NCT00134966
	Carbidopa + Levodopa	3.5 months	*n* = 38	The combination offered preliminary evidence of efficacy, safe and feasible for PD.	Phase 2	^542^	NCT02577523
**ALS**	Celecoxib + Creatine + Minocycline	6 weeks	*n* = 86	The combination significantly improved protection against anterior horn motor neuron depletion.	Phase 2	^543^	NCT00919555
	Triumeq (dolutegravir 50 mg, abacavir 600 mg, lamivudine 300 mg)	5.5 months	*n* = 43	Transposable element activity can be a therapeutic target for human tauopathies.	Phase 2	^544^	NCT02868580

*PEP* primary endpoint, *ERA* endothelin receptor antagonist, *PDE5* phosphodiesterase-5 inhibitor, *6MWD* change from baseline in total distance walked during 6-minute walk distance, *PVR* pulmonary vascular resistance, *ACR20* American College of Rheumatology 20% response criteria, *ACR50* American College of Rheumatology 50% response criteria, *HRQOL* health-related quality of life, *mTSS* change from baseline in van der Heijde-modified total sharp score, *ACR 20 response* ≥20% improvement in RA symptoms and disease activity, *DAS 28 response* disease activity index score response, *sREM* sustained remission, *sLDA* sustained low disease activity

#### NP-mediated co-delivery

Fasudil is a Rho kinase inhibitor used to inhibit the effects of PAH involving Rho-kinase. In addition to effectively dilating pulmonary blood vessels, it can inhibit peripheral pulmonary artery-wall damage and restore the proliferation-apoptosis balance of pulmonary artery endothelial cells, smooth muscle cells, and fibroblasts.^246^ An investigation from Gupta’s group displayed that intratracheal administration of liposomal fasudil attenuated the mean pulmonary arterial pressure (mPAP) in a monocrotaline (MCT)-induced model, indicating its efficacy on PAH.^247^ Furthermore, Ahsan et al. probed the co-delivery efficacy of fasudil and DETA NONOate (a long-acting nitric oxide donor).^248^ Combination therapy significantly reduced mPAP and extended vasodilatory duration compared to monotherapy in acute and chronic PAH animal models. Also, improving right heart function could predict treatment outcomes with this therapy. Research indicated that surface CAR-modification of fasudil-DETA NONOate liposomes could significantly increase the accumulation of liposomes at the lesion site and drug release time. The studies in MCT- and SUGEN hypoxia-induced models indicated that the liposomal formulation was more profound in reducing several indicators, such as mPAP, medial arterial wall thickness, collagen deposition and muscularization degree over the free combination. CAR-modified liposomes were more selective in reducing mPAP than unmodified liposomes. Also, CAR-modified liposomes of a superoxide scavenger (superoxide dismutase (SOD) and fasudil, reduced a >50% mPAP and decelerated right ventricular hypertrophy compared with a single drug or a simple combination.^249^ Additionally, Huang et al. developed a fasudil-DCA prodrug that simultaneously allowed pulmonary vasodilation and inhibition of pyruvate dehydrogenase kinase to impede pulmonary artery remodeling and combat right heart dysfunction.^250^ This series of fasudil-related nanoparticle codelivery is attracting increasing attention, and combination therapy of vasodilators and right-heart function-improving drugs, such as co-delivery of fasudil and DETA NONOate, may represent a promising approach against PAH. Moreover, nanoparticle-mediated codelivery could elevate the treatment effect in several aspects due to the advantages, such as improved target ability and the multi-administration routes that can meet the particular needs of clinical patients.

Inflammation therapy against PAH has recently attracted increasing attention.^251^ A variety of potential pro-inflammatory cells, such as monocyte, macrophages and lymphocytes, is involved in pulmonary circulation. Pulmonary artery SMCs (PASMCs) can also directly secrete various pro-inflammatory factors (IL-1β, IL-6, P-selectin, etc.) to exacerbate pulmonary vascular remodeling and accelerate the process of PAH.^252–254^ He et al. developed a rod-like targeted co-delivery system of the apoptosis-executing gene p53 and the anti-inflammatory baicalein, assembling the nuclear localization signal peptide-p53 conjugate onto the rod-like baicalein nanocrystals and following by glucuronic acid-modification for PASMC-targeting.^255^ This rod-shaped nanoparticle is different from ordinary round particles and enters cells through caveolin, avoiding degradation by lysosomes. The results in vitro and in vivo showed that the co-delivery system could target the lung-PAs-PASMC axis and combat MCT-induced PAH by reducing mPAP, downregulating TNF-α, and impeding remodeling of the pulmonary artery and right ventricular. Furthermore, this study found that effective anti-inflammatory therapy was promising to combat pulmonary hypertension, activating the apoptotic executor signaling axis (p53-Bax-Bcl-2-caspase 3) and potentiated PASMC apoptosis. The downregulated fork-head box O1 (FoxO1) and caspase 3 intensify the proliferation of PASMCs and the PA remodeling.^256^ PTX is an often utilized chemotherapeutical drug; nevertheless, a study revealed that PTX could upregulate FoxO1 and inhibit PASMC proliferation.^256^ A recent report by the same group indicated that co-delivery of FoxO1 stimulus PTX and pro-apoptosis protein caspase 3 to PASMCs could attenuate MCT-induced PAH model, using the similar co-delivery technique that the active protein was loaded onto PTX nanocrystals.^256^

## Myocarditis (MCD)

MCD is an inflammatory disorder of the myocardium, usually caused by a viral infection, direct toxicity, or immune-mediated response to drugs, including immune checkpoint inhibitors and some systemic autoimmune diseases, followed by inflammatory permeation of the myocardium with degenerative and/or necrotic changes in adjacent cardiomyocytes.^257^ The MCD incidence in the population is unknown yet. According to the latest statistics, the incidence of adults is greater than 5%. Especially due to the impact of COVID-19, the data has suddenly increased.^258^ Accurate diagnosis of MCD is difficult because of its heterogeneity, and the clinical manifestations vary greatly.^259^ MCD is a significant cause of accidental death in young patients suffering from heart disease, especially in athletes. Chronic inflammatory dilated cardiomyopathy may develop in up to 20% of patients with MCD.^260,261^

### Targets for MCD therapy

Inflammation, a hallmark of MCD, is caused by various immune system cells during the disease process. It is known from the inflammatory responses in different MCD models that natural killer cells and CD4 and CD8 T cells are critical immune cells infiltrating the lesions in the early stage of MCD.^262–265^ Subsequent infiltration of neutrophils and macrophages accompanied by T cells significantly contributes to the MCD progress.^266–268^ Recently, the pathogenic role of Th17 cells in MCD has been gradually emphasized.^269,270^ All in all, targeting the immune system and anti-inflammatory is the most fundamental and effective MCD treatment (Fig. 8). Also, combined treatment with anti-inflammatory or immunotherapy according to the MCD pathogenesis can improve the treatment outcomes (Table 3).

### Strategies for combinatorial MCD therapy

#### Combining therapy strategies

Current MCD therapy mainly concentrates on combining glucocorticoids with immunotherapy (Fig. 8). Combining prednisone with immunosuppressants, such as cyclosporine (CA) or azathioprine (AZA), can effectively improve cardiac function.^271,272^ For instance, AZA treatment facilitated the increase of the left ventricular ejection fraction and the reduction of the New York Heart Association functional class.^273,274^ For patients who cannot tolerate AZA due to liver disturbance, methotrexate (MTX) is considered a replacement. E.g., the combination of MTX and prednisone was demonstrated to treat autoimmune virus-negative MCD effectively.^275^ These results confirmed the reliability of adding immunosuppressants to steroid drug therapy. For patients with glucocorticoid-resistant MCD, a combination of rituximab (RTX) and mepolizumab (MPZ) can be utilized.^271^ RTX fights against vasculitis by depleting B cells, and MPZ binds to IL-5 and prevents it from interacting with receptors on the surface of eosinophils. Combining RTX as induction therapy and MPZ as maintenance therapy could decrease steroid dose, prolong remission, and reduce relapse frequency.^271,276^

Intravenous immunoglobulin (IVIG) inhibits viral replication and activates the cellular and humoral immune responses, exhibiting dual immunosuppressive effects and potential in treating MCD. Of note, IVIG needs to be administered at high doses.^277^ The combination of glucocorticoids and IVIG accelerates the response process and reduces the incidence of organ failure.^278^ In addition, IVIG also could be combined with other drugs to treat MCD. Cyclosporine is a T-cell suppressor that restricts the transcription and release of crucial pathogenic pro-inflammatory cytokines through the calcineurin-NFAT pathway. In theory, cyclosporine prevents the inflammation progression in the arterial wall and stops the MCD development induced by Kawasaki disease. A phase III randomized controlled trial showed that patients tolerated IVIG in combination with cyclosporine, and this treatment strategy was more effective than conventional therapy using gamma globulin (IVIG) and high-dose aspirin.^279^ In a 2021 report, a combination of IVIG and phosphocreatine (CP) was administered to 121 young patients with MCD.^280^ CP is a fast-moving high-energy phosphate reserve and a cardioprotective agent. Clinical results disclosed that the modified combination therapy boosted the immune system of viral MCD patients.

#### NP-mediated co-delivery

Few NPs were reported to combinatorially combat MCD. Curcumin (Cur) is a polyphenolic flavonoid that can potentially prevent and treat various infectious, cardiovascular, and immune diseases. Increasingly evidence has shown that Cur could combat cardiovascular and inflammatory diseases.^281,282^ Remarkably, Cur rapidly reduced pathogen burden and mortality in mice following acute infection by reducing the expression of parasite-targeted low-density lipoprotein receptors during cell invasion.^283–285^ Recently, the scientist developed Cur-loaded PLGA-NPs in order to improve oral bioavailability. However, the authors did not offer the bioavailability data.^286^ The treatment study indicated that oral administration of Cur-loaded NPs in combination with a standard trypanosome drug benznidazole relieved chronic Chagas-induced MCD.^286^ The combined treatment decreased the pathogen burden at the source and modulated the course of infection in the body. The two drugs worked synergistically, improving treatment efficacy and tolerance in diseased mice *via* targeting cardiac hypertrophy, alleviating parasite burden and fibrosis and lowering the levels of cardiac biomarkers and inflammation-related substances.

## Rheumatoid arthritis (RA)

RA, an autoimmune disorder, is stamped by inflammation and matrix destruction of the bone and cartilage.^287^ The exact mechanism causing RA remains unclear; however, imbalances in the body’s immune system are generally considered an essential factor in RA occurrence.

### Targets for RA therapy

The inflamed joints in RA contain numerous misactivated immune cells, such as T cells, B cells, neutrophils, macrophages, and dendritic cells, and they could release pro-inflammatory factors, including IL-1β, TNF-α, and IL-6.^288,289^ These cytokines overflow into the bloodstream, causing systemic inflammation, while they induce local joint injury by boosting MMP production and activating osteoclasts.^290^ Meanwhile, various signaling pathways, such as Janus kinase–signal transducer and activator of transcription, Th17, IL-17/IL-17R, NF-κB, mitogen and activated protein kinases, are triggered *via* the excessive production of cytokines.^291,292^

Conventional drugs, such as glucocorticoids, non-steroidal anti-inflammatory drugs, disease-modifying anti-rheumatic compounds and biopharmaceuticals (TNF-α blockers), benefit RA treatment. Nevertheless, these medicines always have severe side effects, such as gastrointestinal bleeding, renal dysfunction and CVD risk.^293,294^ Moreover, frequent administration with high doses is required because of traditional drug therapy’s short biological half-life and poor bioavailability.^295^ Therefore, various new therapeutic regimens were established to overcome the limitations of conventional treatment.^296^ (Fig. 8).

### Strategies for combinatorial RA therapy

#### Combining therapy strategies

MTX is a commonly used anti-rheumatic immunosuppressant for RA treatment.^297^ Numerous MTX-based combination strategies were reported, such as combining MTX with hydroxychloroquine, sulfasalazine or steroids. MTX was also integrated with biological therapies against RA.^297,298^ Other drug combinations used in the clinical for RA therapy are summarized in Table 3.

#### NP-mediated co-delivery

Despite the high efficiency in the clinical use of biological therapies for RA, nearly 30% of patients still show low responsiveness due to heterogeneity. Furthermore, these therapies are costly and have a high risk of serious bacterial infections.^299–303^ Therefore, the recently combined therapeutics focus on improving the efficacy of available therapy by targeting inflamed joints.^304^ (Fig. 8) Nanocarriers could deliver the therapeutic agents to the particular inflammation site through loose vasculature in infected areas of RA, elevating the anti-inflammatory activity of medications while avoiding the administration at high doses and non-target effects.^293,305^ Nanocarriers, including NPs, hydrogel, micelles, and liposomes, are often utilized for the combinatorial delivery of two therapeutic agents, targeting the same cellular pathway, elevating the delivery efficiency, and decreasing side effects (Fig. 8).^304^ For instance, MTX and minocycline (MNC)-loaded PLGA NPs (MMNPs, 125 nm) were developed against RA.^306^ MMNPs had a 100–200 nm diameter and can accumulate in the RA lesions after intravenous injection. MMNPs demonstrated superior cytotoxicity to inflammatory RAW 264.7 cells at specific concentrations, a higher antibacterial effect than free MTX or MNC, and a 3-fold inhibition zone compared to free drugs. In another study, researchers developed the multifunctional hexagonal palladium-Cys@MTX@ arginine-glycine aspartic acid (RGD) peptides nanosheets for targeting inflammatory cells and controlling MTX release. The nanosheets could control MTX release using irradiation of 808 nm and significantly reduce MTX toxicity. In vivo data indicated that the combined strategy effectively inhibited RA symptoms by reducing the expression of pro-inflammatory cytokines.^307^ Also, targeted co-delivery of MTX and nimesulide using RGD-modified polymeric micelles to angiogenesis at low doses allowed enhanced anti-RA efficacy in the rat model.^308^ In addition, the long-lasting release of MTX and Dex using intra-articular injectable combined depot formulation of MTX-HA/Dex-microencapsulates demonstrated a synergistic effect on repairing RA joints and inhibiting inflammation by allowing the two drugs to work in the articular joint.^309^ Other combinatorial strategies, such as microwave hyperthermia plus thermosensitive liposome-loaded sinomenine hydrochloride (SIN)^310^ and sialic acid-modified dexamethasone palmitate-liposome-anchored neutrophils,^279^ were also reported for anti-RA treatment. MTX-based nanoparticle codelivery is the most commonly reported combination therapy, demonstrating the effectiveness of MTX. However, the molecular mechanism of these synergistic effects is still unclear. Further mechanism study may benefit their translation.

Gene therapy combing with anti-inflammatory effects has shown high potency in RA treatment.^299,303^ Park et al. demonstrated that the co-delivery of COX-2 siRNA and anti-inflammatory dexamethasone (Dex) showed promising therapeutic efficacy against RA.^311^ The co-delivery markedly downregulated the apoptosis-related and inflammatory factors, for example, caspase 3 and TNF-α in C28/I2 cells, compared to mono-treatment with Dex. PEGylated hybrid-NPs system encompassed calcium phosphate/liposomes co-loaded with NF-κB specific siRNA and MTX were developed to target macrophages, aiming to inhibit p65 and its translocation.^312^ In-vivo results demonstrated that the liposomal formulation could retard the RA progression by preventing the release of pro-inflammatory cytokines from macrophages without affecting the lymphocyte count, which could prevent the adverse effect of MTX. Another co-delivery system of siRNA and Dex using hybrid polymer micelles consisting of polycaprolactone-polyethyleneimine and polycaprolactone-polyethyleneglycol has also shown potential for inhibiting NF-κB signaling pathway in macrophages and polarizing macrophages from M1 to M2 in the arthritic synovium.^313^ Furthermore, folate acid-modified MTX-conjugated polymer hybrid micelles complexed with miR-124 *via* electrostatic interaction that targeted the activated macrophages in RA joints achieved the synergistic anti-RA effect in a rat adjuvant-induced arthritis model.^314^

The in-situ DDSs has promising application potential in treating RA due to its convenient administration, low frequency and high patient compliance. Kang et al. found that the transdermal delivery of nanostructured lipid carriers encapsulating celastrol and indomethacin (Cel-Indo-NLCs)-gel was effective in inhibiting pro-inflammatory cytokines compared to mono nano gel Cel-NLCs-gel or indo-NLCs-gel in RA rats.^6^ However, the efficacy and safety of Cel-Indo-NLCs to alleviate RA have not been thoroughly investigated. An in-situ hydrogel loaded with PEI-SS-IND-MTX-MMP-9 siRNA NPs (D/siRNA-NGel) was used to simultaneously deliver three drugs (indomethacin (IND), MTX, and MMP-9 siRNA) for treating RA by targeting multiple signaling pathways.^315^ The MMP-9 siRNA inhibited MMP-9 expression and the cartilage degeneration mediated by RA synovial fibroblasts; at the same time, the anti-inflammatory drug IND relieved patients’ pain, coupled with the fundamental anti-rheumatic effect of MTX.

## Inflammatory bowel disease (IBD)

IBD, defined as the chronic inflammation of the digestive tract, is clinically classified into Crohn’s disease (CD) and ulcerative colitis (UC).^316,317^ UC conditions cause long-lasting inflammations and ulcers in the innermost lining of the large intestine (colon) and rectum. In contrast, the CD is stamped by the lining inflammation of the entire gastrointestinal tract, resulting in granuloma formulation due to the plasma cell- and macrophage-clustering.

### Targets for IBD therapy

Although the two types of lesions differ, IBD is generally a recurrent inflammatory disease due to dysregulation of the mucosal immune system and symbiotic ecosystem.^318^ Due to its life-threatening, extensive research has been conducted to determine this disease’s environmental and genetic origins.^319–321^ The hyper-permeability of extravascular compartments and beds is the most crucial feature in IBD development. Intraluminal antigens or microbiota stimulates can deeply infiltrate the epithelium of immune cells and extensively migrate across the vascular endothelium. Antigen-presenting cells ingest these pathogenic factors and are activated, producing pro-inflammatory cytokines and chemokines.^322–324^ The inflammatory cycle at the lesion site persists due to the interaction between the inflammatory cells and pro-inflammatory factors. When macrophages, neutrophils, and dendritic cells accumulate within inflamed portions of the intestine, there is an increase in intestinal permeability to macromolecules, molecules, and cells.^325^ These focal microenvironments, favorable for drug penetration and aggregation, lay the foundation for drug design against IBD (Fig. 8).

### Strategies for combinatorial IBD therapy

#### Combining therapy strategies

Many IBD therapies target macrophages and cytokine by inducing polarization of alternatively activated macrophages or inhibiting inflammatory signaling pathways.^326^ (Table 3) The typical therapy regimen is the use of anti-inflammatory agents such as corticosteroids (Dex, hydrocortisone, prednisone), immunosuppressive agents (azathioprine, 6-mercaptopurine), and vascular adhesion molecules.^16^ In addition, three biologic drugs are approved for clinical use, including TNF-α antagonists, interleukin 12/23 antagonists, and integrins.^327^

IBD treatment is no longer restricted to temporary symptom alleviation but instead focuses on long-term strategies for deep remission.^328,329^ Therefore, the combination therapy of those mentioned above clinically effective therapeutic drugs has been intensively studied to enhance the effect of the drug and/or improve the pharmacokinetics, avoiding high-dose intravenous injection of drugs that may cause diarrhea, osteoporosis and other adverse reactions (Table 3).^330–332^ One of the most studied tactics is combining anti-TNF therapeutics with immunomodulators. This strategy can reduce immunogenicity and attain synergistic effects by regulating different inflammatory pathways and affecting pharmacokinetic parameters.^333–335^ Due to their anti-inflammatory effects, regulatory macrophages are essential to wound healing and gut homeostasis. Vos et al. reported that infliximab/azathioprine combination therapy accelerated the mucosal healing process up to twofold compared with infliximab treatment. In addition, the drug combination increased the number of regulatory macrophages and modulated the macrophage phenotype to enhance immunosuppression, providing theoretical support for clinical use.^336^ In another study, Colombel et al. found that the combination of azathioprine/infliximab elevated the anti-CD efficacy, likely due to the increased plasma concentrations of infliximab rather than a therapeutic synergistic effect of the two drugs. They argued that, if this theory is established, the treatment may need to maintain sufficient concentrations of the biologics, not requiring combination therapy and avoiding possible adverse reactions caused by azathioprine. Nevertheless, the biological drug consistently demonstrates poor stability and a short half-life, requiring frequent administration or pump implantation in patients and probably bringing potential limitations, such as poor compliance and infection. Until effective DDSs are developed for biopharmaceutical delivery, drug combinations may remain the most important treatment option.^337^

#### NP-mediated co-delivery

The combination of IBD therapy strategies always fails to deliver drugs to specific sites of inflammation, leading to frequent dosing and adverse side effects that may affect patient response to subsequent treatments.^18^ Hence, effective co-delivery systems are desired to target specific inflammatory sites for the pathological features of IBD and improve drug availability and therapeutic efficacy. The co-delivery preparations are usually administered orally for colon-targeted release. Alternatively, by intravenous injection, the NPs can passively or actively target the endothelium at IBD lesions with discontinuity and high permeability.^338^ E.g., Xiao et al. loaded TNFα siRNA (siTNF) into galactosylated polymer and prepared 260-nm GalsiTNF-NPs. Then, they co-loaded GalsiTNF-NPs and IL-22 in a chitosan/alginate hydrogel, protecting the drug in the digestive tract and releasing it in the colonic lumen.^339^ After oral administration, GalsiTNF-NPs targeted macrophages and repressed the TNFα expression, while IL-22 downregulated the pro-inflammatory factors and promoted mucosal healing in a UC model. Aib et al. co-encapsulated anti-inflammatory and antioxidant drugs, mesalazine and Cur, in liposomes and coated them with Eudragit-S100, conferring the liposomes colon-targeting release.^340^ The coated liposomes remained almost intact at pH 1.2 and rapidly released at pH 7.4, enabling drug delivery to the colonic site. In the UC Colitis model, the coated liposomes can effectively reduce various inflammatory markers for synergy therapy, dropping the level of oxidative stress and protecting the intestinal mucosa. Similarly, using the Eudragit-S100 coating for colon-specific delivery, Desai et al. developed colon-directed bioadhesive beads encapsulating Cur and cyclosporine.^341^ After reaching the colon site, the coating dissolved and allowed 100% colon adhesion of the pellets inside, reducing the administration dose and decreasing side effects.

Active targeting is a significant development direction for intravenous DDSs to treat IBD.^342^ Xu et al. reported a TKPR polypeptide-functionalized reversible cross-linking polymer (TKPR-RCP).^343^ They designed an asymmetric triblock copolymer to self-assemble and form a polymersome with a hydrophilic core inside, a macrophage-targeting polypeptide TKPR attached to the outside, and a redox-sensitive disulfide bond structure. Dexamethasone sodium phosphate and siTNF-α were co-encapsulated in the hydrophilic core of TKPR-RCP. The surface charge of the system is neutral, permitting blood safety and systemic circulation stability. Upon accumulating in the inflamed colons of the UC model, TKPR-RCP targeted macrophages and suffered redox-responsive membrane de-crosslinking, accelerating the intracellular drug release. The efficacy study indicated that TKPR-RCP/siTNF-α/DSP could knock down 80% TNF-α, almost a 2-fold reduction compared to control groups. Meanwhile, the preparation can inhibit the cascade reaction activated by inflammatory factors (IL-1β and IL-6) and prevent the infiltration of leukocytes, alleviating inflammation induced by several pathways. Also, Yan et al. designed a P-selectin-binding peptide (PBP) surface-modified 164-nm PLGA-NPs for co-delivering resveratrol (Res) and dietary triterpenoid betulinic acid (BA), synergistically achieving anti-inflammatory and antioxidant effects.^146^ PBP-PLGA-NPs could efficiently target Colon-26 and RAW 264.7 in vitro and accumulate in the inflamed colon. Moreover, intravenous injection of the NPs could relieve UC symptoms while maintaining intestinal microbiota homeostasis and not inducing organ injuries.

## Hyperthyroidism

The metabolic disorder known as hyperthyroidism is linked to excessive thyroid hormone production. The thyroid gland is a bilobed organ in front of the trachea, between the suprasternal notch and the cricoid cartilage. Secretion of thyroxine (T4) occurs in the thyroid gland as a reaction to thyroid-stimulating hormone (TSH) produced by the pituitary gland. Deiodinase enzymes transform the released T4 into the more powerful triiodothyronine (T3). Despite the thyroid gland’s inherent ability to produce T3, most of the conversion of T4 to T3 occurs outside of it. The thyroid gland’s follicular cells are spherical and polarized, and they surround a gel-like colloid rich in thyroglobulin. The organic precursor to thyroid hormones, thyroglobulin, needs iodide to become thyroid hormone.^344^ After being converted to iodide by the thyroid peroxidase enzyme, dietary iodine is carried into thyroid follicular cells through the sodium-iodide symporter. High dietary iodide levels temporarily suppress the organification process, whereas low dietary iodide facilitates upregulation of the sodium-iodide symporter. This process is termed the Wolff-Chaik off effect.^345^

The excessive secretion and production of these thyroid hormones then lead to hyperthyroidism. Moreover, there is a widespread misperception about the terms thyrotoxicosis and hyperthyroidism, which are used interchangeably. Excessive thyroid hormone exposure to tissues is called thyrotoxicosis, whereas hyperthyroidism is a disorder related to excessive thyroid hormone production. Even though the terms hyperthyroidism and thyrotoxicosis are sometimes used interchangeably, it’s crucial to understand the differences.

There are several forms of hyperthyroidism based on their causes or sources. Graves’ disease is the most prevalent cause of hyperthyroidism. This hyperthyroidism typically affects younger populations since Graves’ disease has an autoimmune etiology.^346^ Another cause of hyperthyroidism is toxic multinodular goiter. Toxic multinodular goiter is the most typical cause of hyperthyroidism in the older population. Even though toxic multinodular goiter and Graves’ disease are the leading causes of hyperthyroidism, there are other causes, such as iodine-induced hyperthyroidism (Jod-Basedow phenomenon), factitious thyroiditis, de Quervain thyroiditis (subacute thyroiditis), postpartum thyroiditis, and thyroid adenomas. For instance, factitious thyroiditis is caused by the excessive or improper use of pharmaceutical thyroid hormones. Thyroxine has the potential to be abused due to a well-liked side effect of reducing weight, so every history of a hyperthyroid patient should include a prescription list and an evaluation of potential abuse (whether intentional or unintentional). Similarly, drugs containing iodine or amiodarone can induce the Jod-Basedow phenomenon and iodine-associated hyperthyroidism or thyrotoxicosis.^347^

### Targets for hyperthyroidism therapy

#### Thyroid-stimulating hormone receptor (TSHR) signaling

Since stimulation of the TSHR is the primary cause of hyperthyroidism, various research teams have been working on methods to block TSHR signaling, either by employing small chemicals or antibodies which prevent receptor activation. Additionally, it is being explored if TSHR peptides have potential long-lasting immunomodulatory characteristics.^348^ One major benefit of this approach is that it is more focused and targeted and, theoretically, would not negatively affect the participant’s capacity to combat infection.

#### B-cell activation or activity disruption

Effective antigen presentation is primarily coordinated by CD40, a TNF family receptor located on thyrocytes and antigen-presenting cells, including B cells.^349^ When there is inflammation, its ligand CD154 (also known as CD40 ligand; CD40L) is momentarily produced on activated T cells and other nonimmune cells. A co-stimulatory pathway is activated by the CD40-CD154 interaction, offering the second signal for activating an adaptive humoral immune response.^350^ Given that the interaction between B and T lymphocytes depends on the formation of the intrathyroidal germinal center and the maturation of the B-cell repertoire for the production of thyroid-stimulating antibodies, it is hypothesized that this interaction is essential in the pathogenesis of hyperthyroidism.^350,351^

Several autoimmune diseases, such as hyperthyroidism, have been linked to CD40 gene variants that can alter thyroid antibody production and act as a relapse signal.^352–354^ Functional investigations have shown that the disease-associated CD40 mutation modifies the consensus Kozak initiation sequence, increasing translational efficiency and pointing to a causal relationship between overexpression of CD40 and the propensity for Graves’ hyperthyroidism.^355^ Indeed, evidence from a variety of murine models has disclosed that genetic or chemical manipulation of CD40 signaling can alter the severity of autoimmune thyroiditis or the generation of thyroid autoantibodies, designating CD40 as a promising target in the management of this condition.^351,352^

The neonatal immunoglobulin Fc receptor (FcRn), which binds to endocytosed Immunoglobin G (IgG) antibody in the lysosome’s acidic environment and recycles it to the cell membrane for release back into circulation, is responsible for IgG antibodies’ prolonged half-lives, including those of TRAbs.^356^ Various animal models of autoimmune disease have been augmented by blocking FcRn; and FcRn-deficient mice have demonstrated resilience to autoimmune disease.^357,358^ For IgG-mediated autoimmune disorders like Graves’ hyperthyroidism, accelerating antibody degradation and reducing circulating pathogenic TRAb and FcRn inhibition may represent an intriguing targeted therapy.^359^

B-cell activating factor (BAFF), a cytokine that belongs to the TNF family, is crucial for the activation, differentiation, and survival of B-lymphocytes. Patients with autoimmune diseases, such as active Graves’ hyperthyroidism, have elevated circulating BAFF levels, correlating with increased thyroid hormone and TRAb.^360^ Additionally, hyperthyroidism is linked to genetic variations of BAFF.^361,362^ As a result, BAFF could be a therapeutic target for autoimmune diseases driven by B cells.

### Strategies for combinatorial hyperthyroidism therapy

Over the years, hyperthyroidism has been treated in two means, depending on its underlying cause, including symptomatic and definitive treatments.^363^ For example, a beta-adrenergic antagonist like atenolol can manage the symptoms of hyperthyroidism, such as anxiety, palpitations and tremor. Also, patients who cannot tolerate beta-blockers or who have contraindications to beta-blocker therapy can be treated with calcium channel blockers, such as verapamil.^364^ Three conventional or definitive treatments are commonly used for the clinic: thionamide therapy, radioactive iodine therapy, and partial thyroidectomy. However, various limitations were reported with these therapies, such as high recurrence rate following drug use discontinuation, hypothyroidism, hepatitis, vasculitis, agranulocytosis and drug-induced lupus.^365–367^ Combinatorial treatment is promising to overcome the drawbacks (Fig. 8).

Graves’ disease patients have lower levels of serum selenium (Se) and vitamin D (VitD).^368–371^ Se could help thyrocyte defense against ROS that is upregulated in hyperthyroidism patients as integrated into selenoproteins (such as glutathione peroxidase).^368,370^ VitD influences the maturation and differentiation of immune cells, such as macrophages, dendritic cells, natural killer cells and T cell subsets, and switches them into tolerogenic and anti-inflammatory phenotypes.^372,373^ Consequently, Gallo et al. studied whether the combination use of Se and cholecalciferol (VitD) with the antithyroid drug methimazole enabled a faster control of hyperthyroidism in a clinical study (EudraCT 2017-00505011).^374^ Individuals with newly-onset Graves’ disease who had marginal or low Se and VitD levels were randomly treated with either MMI monotherapy or MMI in combination with Se and VitD. Se therapy was stopped after 180 days, while the others were continued. The combination therapy significantly reduced the serum-free thyroxine (FT4) levels compared to MMI monotherapy. Also, the composite score exhibited significant recovery in the intervention group compared to the MMI group, evidenced by the investigation of the life quality using a questionnaire for “Thyroid-related Patient-Reported Outcome.” Thus, the combinational treatment could raise the Se and VitD levels and boost the effectiveness of MMI treatments.

Another study by Xie et al. investigated the anti-hyperthyroidism efficacy and safety of combining tripterygium glycosides with thiamazole or prednisone.^375^ The data indicated that involving tripterygium glycosides decreased the exophthalmos, serum-free triiodothyronine, FT4, plasma osteocalcin, and alkaline phosphatase while increasing TSH, SOD, and glutathione peroxidase. Their findings demonstrated that combining tripterygium glycosides and chemical compounds is an efficient treatment against hyperthyroidism.

## Diabetes

Diabetes is a widespread metabolic disorder affecting a large population worldwide.^376^ Insulin is a hormone that regulates blood glucose in the body.^377,378^ Diabetes is a severe condition induced by either insufficient insulin secretion by the pancreas or inefficient insulin utilization by the body.^378^ The blood glucose level is highly increased in diabetes patients, occurring when pancreatic beta cells in the islets of Langerhans cannot produce adequate insulin. Treatment is selected according to the diabetes classification listed as follows.^379,380^ (1) Type 1 diabetes mellitus (T1DM): caused by autoimmune destruction of beta cells, typically resulting in total insulin deficiency.^381^ (2) Type 2 diabetes mellitus (T2DM) is caused by a progressive loss of insulin secretion from beta cells, frequently appearing in conjunction with insulin resistance.^382^ (3) Gestational diabetes mellitus is diabetes occurring in pregnant women.^383,384^ (4) Particular types of diabetes are caused by various factors, such as exocrine pancreatic diseases (cystic fibrosis and pancreatitis), monogenic diabetes syndromes (neonatal diabetes and maturity-onset diabetes of the young), and drug-/chemical-induced diabetes (glucocorticoid use, the compound treatment against HIV/AIDS, and organ transplantation).^385,386^

### Targets for diabetes therapy

#### Histone deacetylase pathway

The therapies can also target the intermediate substrate and glucose metabolism processes.^387^ Diabetes is alleviated by restoring insulin release from pancreatic β cells, with the rare exceptions of aberration in the insulin signaling cascade. As a result, maintaining β cell mass may be a promising strategy for treating diabetes.^388^ HDACs, such as sirtuins, are able to regulate the development of the pancreatic endocrine system, β-cell activities, insulin secretion, and metabolic fates.^387,389–391^ The HDAC-associated pathways are considered novel therapeutic targets in the management of diabetes.

#### The Nrf2/Keap1/ARE pathway

The main defense mechanism against oxidative and electrophilic stressors involves the Kelch-like ECH-associated protein 1/nuclear factor erythroid 2-related factor 2 pathway. Keap1, a component of an E3 ubiquitin ligase, precisely controls the transcription factor Nrf2 under homeostatic conditions by ubiquitination and proteasome-dependent destruction.^392^ This pathway has been extensively studied in cancer, chronic obstructive pulmonary disease, neurological diseases and autoimmune diseases, e.g., IBD and RA.^393–395^ However, the role of the antioxidant Nrf2/Keap1/ARE pathway in diabetic dysfunction was recently discovered, likely contributing to diabetes amputation.^396^

Lessening Nrf2-mediated ROS damage could be an approach against diabetes.^397^ Current pharmacological activators boost Nrf2 expression through three primary methods.^396^ The Nrf2 activators include as follows: (a) activating upstream kinases, such as protein kinase B and extracellular signal-regulated kinases, which phosphorylate specific sites facilitating Nrf2 release from Keap1; (b) altering Keap1 cysteine residues, which disassembles the Nrf2-Keap1 complex and promotes Nrf2 dissociation; and (c) preventing the ubiquitination of these pathways that enhances Nrf2 stability, nuclear translocation and antioxidant cascade.^396^ Notably, the Nrf2 activator, dimethyl fumarate (BG-12, brand name Tecfidera®), was approved in 2013 for treating multiple sclerosis. This compound enhances Nrf2’s downstream pathways and improves cytoprotective, anti-inflammatory and antioxidant effects. As a result, the Nrf2 pathway may be a treatment target for type 2 diabetes, whose conditions are closely related to oxidative stress. Several natural antioxidants, e.g., vitamin E, C, and coenzyme Q10, were explored to combat diseases.^398^ However, the results from clinical trials indicated that adjunct medicines showed modest efficacy in preventing or treating diabetes.^399^ A high throughput cell-based screening assay is now used to screen small-molecular activators for the Nrf2/Keap1/ARE pathway.^400^ New antioxidants would be found to alleviate oxidative stress and inflammation in type 2 diabetes.

#### Endothelin and adipokine pathways

The endothelium can modulate human homeostasis by controlling arterial blood pressure, delivering nutrients and hormones and providing a smooth surface that controls coagulation, fibrinolysis and inflammation.^401^ Endothelial dysfunction is a factor in the onset and progression of microvascular disease in diabetes, as well as most of the microvascular consequences, i.e., diabetic retinopathy, nephropathy and neuropathy.^402^ The key pathogenesis-related variables inducing endothelial dysfunction include hyperglycemia, insulin resistance, hyperinsulinemia and dyslipidemia.

Adipokines are a body’s biologics that regulate various physiological functions, including insulin sensitization, appetite regulation, inflammatory response, vascular homeostasis and energy balance.^403,404^ Adipokines involve anti-/pro-inflammatory/cytokines, adiponectin, fatty acid binding protein, etc. A clinical study discovered that several antidiabetic drugs, including glimepiride could elevate plasma adiponectin, peroxisome proliferator-activated receptor-alpha agonists like thiazolidinediones, renin-angiotensin system-blocking compounds like losartan, and triglyceride-lowering drug, such as simvastatin.^404^

### Strategies for combinatorial diabetes therapy

Clinically, T1DM is mainly treated with insulin replacement therapy.^405,406^ T2DM is the predominent cause of diabetes, with an incidence rate as high as 90–95%.^407^ Primary drug therapy includes insulin secretion inhibitors, biguanides, insulin sensitizers, alpha-glucosidase inhibitors, incretin mimetics, glucagon-like peptide-1 (GLP-1) and sodium-glucose co-transporter-2 (SGLT2) inhibitors.^382^ For patients who fail to achieve treatment goals with first-line oral antidiabetic drugs, combination therapy is often recommended. For gestational diabetes mellitus, 80%-90% of patients are recommended to use lifestyle therapy for blood glucose management (diet, physical activity, etc.).^383^ The causes of specific-type diabetes are always different. Targeted treatment is always encouraged according to the etiology, aiming to normalize the blood sugar level.

#### Combining therapy strategies

For most patients, modifying lifestyle and diet is also the leading choice for T2DM.^408^ Metformin is always selected as blood glucose levels cannot be controlled through diet and exercise.^409^ The effectiveness and safety of dorzagliatin as a supplement to metformin were assessed in T2DM patients with inadequate glycemic control using metformin alone.^409^ Metformin reduces plasma glucose levels and hepatic glucose synthesis,^410,411^ while dorzagliatin is an orally accessible glucokinase activator and reduces postprandial glucose by targeting the pancreatic and liver glucokinase.^412,413^ The results indicated that the combination allowed efficient glycemic control with good tolerance and safety, not causing severe hypoglycemia and other side effects (Fig. 8).

SGLT2 balances sodium-glucose transport proteins in the nephron, preventing the kidneys from glucose reabsorption and lowering blood sugar. SGLT2 inhibitors suppress the proximal nephron’s SGLT2 protein,^414^ reducing the glucose reabsorption in T2DM and increasing urinary glucose excretion.^415^ Dosing SGLT2 inhibitors could reduce weight, decline systolic blood pressure and lower glycemic level.^416^ Tahara et al. evaluated the treatment efficacy of the combination of SGLT2-selective inhibitor ipragliflozin (10 mg/kg) and pioglitazone (1 mg/kg) on nonalcoholic steatohepatitis in T2DM KK/Ay mice fed a high-fat diet.^417^ The results showed that the combination allowed significant reductions in hyperlipidemia, hepatic steatosis and fibrosis and improved obesity, insulin resistance and hyperglycemia.^417^

#### NP-mediated co-delivery

Various NPs were reported for delivering therapeutic compounds, including insulin, dipeptidyl peptidase-4 (DPP4) inhibitors, and plasmids containing the GLP-1 gene.^418^ To relieve the enzymatic breakdown of certain antidiabetic drugs like insulin in the gastrointestinal (GI) tract, the scientists designed several NPs, including mesoporous silica NPs (MSNs), liposomes, gold NPs and polymer NPs. However, drug codelivery systems may be exploited to simplify treatment regimens and improve patient compliance. Besides, NPs could be leveraged to co-deliver anti-diabetic gene therapeutics and peptides. Despite the potential advantages, few preclinical studies investigating NP-mediated antidiabetic combinations have been reported.

An MSN-based H_2_O_2_-responsive system was developed for dual stimuli-responsive (glucose and H_2_O_2_) insulin delivery.^419^ 4-(imidazoyl carbamate) phenylboronic acid pinacol ester and cyclodextrin (CD) were added to MSNs, enabling drug release in response to H_2_O_2_. The insulin and glucose oxidase were encapsulated in MSNs after surface modification. The release study indicated that 72.4% and 42.0% of insulin was released at 5 mM and 1 M H_2_O_2_, respectively, indicating that the drug release was H_2_O_2_-concentration dependent. Moreover, the insulin release increased in high-glucose conditions, demonstrating a glucose-sensitive release. Transdermal administration of the preparation maintained 3-h higher plasma insulin than the subcutaneous injection.

GLP-1 is an incretin hormone used for T2DM therapy due to its capacity to stimulate insulin secretion in a glucose-dependent manner. However, oral GLP-1 delivery is rapidly degraded by the enzyme DPP4.^420^ Therefore, the co-delivery of GLP-1 and DPP4 inhibitors seems rational. Shrestha et al. designed a nanocomposite formed by chitosan-modified porous silicon NPs and coated by an enteric polymer.^421^ The orally delivered NPs induced a 32% decrease in glycemia and approximately 6-fold augmentation in pancreatic insulin level compared to free combination. Another example is the study of Ma et al., who developed chitosan NPs-inlaid poly-l-lactide porous microparticles co-loaded with two antidiabetic agents, including GLP-1 and small interfering RNA (siRNA), to inhibit the expression of dipeptidyl peptidase-4 mRNA.^422^ Interestingly, the designed system (100–150 nm) was prepared using the supercritical carbon dioxide technology and was delivered through the pulmonary route. The codelivery system efficiently reduced hyperglycemia due to the sustained liberation of siRNA from NPs and the synergistic action of GLP-1.^422^

## Neurodegenerative diseases (NDs)

NDs represent the gradual deterioration of the function and structure of the neuron populations in the central nervous system (CNS).^423^ Immunocompetence reduction with age and chronic neuroinflammation are underlying causes of NDs, including Alzheimer’s disease (AD), Parkinson’s disease (PD), and amyotrophic lateral sclerosis (ALS).^424^ Insufficient clearance of the misfolded proteins can also induce NDs.^425^ For instance, the accumulation of β-amyloid, tau, and α-synuclein (α-syn) causes AD and PD, respectively.^426,427^ Additionally, neuronal degeneration and brain inflammation can be stimulated by the alterations of protein conformations aggregating into neurofibrils or oligomers and the resultant neuronal toxicity.^427,428^ ALS-neurodegeneration could be caused by various factors, including glutamate excitotoxicity, production of free radicals, cytoplasmic protein aggregates, SOD-1 enzymes, mitochondrial dysfunction, and the disruption of axonal transport processes through the accumulation of neurofilament intracellular aggregates.^429^

### Targets for ND therapy

Three categories are employed for the NDs therapy, e.g., treating AD using amyloid antibodies, cholinesterase inhibitors (ChEIs) and glutamate regulators, combating PD using dopamine supplements, decarboxylase inhibitors and dopamine agonists, and treating ALS using glutamate-receptor antagonist and free-radical scavenger.^430^ Nonetheless, developing an effective treatment approach against NDs remains challenging, owing to the unclear cause of onset and etiology and the blood-brain barrier (BBB) hindering brain drug delivery (Fig. 8).^431^

Amyloid proteins always induce neurotoxicity and likely could be a therapy target.^432^ NDs have a late onset and are often exacerbated by aging and neuronal loss.^433^ The aging and the missing neuronal decline in cellular homeostasis may be induced by DNA damage. Meanwhile, DNA injury is induced by the high level of ROS and mitochondria dysfunctions.^434^ The mitochondrial citric acid cycle is strengthened due to abnormal energy metabolism and dysfunctional mitochondria, intensifying neuroinflammation. As a result, mitochondria could be a potential target for treating AD.^435^ Second, the loss of neurons is affected by the ubiquitin-proteasome and the autophagy-lysosome pathways.^436^ So, these two pathways also could be used as therapy targets. E.g., the stimulation of the sigma-1 receptor activates autophagy, alleviates chronic CNS inflammation by reducing immune response, and is a promising therapeutic target against.^437^ Additionally, the protein Rho GTPase controls the development of the actin cytoskeleton in nerve cells and oxidative stress through the nuclear erythroid 2-related factor, significantly affecting cellular redox homeostasis.^438,439^

### Strategies for combinatorial ND therapy

#### Combining therapy strategies

Multiple pathways are always involved in NDs development; therefore, multi-drug therapy targets many molecular pathways rather than a single target.^440^ In 2014, Namzaric®, a combination of the AChE inhibitor donepezil and memantine, was approved to treat moderate to chronic AD.^441^ A hybrid compound containing the Rho kinase inhibitor fasudil and NRF2-triggers caffeic and ferulic acids was synthesized to treat ALS.^439^ The compound enabled NRF2 activation and promoted the expression of antioxidant response enzymes.^439^ The combined use of memantine and ChEIs for AD treatment is the most extensively researched and clinically proven effective.^370,371^ This combined treatment strategy slowed the functional and cognitive decline rate for more than one year compared to monotherapy. Additionally, compared to no treatment or ChEI monotherapy, the combined therapy lessened the development and severity of neurobehavioral symptoms, such as aggression and agitation, and demonstrated enhanced efficacy against the diseases at an early stage.^442^

Levodopa was launched in 1970 to treat PD motor symptoms, and five years later, the first combined product of levodopa and carbidopa was approved^443^. Afterward, various compounds were investigated to manage PD. However, only two drugs, riluzole and edaravone, were marketed to treat ALS. These two medicines could improve an individual’s quality of life. Also, their combination displayed elevated efficacy against ALS patients compared to monotherapy.^444^ Nevertheless, no effective disease-modifying treatments are obtainable for ALS; and most of the available combinations are used to alleviate symptoms rather than inhibit the disease development.

#### NP-mediated co-delivery

NPs were employed to deliver the therapeutics, i.e., chemical substances, genes, peptides and antibodies, to treat AD.^445–448^ For instance, a patent (CN110559454B) reported micelles modified using quadrupole superparamagnetic ferrite for AD-protein targeting and cathode ray tube for transferrin targeting for improving brain delivery.^449^ Yang et al. designed albumin NPs co-loading clioquinol (metal-ion chelator) and donepezil (acetylcholinesterase inhibitor) as potential synergistic therapy against AD.^450^ The drug combination could simultaneously restore the balance between amyloid-beta aggregates and acetylcholine. The NPs were modified with transcriptional activator protein and monosialotetrahexosylganglioside lipid to enhance brain targeting. After 30 days of intranasal administration, the NPs could rescue acetylcholine imbalance and reduce the aggregation of amyloid-beta, ameliorating spatial learning and memory function in AD mice. Associating the neuroprotective hormone, leptin, and the anti-inflammatory agent, pioglitazone, has been widely recommended for NDs treatment, including AD and ALS.^451,452^ Two active compounds, curcumin decomposing amyloid protein and superparamagnetic ferrite, were contained in the NPs. The results indicated that the NPs could increase the drug concentration at the target site and extend the accumulation time. Recently, two drugs, neuroprotective leptin and anti-inflammatory pioglitazone, were loaded in mesoporous silica NPs to treat ALS. The treatment study indicated that the co-loaded NPs could slow the disease progress and significantly improve the motor function in the TDP43^A315T^ model.^453^ Díaz-García et al. used mesoporous silica NPs to co-encapsulate leptin and pioglitazone. The study reported that the co-loaded NPs could slow the disease progression and significantly improve motor function in vivo.^453^

Multiple drug combinations have also been investigated for PD treatment. Levodopa is the gold standard of PD treatment.^454^ Usually, it is associated with carbidopa or benserazide to prevent its peripherical conversion into dopamine, which, unlike its precursor, levodopa, cannot cross the BBB.^454^ Also, long-term use of levodopa may induce dyskinesia.^455^ Yang et al. associated levodopa methyl ester with benserazide in one nanoplatform to sustainably release the two drugs.^456^ The NP-based combination significantly decreased the apomorphine-induced rotations in dyskinetic rats compared to the free combination. A recent patent (CN202010142569.7) proposed an NP made up of a lipid bilayer modified with cell-penetrating peptides and lactoferrin as the external shell to enclose the mesoporous silica NPs. This platform specifically co-delivers levodopa and curcumin to the brain to act synergistically. Levodopa relieves dyskinesia and curcumin exerts a neuroprotective effect.^457^ Recently, another group implemented polymeric micelles composed of polyethylene oxide and poly ε-caprolactone to co-deliver levodopa and curcumin as potential therapy for PD.^458^ The system was modified with glutathione to enhance brain delivery due to its specific binding in BBB.^459^

## Conclusions and perspectives

Combination-drug therapy allows synergistic therapy by simultaneously stimulating multiple pathways or enhancing the pharmacokinetic performance of one or more drugs. There are many mechanisms for synergistic therapy; however, not all therapeutic agents effectively work when combined.^460^ Chemical interference between therapeutic agents may reduce their combined action compared to the estimated sum of effort. Antagonism may occur if two compounds act competitively on the same target, reducing their combined activity. Therefore, verifying the relationship between their therapeutic index and synergy coefficient is necessary to ensure synergistic therapy when designing a combination therapy using two or more drugs. Computer-aided design can quickly and efficiently screen suitable drug combinations with synergistic effects. Moreover, the clinicians reported some potential drug combinations through clinical practices, and the combined treatment model has been utilized to treat various diseases (Tables 1–3).

Administering multiple drugs directly (mostly intravenously) always leads to compromised treatment efficacy because the drugs must cross many biological barriers before and after entering systemic circulation.^461–463^ Therefore, developing codelivery systems is vital for therapy as designing combination strategies.^464,465^ Over the years, NP-codelivery systems have been exploited with other therapeutic agents to treat various diseases. The NPs’ treatment efficacy could be enhanced by altering their physicochemical properties, i.e., diameter, morphology, surface charge and surface features, to improve their targetability to the diseased conditions, such as pH reduction, increased shear forces of blood flow, EPR effect, and highly expressed receptors on target tissues or cells. E.g., rod-shaped NPs could target the highly expressed caveolar protein on endothelial cells and improve cytosol delivery by reducing the endosomal entrapment. Furthermore, NPs could integrate different regimens for combinatorial treatment. For example, chemotherapy and photothermal therapy can effectively be combined using DDSs for treating cancer or AS. For specific diseases that are difficult to diagnose in real-time, co-delivering the diagnostic agent and the therapeutic drug to the lesion site enables real-time observation of the pathological process of the lesion site during treatment, integrating diagnosis and treatment.

Lipid NPs are often used carriers for co-delivery due to their ability to encapsulate various drugs and enhance the solubility of chemotherapeutic agents, efficiency, non-immunogenicity, and bio-compatibility.^466^ Over 20 liposomes and liposome-like NPs were approved for clinical use.^467–471^ Notably, a liposomal formulation containing daunorubicin and ara-C was approved to treat acute myeloid leukemia.^472^ The evidence demonstrates that liposomes are a promising carrier for codelivery. Interestingly, a carrier-free strategy termed the drug-delivering-drug (DDD) platform pioneered by He’s group was developed to improve co-delivery, using drug crystals of insoluble drugs as a carrier to deliver the second drug such as biopharmaceuticals and small molecular-weight compounds.^16,17,255,473^ The second drug RNAi and active proteins were absorbed into the drug crystals stabilized with cationic polymer or polyphenol through electrostatic or non-covalent interactions such as multi-hydrogen bonds.^474–476^ In contrast, a second small molecular-weight drug was incorporated into the drug crystals *via* a cocrystal-like approach.^11,477^ DDD’s most significant merit is the high drug-payload capacity of 70–100% (w/w), 20-fold more significant than the conventional drug carriers. DDD might represent a promising tactic for combinatorial therapy. Recently, drug-drug cocrystals, referred as solids that are crystalline single-phase materials composed of two or more different molecular and/or ionic compounds generally in a stoichiometric ratio which are neither solvates nor simple salts, are attracting increasing attention in the pharmaceutical field due to the ability to improve the in vivo fate and physicochemical properties of drugs, including solubility, permeability, hydration, tableting, mechanical strength, etc.^478,479^ Over 8 drug-drug cocrystals, e.g., Odomzo^®^ (cocrystal of sonidegib and phosphoric acid), Suglat^®^ (cocrystal of ipragliflozin and L-proline), and Entresto^®^ (cocrystal of valsartan and sacubitril), were marketed for the clinic. Cocrystals are developing as a potent combinatorial therapy strategy. E.g., Entresto®, consisting of the angiotensin receptor inhibitor valsartan and a neprilysin inhibitor prodrug (sacubitril), elevates the bioavailability of valsartan and reduces its dose. Incorporating nanotechnology into cocrystals may represent a new approach to designing novel NP-codelivery preparations.

Whereas a considerable number of NP-codelivery systems were reported, only one product (Vyxeos^®^) was approved, demonstrating a shallow translation rate. The poor translation efficacy may associate with the modest drug-loading ability of conventional polymer NPs. Liposomal formulations always demonstrate potent encapsulation ability for various drugs and, as a result, are often utilized for codelivery. Accordingly, pharmaceutical techniques with high drug-loading capacity, i.e., drug-drug cocrystals and liposome-like NPs, could be promising for NP-codelivery. However, it should still be noted that the composition ratio of different drugs in the co-loading system may not be equal to the drug ratio released by the NPs in the actual treatment, while the actual control system contributes to the synergistic effect. Therefore, establishing an analysis method that can precisely study the drug release is critical for the development of NP-codelivery.^480,481^ Moreover, the translation always involves enormous efforts, such as the initial selection of combination drugs and dosage forms, screening and characterization, the final large-scale batch production, and quality control. In addition, even though various NPs have been proven to target the diseased lesions and improve treatment efficacy, less than 1% of nanomedicines accumulate in the target site due to sequestration or clearance of RES and renal system, etc. This considerable non-targeted distribution of NPs may also cause side effects.^482–484^ Clinical desires should be the first driving force in developing combined DDSs or co-delivery preparations. Therefore, early clinical collaborative efforts should be undertaken to understand patient needs better and facilitate the development of novel combination DDSs. Interdisciplinary cooperation should be strengthened during the whole development and translation. The computer simulation systems could assist in optimizing the NP properties, including combinatorial drug ratio, drug-loading capacity, targetability, drug release profiles, and in vivo fate. Establishing effective in vitro and in vivo models is wanted to evaluate the combination DDSs regarding pharmacokinetics, biodistribution, and drug concentration at the target site. For industrialization and clinical use, unauthorized materials and complex preparation are not recommended.

## References

[1] He C, Tang Z, Tian H, Chen X (2016). Co-delivery of chemotherapeutics and proteins for synergistic therapy. Adv. Drug Deliv. Rev..

[2] Da Silva C (2016). Combinatorial prospects of nano-targeted chemoimmunotherapy. Biomaterials.

[3] Shim G (2017). Nanoformulation-based sequential combination cancer therapy. Adv. Drug Deliv. Rev..

[4] Zhang Z (2020). Overcoming cancer therapeutic bottleneck by drug repurposing. Signal Transduct. Target Ther..

[5] Shrestha B, Tang L, Romero G (2019). Nanoparticles‐mediated combination therapies for cancer treatment. Adv. Ther..

[6] Chen L (2018). Stepwise co-delivery of an enzyme and prodrug based on a multi-responsive nanoplatform for accurate tumor therapy. J. Mater. Chem. B..

[7] Guo M, Sun X, Chen J, Cai T (2021). Pharmaceutical cocrystals: A review of preparations, physicochemical properties and applications. Acta Pharm. Sin. B..

[8] Gurunathan S, Kang M-H, Qasim M, Kim J-H (2018). Nanoparticle-mediated combination therapy: Two-in-one approach for cancer. Int J. Mol. Sci..

[9] Ashrafizadeh M (2021). Hyaluronic acid-based nanoplatforms for doxorubicin: A review of stimuli-responsive carriers, co-delivery and resistance suppression. Carbohyd Polym..

[10] Wu R (2020). Combination chemotherapy of lung cancer–co-delivery of docetaxel prodrug and cisplatin using aptamer-decorated lipid–polymer hybrid nanoparticles. Drug Des. Dev. Ther..

[11] Li Y (2021). Cocrystallization-like strategy for the codelivery of hydrophobic and hydrophilic drugs in a single carrier material formulation. Chin. Chem. Lett..

[12] Baby T (2021). Microfluidic synthesis of curcumin loaded polymer nanoparticles with tunable drug loading and pH-triggered release. J. Colloid Inter. Sci..

[13] Cao Z (2019). pH-and enzyme-triggered drug release as an important process in the design of anti-tumor drug delivery systems. Biomed. Pharmacother..

[14] Liu R (2019). Theranostic nanoparticles with tumor-specific enzyme-triggered size reduction and drug release to perform photothermal therapy for breast cancer treatment. Acta Pharm. Sin. B..

[15] Xie X (2020). Ag nanoparticles cluster with pH‐triggered reassembly in targeting antimicrobial applications. Adv. Funct. Mater..

[16] Du X (2021). Cytosolic delivery of the immunological adjuvant Poly I: C and cytotoxic drug crystals via a carrier-free strategy significantly amplifies immune response. Acta Pharm. Sin. B..

[17] Teng C (2020). Intracellular codelivery of anti-inflammatory drug and anti-miR 155 to treat inflammatory disease. Acta Pharm. Sin. B.

[18] Zhang S, Langer R, Traverso G (2017). Nanoparticulate drug delivery systems targeting inflammation for treatment of inflammatory bowel disease. Nano Today.

[19] Krauss AC (2019). FDA Approval Summary:(Daunorubicin and cytarabine) liposome for injection for the treatment of adults with high-risk acute myeloid LeukemiaFDA Approval:(Daunorubicin and Cytarabine). Clin. Cancer Res..

[20] Couvreur P (2013). Nanoparticles in drug delivery: past, present and future. Adv. Drug Deliv. Rev..

[21] Birrenbach G, Speiser P (1976). Polymerized micelles and their use as adjuvants in immunology. J. Pharm. Sci..

[22] Chou LY, Ming K, Chan WC (2011). Strategies for the intracellular delivery of nanoparticles. Chem. Soc. Rev..

[23] Wang R (2021). Strategies for the design of nanoparticles: starting with long-circulating nanoparticles, from lab to clinic. Biomater. Sci..

[24] Stone NR, Bicanic T, Salim R, Hope W (2016). Liposomal amphotericin B (AmBisome®): a review of the pharmacokinetics, pharmacodynamics, clinical experience and future directions. Drugs.

[25] Lister J (1996). Amphotericin B lipid complex (Abelcet®) in the treatment of invasive mycoses: the North American experience. Eur. J. Haematol..

[26] Zylberberg C, Matosevic S (2016). Pharmaceutical liposomal drug delivery: a review of new delivery systems and a look at the regulatory landscape. Drug Deliv..

[27] Rivankar S (2014). An overview of doxorubicin formulations in cancer therapy. J. Can. Res Ther..

[28] Rizzardinl G, Pastecchia C, Vigevanl GM, Miiella AM (1997). Stealth liposomal doxorubicin or bleomycin/vincristine for the treatment of AIDS-related Kaposi’s sarcoma: 17. J. Acq Imm Def..

[29] Dinndorf PA (2007). FDA drug approval summary: pegaspargase (Oncaspar®) for the first-line treatment of children with acute lymphoblastic leukemia (ALL). Oncologist.

[30] Cammas S (1997). Thermo-responsive polymer nanoparticles with a core-shell micelle structure as site-specific drug carriers. J. Control Release.

[31] Stella B (2000). Design of folic acid‐conjugated nanoparticles for drug targeting. J. Pharm. Sci..

[32] Urits I (2020). A review of patisiran (ONPATTRO®) for the treatment of polyneuropathy in people with hereditary transthyretin amyloidosis. NeurTher.

[33] Thapa RK, Kim JO (2023). Nanomedicine-based commercial formulations: Current developments and future prospects. J. Pharm. Investig..

[34] Yuan S, Chen H (2019). Mathematical rules for synergistic, additive, and antagonistic effects of multi-drug combinations and their application in research and development of combinatorial drugs and special medical food combinations. Food Sci. Hum. Well..

[35] Chen D (2015). Systematic synergy modeling: understanding drug synergy from a systems biology perspective. BMC Syst. Biol..

[36] Niu J, Straubinger RM, Mager DE (2019). Pharmacodynamic Drug–Drug Interactions. Clin. Pharm. Ther..

[37] Caesar LK, Cech NB (2019). Synergy and antagonism in natural product extracts: when 1+ 1 does not equal 2. Nat. Prod. Rep..

[38] Nøhr-Nielsen A (2020). Pharmacodynamic modelling reveals synergistic interaction between docetaxel and SCO-101 in a docetaxel-resistant triple negative breast cancer cell line. Eur. J. Pharm. Sci..

[39] Rodríguez-Vázquez GO (2023). Synergistic interactions of cytarabine-adavosertib in leukemic cell lines proliferation and metabolomic endpoints. Biomed. Pharmacother..

[40] Liu Z (2015). Pharmacokinetic synergy from the taxane extract of Taxus chinensis improves the bioavailability of paclitaxel. Phytomedicine.

[41] Wang H, Huang Y (2020). Combination therapy based on nano codelivery for overcoming cancer drug resistance. Med Drug Discov..

[42] Tardi P (2009). In vivo maintenance of synergistic cytarabine: daunorubicin ratios greatly enhances therapeutic efficacy. Leuk. Res..

[43] Foucquier J, Guedj M (2015). Analysis of drug combinations: current methodological landscape. Pharm. Res Perspect..

[44] Wooten DJ (2021). MuSyC is a consensus framework that unifies multi-drug synergy metrics for combinatorial drug discovery. Nat. Commun..

[45] Duarte D, Vale N (2022). Evaluation of synergism in drug combinations and reference models for future orientations in oncology. Curr Res Pharm. Drug Discov..

[46] Vakil V, Trappe W (2019). Drug combinations: mathematical modeling and networking methods. Pharmaceutics.

[47] Li Y (2021). Protease-triggered bioresponsive drug delivery for the targeted theranostics of malignancy. Acta Pharm. Sin. B..

[48] Bejarano L, Jordāo MJ, Joyce JA (2021). Therapeutic targeting of the tumor microenvironment. Cancer Discov..

[49] Xiao Y, Yu D (2021). Tumor microenvironment as a therapeutic target in cancer. Pharm. Therapeut..

[50] Romanini A (2003). First-line chemotherapy with epidoxorubicin, paclitaxel, and carboplatin for the treatment of advanced epithelial ovarian cancer patients. Gynecol. Oncol..

[51] Ye F (2018). Advances in nanotechnology for cancer biomarkers. Nano Today.

[52] Jin C, Wang K, Oppong-Gyebi A, Hu J (2020). Application of nanotechnology in cancer diagnosis and therapy-a mini-review. Int J. Med Sci..

[53] Chaturvedi VK, Singh A, Singh VK, Singh MP (2019). Cancer nanotechnology: A new revolution for cancer diagnosis and therapy. Curr. Drug Metab..

[54] Yang S (2021). Paying attention to tumor blood vessels: cancer phototherapy assisted with nano delivery strategies. Biomaterials.

[55] Liu J (2018). A DNA-based nanocarrier for efficient gene delivery and combined cancer therapy. Nano Lett..

[56] Qian K, Yan B, Xiong Y (2021). The application of chemometrics for efficiency enhancement and toxicity reduction in cancer treatment with combined therapy. Curr. Drug Deliv..

[57] Partridge AH, Burstein HJ, Winer EP (2001). Side effects of chemotherapy and combined chemohormonal therapy in women with early-stage breast cancer. JNCI Monogr..

[58] Lebaron S (1988). Chemotherapy side effects in pediatric oncology patients: Drugs, age, and sex as risk factors. Med Pediatr. Oncol..

[59] Lee A, Djamgoz MB (2018). Triple negative breast cancer: emerging therapeutic modalities and novel combination therapies. Cancer Treat. Rev..

[60] Sang W, Zhang Z, Dai Y, Chen X (2019). Recent advances in nanomaterial-based synergistic combination cancer immunotherapy. Chem. Soc. Rev..

[61] Walsh JH, Karnes W, Cuttitta F, Walker A (1991). Autocrine growth factors and solid tumor malignancy. West. J. Med..

[62] Drozdov I (2009). Autoregulatory effects of serotonin on proliferation and signaling pathways in lung and small intestine neuroendocrine tumor cell lines. Cancer.

[63] Semenza GL (2012). Hypoxia-inducible factors: mediators of cancer progression and targets for cancer therapy. Trends Pharm. Sci..

[64] Shang P (2021). VEGFR2-targeted antibody fused with IFNαmut regulates the tumor microenvironment of colorectal cancer and exhibits potent anti-tumor and anti-metastasis activity. Acta Pharm. Sin. B..

[65] Intlekofer AM, Finley LW (2019). Metabolic signatures of cancer cells and stem cells. Nat. Metab..

[66] Zhu J, Thompson CB (2019). Metabolic regulation of cell growth and proliferation. Nat. Rev. Mol. Cell Biol..

[67] Lee D-Y, Song M-Y, Kim E-H (2021). Role of oxidative stress and Nrf2/keap1 signaling in colorectal cancer: Mechanisms and therapeutic perspectives with phytochemicals. Antioxidants.

[68] Taguchi K, Yamamoto M (2020). The KEAP1–NRF2 system as a molecular target of cancer treatment. Cancers.

[69] Cha H-Y (2016). Downregulation of Nrf2 by the combination of TRAIL and Valproic acid induces apoptotic cell death of TRAIL-resistant papillary thyroid cancer cells via suppression of Bcl-xL. Cancer Lett..

[70] Foo BJ-A, Eu JQ, Hirpara JL, Pervaiz S (2021). Interplay between mitochondrial metabolism and cellular redox state dictates cancer cell survival. Oxid. Med. Cell Longev..

[71] Missiroli S (2020). Cancer metabolism and mitochondria: Finding novel mechanisms to fight tumours. EBioMedicine.

[72] Singh P, Lim B (2022). Targeting apoptosis in cancer. Curr. Oncol. Rep..

[73] Singh R, Letai A, Sarosiek K (2019). Regulation of apoptosis in health and disease: the balancing act of BCL-2 family proteins. Nat. Rev. Mol. Cell Biol..

[74] Castle VP (1993). Expression of the apoptosis-suppressing protein bcl-2, in neuroblastoma is associated with unfavorable histology and N-myc amplification. Am. J. Pathol..

[75] Raffo AJ (1995). Overexpression of bcl-2 protects prostate cancer cells from apoptosis in vitro and confers resistance to androgen depletion in vivo. Cancer Res..

[76] Wang M, Su P (2018). The role of the Fas/FasL signaling pathway in environmental toxicant-induced testicular cell apoptosis: An update. Syst. Biol. Reprod. Med..

[77] Ivanisenko NV (2022). Regulation of extrinsic apoptotic signaling by c-FLIP: towards targeting cancer networks. Trends Cancer.

[78] Zheng Y, Ma L, Sun Q (2021). Clinically-relevant ABC transporter for anti-cancer drug resistance. Front Pharmacol..

[79] Wang JQ (2021). ATP‐binding cassette (ABC) transporters in cancer: A review of recent updates. JEBM.

[80] Gupta SK, Singh P, Ali V, Verma M (2020). Role of membrane-embedded drug efflux ABC transporters in the cancer chemotherapy. Oncol. Rev..

[81] Sharma P, Allison James P (2015). The future of immune checkpoint therapy. Science.

[82] Passardi A, Canale M, Valgiusti M, Ulivi P (2017). Immune checkpoints as a target for colorectal cancer treatment. Int. J. Mol. Sci..

[83] Anderson TS (2022). Disrupting cancer angiogenesis and immune checkpoint networks for improved tumor immunity. Semin Cancer Biol..

[84] Li N (2022). Adverse and unconventional reactions related to immune checkpoint inhibitor therapy for cancer. Int Immunopharmacol..

[85] Khair DO (2019). Combining immune checkpoint inhibitors: Established and emerging targets and strategies to improve outcomes in melanoma. Front Immunol..

[86] Bonati L, Tang L (2021). Cytokine engineering for targeted cancer immunotherapy. Curr. Opin. Chem. Biol..

[87] Mughees M (2022). Chemokines and cytokines: Axis and allies in prostate cancer pathogenesis. Semin Cancer Biol..

[88] Malik D, Mahendiratta S, Kaur H, Medhi B (2021). Futuristic approach to cancer treatment. Gene.

[89] Baskar R, Lee KA, Yeo R, Yeoh K-W (2012). Cancer and radiation therapy: current advances and future directions. Int J. Med. Sci..

[90] DeVita VT, Chu E (2008). A history of cancer chemotherapy. Cancer Res..

[91] Wahida A (2023). The coming decade in precision oncology: six riddles. Nat. Rev. Cancer.

[92] Liu R (2022). Advances of nanoparticles as drug delivery systems for disease diagnosis and treatment. Chin. Chem. Lett..

[93] Deshpande PP, Biswas S, Torchilin VP (2013). Current trends in the use of liposomes for tumor targeting. Nanomedicine.

[94] He K, Tang M (2018). Safety of novel liposomal drugs for cancer treatment: Advances and prospects. Chem. Biol. Interact..

[95] Saraf S (2020). Advances in liposomal drug delivery to cancer: An overview. J. Drug Deliv. Sci. Tec..

[96] Fan Y, Zhang Q (2013). Development of liposomal formulations: From concept to clinical investigations. Asian J. Pharm. Sci..

[97] Sousa I (2018). Liposomal therapies in oncology: does one size fit all?. Cancer Chemoth Pharm..

[98] Cooper TM (2020). Phase I/II study of CPX-351 followed by fludarabine, cytarabine, and granulocyte-colony stimulating factor for children with relapsed acute myeloid leukemia: a report from the Children’s Oncology Group. J. Clin. Oncol..

[99] Feldman EJ (2008). Phase I study of a liposomal carrier (CPX-351) containing a synergistic, fixed molar ratio of cytarabine (Ara-C) and daunorubicin (DNR) in advanced leukemias. Blood.

[100] Lin TL (2021). Older adults with newly diagnosed high-risk/secondary AML who achieved remission with CPX-351: phase 3 post hoc analyses. Blood Adv..

[101] Lim W-S (2010). Leukemia-selective uptake and cytotoxicity of CPX-351, a synergistic fixed-ratio cytarabine: daunorubicin formulation, in bone marrow xenografts. Leuk. Res..

[102] Blagosklonny MV (2008). “Targeting the absence” and therapeutic engineering for cancer therapy. Cell Cycle.

[103] Sun Y (2021). Co-delivery of chemotherapeutic drugs and cell cycle regulatory agents using nanocarriers for cancer therapy. Sci. China Mater..

[104] Li F (2019). Co-delivery of VEGF siRNA and Etoposide for Enhanced Anti-angiogenesis and Anti-proliferation Effect via Multi-functional Nanoparticles for Orthotopic Non-Small Cell Lung Cancer Treatment. Theranostics.

[105] Nakamura H, Takada K (2021). Reactive oxygen species in cancer: Current findings and future directions. Cancer Sci..

[106] Kohan R (2020). Reactive oxygen species in cancer: A paradox between pro-and anti-tumour activities. Cancer Chemoth Pharm..

[107] ArulJothi K (2022). Implications of reactive oxygen species in lung cancer and exploiting it for therapeutic interventions. Med Oncol..

[108] Sarmiento-Salinas FL (2021). Reactive oxygen species: Role in carcinogenesis, cancer cell signaling and tumor progression. Life Sci..

[109] Ghoneum A (2020). Redox homeostasis and metabolism in cancer: a complex mechanism and potential targeted therapeutics. Int J. Mol. Sci..

[110] Antunes F, Cadenas E (2001). Cellular titration of apoptosis with steady state concentrations of H2O2: submicromolar levels of H2O2 induce apoptosis through Fenton chemistry independent of the cellular thiol state. Free Radic. Biol. Med..

[111] Tang J (2016). Co-delivery of doxorubicin and P-gp inhibitor by a reduction-sensitive liposome to overcome multidrug resistance, enhance anti-tumor efficiency and reduce toxicity. Drug Deliv..

[112] Mirzaei S (2022). Advances in understanding the role of P-gp in doxorubicin resistance: Molecular pathways, therapeutic strategies, and prospects. Drug Discov..

[113] Wang Y (2022). Paclitaxel derivative-based liposomal nanoplatform for potentiated chemo-immunotherapy. J. Control Release.

[114] Zhang J (2021). Small molecules regulating reactive oxygen species homeostasis for cancer therapy. Med Res Rev..

[115] Zong Q (2022). Self-amplified chain-shattering cinnamaldehyde-based poly (thioacetal) boosts cancer chemo-immunotherapy. Acta Biomater..

[116] Boafo GF (2022). Targeted co-delivery of daunorubicin and cytarabine based on the hyaluronic acid prodrug modified liposomes. Chin. Chem. Lett..

[117] Lv Y (2018). Nanoplatform assembled from a CD44-targeted prodrug and smart liposomes for dual targeting of tumor microenvironment and cancer cells. Acs Nano..

[118] Xiao Q (2022). Liposome-based anchoring and core-encapsulation for combinatorial cancer therapy. Chin. Chem. Lett..

[119] Mei K-C (2020). Liposomal Delivery of Mitoxantrone and a Cholesteryl Indoximod Prodrug Provides Effective Chemo-immunotherapy in Multiple Solid Tumors. ACS Nano..

[120] Xiao Q (2022). Improving cancer immunotherapy via co-delivering checkpoint blockade and thrombospondin-1 downregulator. Acta Pharma Sin. B..

[121] Yu J (2021). Combining PD-L1 inhibitors with immunogenic cell death triggered by chemo-photothermal therapy via a thermosensitive liposome system to stimulate tumor-specific immunological response. Nanoscale.

[122] Mukherjee A, Bisht B, Dutta S, Paul MK (2022). Current advances in the use of exosomes, liposomes, and bioengineered hybrid nanovesicles in cancer detection and therapy. Acta Pharm. Sin..

[123] Xu Z (2020). Pathological findings of COVID-19 associated with acute respiratory distress syndrome. Lancet Resp. Med..

[124] Zheng Y (2021). Recent progress in sono-photodynamic cancer therapy: From developed new sensitizers to nanotechnology-based efficacy-enhancing strategies. Acta Pharm. Sin. B..

[125] Tarantino P (2022). Antibody–drug conjugates: Smart chemotherapy delivery across tumor histologies. CA Cancer J. Clin..

[126] Fu Z (2022). Antibody drug conjugate: the “biological missile” for targeted cancer therapy. Signal Transduct. Target Ther..

[127] Baah S, Laws M, Rahman KM (2021). Antibody–drug conjugates—A tutorial review. Molecules.

[128] Baron J, Wang ES (2018). Gemtuzumab ozogamicin for the treatment of acute myeloid leukemia. Expert Rev. Clin. Phar..

[129] Jin Y (2022). Stepping forward in antibody-drug conjugate development. Pharmacol. Therapeut..

[130] Shi F (2022). Disitamab vedotin: a novel antibody-drug conjugates for cancer therapy. Drug Deliv..

[131] Deeks ED (2021). Disitamab vedotin: first approval. Drugs.

[132] Nicolaou KC, Rigol S (2019). The role of organic synthesis in the emergence and development of antibody–drug conjugates as targeted cancer therapies. Angew. Chem. Int Ed..

[133] Wiedemeyer WR (2022). ABBV-011, a novel, calicheamicin-based antibody–drug conjugate, targets SEZ6 to eradicate small cell lung cancer tumors. Mol. Cancer Ther..

[134] Jabr-Milane LS, van Vlerken LE, Yadav S, Amiji MM (2008). Multi-functional nanocarriers to overcome tumor drug resistance. Cancer Treat. Rev..

[135] Baguley BC (2010). Multiple drug resistance mechanisms in cancer. Mol. Biotechnol..

[136] Iyer AK, Duan Z, Amiji MM (2014). Nanodelivery Systems for Nucleic Acid Therapeutics in Drug Resistant Tumors. Mol. Pharm..

[137] Tonissen KF, Poulsen S-A (2021). Carbonic anhydrase XII inhibition overcomes P-glycoprotein-mediated drug resistance: A potential new combination therapy in cancer. Cancer Drug Resist..

[138] Chen S, Deng J, Zhang L-M (2021). Cationic nanoparticles self-assembled from amphiphilic chitosan derivatives containing poly (amidoamine) dendrons and deoxycholic acid as a vector for co-delivery of doxorubicin and gene. Carbohyd Polym..

[139] Lee MJ (2012). Sequential application of anticancer drugs enhances cell death by rewiring apoptotic signaling networks. Cell.

[140] Vickers NJ (2017). Animal communication: when i’m calling you, will you answer too?. Curr. Biol..

[141] Wu M (2018). Photoresponsive nanovehicle for two independent wavelength light-triggered sequential release of P-gp shRNA and doxorubicin to optimize and enhance synergistic therapy of multidrug-resistant cancer. ACS Appl. Mater. Interfaces.

[142] Fares J (2020). Molecular principles of metastasis: a hallmark of cancer revisited. Signal Transduct. Target Ther..

[143] Dana H (2021). CAR-T cells: Early successes in blood cancer and challenges in solid tumors. Acta Pharm. Sin. B..

[144] Tang T (2019). Harnessing the layer-by-layer assembly technique to design biomaterials vaccines for immune modulation in translational applications. Biomater. Sci..

[145] Garris CS (2018). Successful anti-PD-1 cancer immunotherapy requires T cell-dendritic cell crosstalk involving the cytokines IFN-γ and IL-12. Immunity.

[146] Sun D (2022). A cyclodextrin-based nanoformulation achieves co-delivery of ginsenoside Rg3 and quercetin for chemo-immunotherapy in colorectal cancer. Acta Pharm. Sin. B..

[147] Kroemer G, Galluzzi L, Kepp O, Zitvogel L (2013). Immunogenic cell death in cancer therapy. Annu Rev. Immunol.

[148] Zhu M (2018). Co-delivery of tumor antigen and dual toll-like receptor ligands into dendritic cell by silicon microparticle enables efficient immunotherapy against melanoma. J. Control Release.

[149] Ashburn TT, Thor KB (2004). Drug repositioning: identifying and developing new uses for existing drugs. Nat. Rev. Drug Discov..

[150] Turanli B (2021). Systems biology based drug repositioning for development of cancer therapy. Semin Cancer Biol..

[151] Turanli B (2019). Discovery of therapeutic agents for prostate cancer using genome-scale metabolic modeling and drug repositioning. EBioMedicine.

[152] Mohammadi E (2020). Applications of genome-wide screening and systems biology approaches in drug repositioning. Cancers.

[153] Wu Z, Li W, Liu G, Tang Y (2018). Network-Based Methods for Prediction of Drug-Target Interactions. Front Pharmacol..

[154] Wang P, Shen Y, Zhao L (2020). Chitosan nanoparticles loaded with aspirin and 5-fluororacil enable synergistic antitumour activity through the modulation of NF-κB/COX-2 signalling pathway. IET Nanobiotechnol..

[155] Song Y (2022). Recent advances in targeted stimuli-responsive nano-based drug delivery systems combating atherosclerosis. Chin. Chem. Lett..

[156] Murray CJ, Lopez AD (1997). Alternative projections of mortality and disability by cause 1990–2020: Global Burden of Disease Study. Lancet.

[157] Hopkins PN, Williams RR (1981). A survey of 246 suggested coronary risk factors. Atherosclerosis.

[158] Kannel WB, Wilson PW (1995). An update on coronary risk factors. Med Clin. N. Am..

[159] Saigusa R, Winkels H, Ley K (2020). T cell subsets and functions in atherosclerosis. Nat. Rev. Cardiol..

[160] Allahverdian S (2018). Smooth muscle cell fate and plasticity in atherosclerosis. Cardiovascular Res..

[161] Wolf D, Ley K (2019). Immunity and inflammation in atherosclerosis. Circ. Res..

[162] Paone S, Baxter AA, Hulett MD, Poon IK (2019). Endothelial cell apoptosis and the role of endothelial cell-derived extracellular vesicles in the progression of atherosclerosis. Cell Mol. Life Sci..

[163] Zahid MK (2021). Role of macrophage autophagy in atherosclerosis: modulation by bioactive compounds. Biochem J..

[164] Custodio-Chablé SJ, Lezama RA, Reyes-Maldonado E (2020). Platelet activation as a trigger factor for inflammation and atherosclerosis. Cirugía y. cirujanos..

[165] Lordan R, Tsoupras A, Zabetakis I (2021). Platelet activation and prothrombotic mediators at the nexus of inflammation and atherosclerosis: Potential role of antiplatelet agents. Blood Rev..

[166] Marchio P (2019). Targeting early atherosclerosis: a focus on oxidative stress and inflammation. Oxid. Med Cell Longev..

[167] Raggi P (2018). Role of inflammation in the pathogenesis of atherosclerosis and therapeutic interventions. Atherosclerosis.

[168] Volobueva A, Zhang D, Grechko AV, Orekhov AN (2018). Foam cell formation and cholesterol trafficking and metabolism disturbances in atherosclerosis. Cor et. Vasa.

[169] Kwak B, Mulhaupt F, Myit S, Mach F (2000). Statins as a newly recognized type of immunomodulator. Nat. Med..

[170] Gotto AM (2001). Statin therapy: where are we? Where do we go next?. Am. J. Cardiol..

[171] Grundy SM (2002). Alternative approaches to cholesterol-lowering therapy. Am. J. Cardiol..

[172] Jia J (2021). A systematic review and meta-analysis on the efficacy of statins in the treatment of atherosclerosis. Ann. Palliat. Med..

[173] Alder M (2020). A meta-analysis assessing additional LDL-C reduction from addition of a bile acid sequestrant to statin therapy. Am. J. Med..

[174] Lee M (2022). Association between intensity of low-density lipoprotein cholesterol reduction with statin-based therapies and secondary stroke prevention: a meta-analysis of randomized clinical trials. JAMA Neurol..

[175] Saxon DR, Eckel RH (2016). Statin intolerance: a literature review and management strategies. Prog. Cardiovasc Dis..

[176] Okada K (2012). Long-term effects of ezetimibe-plus-statin therapy on low-density lipoprotein cholesterol levels as compared with double-dose statin therapy in patients with coronary artery disease. Atherosclerosis.

[177] Park S-W (2013). Intestinal and hepatic niemann-pick c1-like 1. Diabetes Metab. J..

[178] Ah Y-M, Jeong M, Choi HD (2022). Comparative safety and efficacy of low-or moderate-intensity statin plus ezetimibe combination therapy and high-intensity statin monotherapy: A meta-analysis of randomized controlled studies. Plos one.

[179] Hibi K (2018). Effects of ezetimibe-statin combination therapy on coronary atherosclerosis in acute coronary syndrome. Circ. J..

[180] Hong N (2018). Comparison of the effects of ezetimibe-statin combination therapy on major adverse cardiovascular events in patients with and without diabetes: a meta-analysis. Endocrinol. Metab..

[181] Sabatine MS (2019). PCSK9 inhibitors: clinical evidence and implementation. Nat. Rev. Cardiol..

[182] Gallego-Colon E, Daum A, Yosefy C (2020). Statins and PCSK9 inhibitors: A new lipid-lowering therapy. Eur. J. Pharmacol..

[183] Pradhan AD, Aday AW, Rose LM, Ridker PM (2018). Residual inflammatory risk on treatment with PCSK9 inhibition and statin therapy. Circulation.

[184] Wallentin L (2009). Ticagrelor versus clopidogrel in patients with acute coronary syndromes. N. Engl. J. Med..

[185] Wiviott SD (2007). Prasugrel versus clopidogrel in patients with acute coronary syndromes. N. Engl. J. Med..

[186] Olie RH, van der Meijden PE, Ten Cate H (2018). The coagulation system in atherothrombosis: Implications for new therapeutic strategies. Thromb. Haemost..

[187] Khan SU (2022). PCSK9 inhibitors and ezetimibe with or without statin therapy for cardiovascular risk reduction: a systematic review and network meta-analysis. Brit Med J..

[188] Rached F, Santos RD (2021). Beyond statins and PCSK9 inhibitors: updates in management of familial and refractory hypercholesterolemias. Curr. Cardiol. Rep..

[189] Kong P (2022). Inflammation and atherosclerosis: signaling pathways and therapeutic intervention. Signal Transduct. Target Ther..

[190] Samuel M, Tardif J-C (2021). Lessons learned from large Cardiovascular Outcome Trials targeting inflammation in cardiovascular disease (CANTOS, CIRT, COLCOT and LoDoCo2). Future Cardiol..

[191] Everett BM (2020). Inhibition of interleukin-1β and reduction in atherothrombotic cardiovascular events in the CANTOS trial. J. Am. Coll. Cardiol..

[192] Xepapadaki E (2020). Τhe antioxidant function of HDL in atherosclerosis. Angiology.

[193] Assmann G, Gotto AM (2004). HDL cholesterol and protective factors in atherosclerosis. Circulation.

[194] Maisch B, Alter P (2018). Treatment options in myocarditis and inflammatory cardiomyopathy. Herz.

[195] Chen J (2020). High density lipoprotein mimicking nanoparticles for atherosclerosis. Nano Converg..

[196] Ou L-c, Zhong S, Ou J-s, Tian J-w (2021). Application of targeted therapy strategies with nanomedicine delivery for atherosclerosis. Acta Pharm. Sin..

[197] Motamed S, Hosseini Karimi SN, Hooshyar M, Mehdinavaz Aghdam R (2021). Advances in nanocarriers as drug delivery systems in Atherosclerosis therapy. JUFGNSM.

[198] He J (2020). Shuttle/sink model composed of β-cyclodextrin and simvastatin-loaded discoidal reconstituted high-density lipoprotein for enhanced cholesterol efflux and drug uptake in macrophage/foam cells. J. Mater. Chem. B..

[199] He J (2023). Reactive oxygen species (ROS)-responsive size-reducible nanoassemblies for deeper atherosclerotic plaque penetration and enhanced macrophage-targeted drug delivery. Bioact. Mater..

[200] He J (2022). Anchoring β-CD on simvastatin-loaded rHDL for selective cholesterol crystals dissolution and enhanced anti-inflammatory effects in macrophage/foam cells. Eur. J. Pharm. Biopharm..

[201] Vickers KC (2011). MicroRNAs are transported in plasma and delivered to recipient cells by high-density lipoproteins. Nat. Cell Biol..

[202] Tabet F (2014). HDL-transferred microRNA-223 regulates ICAM-1 expression in endothelial cells. Nat. Commun..

[203] Wiese CB (2019). Dual inhibition of endothelial miR-92a-3p and miR-489-3p reduces renal injury-associated atherosclerosis. Atherosclerosis.

[204] Schultz JR (2000). Role of LXRs in control of lipogenesis. Gene Dev..

[205] Im S-S, Osborne TF (2011). Liver x receptors in atherosclerosis and inflammation. Circ. Res..

[206] Guo Y (2018). Synthetic High-Density Lipoprotein-Mediated Targeted Delivery of Liver X Receptors Agonist Promotes Atherosclerosis Regression. EBioMedicine.

[207] Xiao Q (2021). Biological drug and drug delivery-mediated immunotherapy. Acta Pharm. Sin. B..

[208] Sheng J (2022). Targeted therapy of atherosclerosis by zeolitic imidazolate framework-8 nanoparticles loaded with losartan potassium via simultaneous lipid-scavenging and anti-inflammation. J. Mater. Chem. B..

[209] Zhao R (2022). A ROS-Responsive Simvastatin Nano-Prodrug and its Fibronectin-Targeted Co-Delivery System for Atherosclerosis Treatment. ACS Appl Mater. Interfaces.

[210] Opriessnig P, Silbernagel G, Krassnig S, Reishofer G (2018). Magnetic resonance microscopy diffusion tensor imaging of collagen fibre bundles stabilizing an atherosclerotic plaque of the common carotid artery. Eur. Heart J..

[211] Li X (2022). Liposomal codelivery of inflammation inhibitor and collagen protector to the plaque for effective anti-atherosclerosis. Chin. Chem. Lett..

[212] Humbert M (2006). Pulmonary arterial hypertension in France: results from a national registry. Am. J. Resp. Crit. Care..

[213] Maron BA (2021). Pulmonary arterial hypertension: diagnosis, treatment, and novel advances. Am. J. Resp. Crit. Care..

[214] Naeije R, Richter MJ, Rubin LJ (2022). The physiological basis of pulmonary arterial hypertension. Eur. Respir. J..

[215] Zoulikha M, Huang F, Wu Z, He W (2022). COVID-19 inflammation and implications in drug delivery. J. Control Release.

[216] Mclaughlin VV (2014). Treatment goals of pulmonary hypertension. J. Am. Coll. Cardiol..

[217] Galiè N (2019). Risk stratification and medical therapy of pulmonary arterial hypertension. Eur. Respir. J..

[218] Evans CE (2021). Endothelial cells in the pathogenesis of pulmonary arterial hypertension. Eur. Respir. J..

[219] Kunder, S. K. Pharmacotherapy of Pulmonary Arterial Hypertension. In *Introduction to Basics of Pharmacology and Toxicology* (eds Paul, A., Anandabaskar, N., & Mathaiyan, J., Raj, G. M.) (Springer, Singapore, 2021).

[220] Dai Y (2019). Immunotherapy of endothelin-1 receptor type A for pulmonary arterial hypertension. J. Am. Coll. Cardiol..

[221] de Lima-Seolin BG (2018). Bucindolol attenuates the vascular remodeling of pulmonary arteries by modulating the expression of the endothelin-1 A receptor in rats with pulmonary arterial hypertension. Biomed. Pharmacother..

[222] Lan NS, Massam BD, Kulkarni SS, Lang CC (2018). Pulmonary arterial hypertension: pathophysiology and treatment. Diseases.

[223] Hoeper MM (2021). Switching to riociguat versus maintenance therapy with phosphodiesterase-5 inhibitors in patients with pulmonary arterial hypertension (REPLACE): a multicentre, open-label, randomised controlled trial. Lancet Resp. Med..

[224] Prins KW (2019). Repurposing medications for treatment of pulmonary arterial hypertension: what’s old is new again. J. Am. Heart Assoc..

[225] Beghetti M (2019). Treatment of pediatric pulmonary arterial hypertension: A focus on the NO‐sGC‐cGMP pathway. Pediatr. Pulm..

[226] Angalakuditi M (2010). Treatment patterns and resource utilization and costs among patients with pulmonary arterial hypertension in the United States. J. Med Econ..

[227] Galie N, Palazzini M, Manes A (2010). Pulmonary arterial hypertension: from the kingdom of the near-dead to multiple clinical trial meta-analyses. Eur. Heart J..

[228] Yang Y (2020). Discovery of highly selective and orally available benzimidazole-based phosphodiesterase 10 inhibitors with improved solubility and pharmacokinetic properties for treatment of pulmonary arterial hypertension. Acta Pharm. Sin. B..

[229] Halliday SJ (2018). Clinical and genetic associations with prostacyclin response in pulmonary arterial hypertension. Pulm. Circ..

[230] Gąsecka A (2021). Prostacyclin analogues inhibit platelet reactivity, extracellular vesicle release and thrombus formation in patients with pulmonary arterial hypertension. J. Clin. Med..

[231] Lambers C (2018). Mechanism of anti-remodelling action of treprostinil in human pulmonary arterial smooth muscle cells. PLoS One.

[232] Lindegaard Pedersen M (2020). The prostacyclin analogue treprostinil in the treatment of pulmonary arterial hypertension. Basic Clin. Pharmacol..

[233] Spaczyńska M, Rocha SF, Oliver E (2020). Pharmacology of pulmonary arterial hypertension: an overview of current and emerging therapies. ACS Pharm. Transl..

[234] Nakamura K (2019). Current treatment strategies and nanoparticle-mediated drug delivery systems for pulmonary arterial hypertension. Int J. Mol. Sci..

[235] Bai Y, Sun L, Hu S, Wei Y (2011). Combination therapy in pulmonary arterial hypertension: a meta-analysis. Cardiology.

[236] Fox BD (2016). Combination therapy for pulmonary arterial hypertension: a systematic review and meta-analysis. Can. J. Cardiol..

[237] Ghofrani H-A, Humbert M (2014). The role of combination therapy in managing pulmonary arterial hypertension. Eur. Respir. Rev..

[238] Galiè N (2015). Initial use of ambrisentan plus tadalafil in pulmonary arterial hypertension. N. Engl. J. Med..

[239] Lajoie AC (2016). Combination therapy versus monotherapy for pulmonary arterial hypertension: a meta-analysis. Lancet Resp. Med..

[240] Sitbon O (2016). Initial dual oral combination therapy in pulmonary arterial hypertension. Eur. Respir. J..

[241] Gruenig E (2009). Acute hemodynamic effects of single‐dose sildenafil when added to established bosentan therapy in patients with pulmonary arterial hypertension: results of the COMPASS‐1 study. J. Clin. Pharmacol..

[242] McLaughlin VV (2006). Randomized study of adding inhaled iloprost to existing bosentan in pulmonary arterial hypertension. Am. J. Resp. Crit. Care..

[243] Said K (2014). Riociguat: patent-1 study. Glob. Cardiol. Sci. Pract..

[244] McLaughlin V (2014). Effect of Bosentan and Sildenafil Combination Therapy on Morbidity and Mortality in Pulmonary Arterial Hypertension (PAH): Results From the COMPASS-2 Study. Chest.

[245] Maron BA, Galiè N (2016). Diagnosis, treatment, and clinical management of pulmonary arterial hypertension in the contemporary era: a review. JAMA Cardiol..

[246] Shimokawa H, Satoh K (2015). 2015 ATVB Plenary Lecture: translational research on rho-kinase in cardiovascular medicine. Arterioscl Throm Vas..

[247] Gupta V (2013). Liposomal fasudil, a rho-kinase inhibitor, for prolonged pulmonary preferential vasodilation in pulmonary arterial hypertension. J. Control Release.

[248] Rashid J (2018). Fasudil and DETA NONOate, loaded in a peptide-modified liposomal carrier, slow PAH progression upon pulmonary delivery. Mol. Pharm..

[249] Gupta N (2017). Cocktail of superoxide dismutase and fasudil encapsulated in targeted liposomes slows PAH progression at a reduced dosing frequency. Mol. Pharm..

[250] Qi L (2019). Fasudil dichloroacetate (FDCA), an orally available agent with potent therapeutic efficiency on monocrotaline-induced pulmonary arterial hypertension rats. Bioorg. Med Chem. Lett..

[251] Yang Y (2020). Investigational pharmacotherapy and immunotherapy of pulmonary arterial hypertension: An update. Biomed. Pharmacother..

[252] Costa J (2018). Inflammatory response of pulmonary artery smooth muscle cells exposed to oxidative and biophysical stress. Inflammation.

[253] Mamazhakypov A (2021). The role of chemokines and chemokine receptors in pulmonary arterial hypertension. Brit J. Pharmacol..

[254] Dreymueller D (2014). Smooth muscle cells relay acute pulmonary inflammation via distinct ADAM17/ErbB axes. J. Immunol..

[255] Teng C (2022). Targeted delivery of baicalein-p53 complex to smooth muscle cells reverses pulmonary hypertension. J. Control Release.

[256] Savai R (2014). Pro-proliferative and inflammatory signaling converge on FoxO1 transcription factor in pulmonary hypertension. Nat. Med..

[257] Tschöpe C, Cooper LT, Torre-Amione G, Van Linthout S (2019). Management of myocarditis-related cardiomyopathy in adults. Circ. Res..

[258] Basso C (2022). Myocarditis. N. Engl. J. Med..

[259] Caforio ALP, Malipiero G, Marcolongo R, Iliceto S (2017). Myocarditis: A Clinical Overview. Curr. Cardiol. Rep..

[260] Caforio AL (2013). Current state of knowledge on aetiology, diagnosis, management, and therapy of myocarditis: a position statement of the European Society of Cardiology Working Group on Myocardial and Pericardial Diseases. Eur. Heart J..

[261] Ammirati E (2018). Clinical presentation and outcome in a contemporary cohort of patients with acute myocarditis: multicenter Lombardy registry. Circulation.

[262] Seko Y (1995). Restricted usage of T cell receptor V alpha-V beta genes in infiltrating cells in the hearts of patients with acute myocarditis and dilated cardiomyopathy. J. Clin. Invest..

[263] Godeny EK, Gauntt C (1987). In situ immune autoradiographic identification of cells in heart tissues of mice with coxsackievirus B3-induced myocarditis. Am. J. Pathol..

[264] Hua X, Song J (2019). Immune cell diversity contributes to the pathogenesis of myocarditis. Heart Fail Rev..

[265] Seko Y (1991). Expression of perforin in infiltrating cells in murine hearts with acute myocarditis caused by coxsackievirus B3. Circulation.

[266] Leone O, Pieroni M, Rapezzi C, Olivotto I (2019). The spectrum of myocarditis: from pathology to the clinics. Virchows Arch..

[267] Rivadeneyra L (2018). Role of neutrophils in CVB3 infection and viral myocarditis. J. Mol. Cell Cardiol..

[268] Alu A (2020). The role of lysosome in regulated necrosis. Acta Pharm. Sin. B..

[269] Jensen LD, Marchant DJ (2016). Emerging pharmacologic targets and treatments for myocarditis. Pharm. Therapeut..

[270] Myers JM (2016). Cardiac myosin-Th17 responses promote heart failure in human myocarditis. JCI insight.

[271] Higashitani K (2022). Rituximab and mepolizumab combination therapy for glucocorticoid-resistant myocarditis related to eosinophilic granulomatosis with polyangiitis. Mod. Rheumatol. Case.

[272] Winter M-P (2018). Immunomodulatory treatment for lymphocytic myocarditis—a systematic review and meta-analysis. Heart Fail Rev..

[273] Wojnicz R (2001). Randomized, placebo-controlled study for immunosuppressive treatment of inflammatory dilated cardiomyopathy: two-year follow-up results. Circulation.

[274] Frustaci A, Russo MA, Chimenti C (2009). Randomized study on the efficacy of immunosuppressive therapy in patients with virus-negative inflammatory cardiomyopathy: the TIMIC study. Eur. Heart J..

[275] Campochiaro C (2021). Efficacy and safety of methotrexate for the treatment of autoimmune virus-negative myocarditis: a case series. J. Clin. Rheumatol..

[276] Song T, Jones DM, Homsi Y (2017). Therapeutic effect of anti-IL-5 on eosinophilic myocarditis with large pericardial effusion. BMJ Case Rep..

[277] Yen C-Y (2019). Role of intravenous immunoglobulin therapy in the survival rate of pediatric patients with acute myocarditis: A systematic review and meta-analysis. Sci. Rep..

[278] Wei X, Fang Y, Hu H (2020). Glucocorticoid and immunoglobulin to treat viral fulminant myocarditis. Eur. Heart J..

[279] Hamada H (2019). Efficacy of primary treatment with immunoglobulin plus ciclosporin for prevention of coronary artery abnormalities in patients with Kawasaki disease predicted to be at increased risk of non-response to intravenous immunoglobulin (KAICA): a randomised controlled, open-label, blinded-endpoints, phase 3 trial. Lancet.

[280] Li JH, Li TT, Wu XS, Zeng DL (2021). Effect of gamma globulin combined with creatine phosphate on viral myocarditis. Am. J. Transl. Res..

[281] Lee G (2017). Curcumin attenuates the scurfy-induced immune disorder, a model of IPEX syndrome, with inhibiting Th1/Th2/Th17 responses in mice. Phytomedicine.

[282] Liu R (2018). Curcumin alleviates isoproterenol-induced cardiac hypertrophy and fibrosis through inhibition of autophagy and activation of mTOR. Eur. Rev. Med Pharm. Sci..

[283] Luthra PM, Singh R, Chandra R (2001). Therapeutic uses ofCurcuma longa (turmeric). Indian J. Clin. Bioche..

[284] Hernández M, Wicz S, Santamaría MH, Corral RS (2018). Curcumin exerts anti-inflammatory and vasoprotective effects through amelioration of NFAT-dependent endothelin-1 production in mice with acute Chagas cardiomyopathy. Mem. I Oswaldo Cruz..

[285] Hernández M, Wicz S, Corral RS (2016). Cardioprotective actions of curcumin on the pathogenic NFAT/COX-2/prostaglandin E2 pathway induced during Trypanosoma cruzi infection. Phytomedicine.

[286] Hernández M (2021). Dual chemotherapy with benznidazole at suboptimal dose plus curcumin nanoparticles mitigates Trypanosoma cruzi-elicited chronic cardiomyopathy. Parasitol. Int..

[287] Wu M-Y (2021). Pharmacological insights into autophagy modulation in autoimmune diseases. Acta Pharma Sin. B..

[288] McInnes IB, Schett G (2017). Pathogenetic insights from the treatment of rheumatoid arthritis. Lancet.

[289] Siouti E, Andreakos E (2019). The many facets of macrophages in rheumatoid arthritis. Biochem Pharmacol..

[290] Butola LK, Anjanker A, Vagga A, Kaple MN (2020). Endogenous factor and pathophysiology of rheumatoid arthritis: an autoimmune disease from decades. Int J. Cur Res Rev..

[291] Koenders MI, van den Berg WB (2015). Novel therapeutic targets in rheumatoid arthritis. Trends Pharm. Sci..

[292] Alghasham A, Rasheed Z (2014). Therapeutic targets for rheumatoid arthritis: Progress and promises. Autoimmunity.

[293] Pirmardvand Chegini S, Varshosaz J, Taymouri S (2018). Recent approaches for targeted drug delivery in rheumatoid arthritis diagnosis and treatment. Artif. Cell Nanomed. B..

[294] Kesharwani D, Paliwal R, Satapathy T, Paul SD (2019). Rheumatiod arthritis: an updated overview of latest therapy and drug delivery. J. Pharmacopunct..

[295] Wang S (2020). Recent Advances in Nanotheranostics for Treat‐to‐Target of Rheumatoid Arthritis. Adv. Health. Mater..

[296] Wang Q, Qin X, Fang J, Sun X (2021). Nanomedicines for the treatment of rheumatoid arthritis: State of art and potential therapeutic strategies. Acta Pharm. Sin. B..

[297] Drosos AA, Pelechas E, Voulgari PV (2020). Treatment strategies are more important than drugs in the management of rheumatoid arthritis. Clin. Rheumatol..

[298] Donahue KE (2019). Comparative effectiveness of combining MTX with biologic drug therapy versus either MTX or biologics alone for early rheumatoid arthritis in adults: a systematic review and network meta-analysis. J. Gen. Intern Med..

[299] Yang M (2017). Nanotherapeutics relieve rheumatoid arthritis. J. Control Release.

[300] Yuan F (2012). Development of macromolecular prodrug for rheumatoid arthritis. Adv. Drug Deliv. Rev..

[301] Buch M, Bingham S, Bryer D, Emery P (2007). Long-term infliximab treatment in rheumatoid arthritis: subsequent outcome of initial responders. Rheumatology.

[302] Listing J (2005). Infections in patients with rheumatoid arthritis treated with biologic agents. Arthritis Rheum.-US..

[303] Dolati S (2016). Utilization of nanoparticle technology in rheumatoid arthritis treatment. Biomed. Pharmacother..

[304] Yu Z (2020). Nanomedicines for the delivery of glucocorticoids and nucleic acids as potential alternatives in the treatment of rheumatoid arthritis. Wires Nanomed. Nanobi..

[305] Yu K (2021). Layered dissolving microneedles as a need-based delivery system to simultaneously alleviate skin and joint lesions in psoriatic arthritis. Acta Pharm. Sin. B..

[306] Janakiraman K (2020). Development of methotrexate and minocycline loaded nanoparticles for the effective treatment of rheumatoid arthritis. AAPS PharmSciTech..

[307] Chen X (2019). Targeted hexagonal Pd nanosheet combination therapy for rheumatoid arthritis via the photothermal controlled release of MTX. J. Mater. Chem. B..

[308] Wang Y (2019). Enhanced therapeutic effect of RGD-modified polymeric micelles loaded with low-dose methotrexate and nimesulide on rheumatoid arthritis. Theranostics.

[309] Son AR (2015). Direct chemotherapeutic dual drug delivery through intra-articular injection for synergistic enhancement of rheumatoid arthritis treatment. Sci. Rep..

[310] Shen Q (2020). Sinomenine hydrochloride loaded thermosensitive liposomes combined with microwave hyperthermia for the treatment of rheumatoid arthritis. Int J. Pharm..

[311] Park JS (2012). The use of anti-COX2 siRNA coated onto PLGA nanoparticles loading dexamethasone in the treatment of rheumatoid arthritis. Biomaterials.

[312] Duan W, Li H (2018). Combination of NF-kB targeted siRNA and methotrexate in a hybrid nanocarrier towards the effective treatment in rheumatoid arthritis. J. Nanobiotechnol..

[313] Wang Q (2017). Targeting NF-kB signaling with polymeric hybrid micelles that co-deliver siRNA and dexamethasone for arthritis therapy. Biomaterials.

[314] Hao F (2019). Hybrid micelles containing methotrexate-conjugated polymer and co-loaded with microRNA-124 for rheumatoid arthritis therapy. Theranostics.

[315] Yin N (2020). A novel indomethacin/methotrexate/MMP-9 siRNA in situ hydrogel with dual effects of anti-inflammatory activity and reversal of cartilage disruption for the synergistic treatment of rheumatoid arthritis. Nanoscale.

[316] DK P (2002). Inflanain bowel diease. N. Engl. J. Med..

[317] Abraham C, Cho JH (2009). Mechanisms of disease. N. Engl. J. Med..

[318] Graham DB, Xavier RJ (2020). Pathway paradigms revealed from the genetics of inflammatory bowel disease. Nature.

[319] Cui G, Yuan A (2018). A systematic review of epidemiology and risk factors associated with Chinese inflammatory bowel disease. Front Med..

[320] Axelrad JE, Cadwell KH, Colombel J-F, Shah SC (2021). The role of gastrointestinal pathogens in inflammatory bowel disease: a systematic review. Ther. Adv. Gastroenter..

[321] Ahlawat S (2021). Inflammatory bowel disease: tri-directional relationship between microbiota, immune system and intestinal epithelium. Crit. Rev. Microbiol..

[322] Veenbergen S (2019). IL-10 signaling in dendritic cells controls IL-1β-mediated IFNγ secretion by human CD4+ T cells: relevance to inflammatory bowel disease. Mucosal Immunol..

[323] Bernardo D, Chaparro M, Gisbert JP (2018). Human intestinal dendritic cells in inflammatory bowel diseases. Mol. Nutr. Food Res..

[324] Leppkes M, Neurath M (2020). Cytokines in inflammatory bowel diseases–update 2020. Pharm. Res..

[325] Lee A (2017). Dexamethasone-loaded polymeric nanoconstructs for monitoring and treating inflammatory bowel disease. Theranostics.

[326] Na YR, Stakenborg M, Seok SH, Matteoli G (2019). Macrophages in intestinal inflammation and resolution: a potential therapeutic target in IBD. Nat. Rev. Gastro Hepat..

[327] Fredericks E, Watermeyer G (2019). De-escalation of biological therapy in inflammatory bowel disease: Benefits and risks. S Afr. Med J..

[328] Peyrin-Biroulet L (2015). Selecting therapeutic targets in inflammatory bowel disease (STRIDE): determining therapeutic goals for treat-to-target. Am. J. Gastroenterol..

[329] Xiao Q (2022). The effects of protein corona on in vivo fate of nanocarriers. Adv. Drug Deliv. Rev..

[330] Sandborn WJ (2003). Strategies for targeting tumour necrosis factor in IBD. Best. Pr. Res Cl. Gastroenterol.

[331] Privitera G (2021). Combination therapy in inflammatory bowel disease–from traditional immunosuppressors towards the new paradigm of dual targeted therapy. Autoimmun. Rev..

[332] Papa A (2009). Biological therapies for inflammatory bowel disease: controversies and future options. Expert Rev. Clin. Pharm..

[333] Sokol H (2010). Usefulness of co-treatment with immunomodulators in patients with inflammatory bowel disease treated with scheduled infliximab maintenance therapy. Gut.

[334] Dohos D (2021). Systematic review with meta‐analysis: the effects of immunomodulator or biological withdrawal from mono‐or combination therapy in inflammatory bowel disease. Aliment Pharm. Ther..

[335] van Schaik T (2014). Influence of combination therapy with immune modulators on anti-TNF trough levels and antibodies in patients with IBD. Inflamm. Bowel Dis..

[336] Vos ACW (2012). Regulatory macrophages induced by infliximab are involved in healing in vivo and in vitro. Inflamm. Bowel Dis..

[337] Colombel J-F (2019). Combination therapy with infliximab and azathioprine improves infliximab pharmacokinetic features and efficacy: A post hoc analysis. Clin. Gastroenterol. H..

[338] Fan X, Ding X, Zhang Q-Y (2020). Hepatic and intestinal biotransformation gene expression and drug disposition in a dextran sulfate sodium-induced colitis mouse model. Acta Pharm. Sin. B..

[339] Xiao B (2018). TNFα gene silencing mediated by orally targeted nanoparticles combined with interleukin-22 for synergistic combination therapy of ulcerative colitis. J. Control Release.

[340] Aib S (2022). pH-sensitive liposomes for colonic co-delivery of mesalazine and curcumin for the treatment of ulcerative colitis. J. Drug Deliv. Sci. Tec..

[341] Desai N, Momin M (2020). Colon targeted bioadhesive pellets of curcumin and cyclosporine for improved management of inflammatory bowel disease. Drug Deliv. Transl. Res..

[342] Liu P (2021). Receptor-mediated targeted drug delivery systems for treatment of inflammatory bowel disease: Opportunities and emerging strategies. Acta Pharm. Sin. B..

[343] Xu X (2019). Efficient and targeted drug/siRNA co-delivery mediated by reversibly crosslinked polymersomes toward anti-inflammatory treatment of ulcerative colitis (UC). Nano Res..

[344] Bilek R, Dvořáková M, Grimmichova T, Jiskra J (2020). Iodine, thyroglobulin and thyroid gland. Physiol. Res..

[345] Macovei M-L, Azis Ű, Gheorghe AG, Burcea M (2021). A systematic review of euthyroid Graves’ disease. Exp. Ther. Med..

[346] Wiersinga WM (2019). Graves’ disease: can it be cured?. Endocrinol. Metab..

[347] Leung AM, Braverman LE (2014). Consequences of excess iodine. Nat. Rev. Endocrinol..

[348] Lane LC, Cheetham TD, Perros P, Pearce SH (2020). New therapeutic horizons for Graves’ hyperthyroidism. Endocr. Rev..

[349] Lee HJ (2017). Cd40 signaling in graves disease is mediated through canonical and noncanonical thyroidal nuclear factor κ b activation. Endocrinology.

[350] Smith TJ, Hegedüs L (2016). Graves’ disease. N. Engl. J. Med..

[351] Marín-Sánchez A (2019). Regulation of TSHR expression in the thyroid and thymus may contribute to TSHR tolerance failure in Graves’ disease patients via two distinct mechanisms. Front Immunol..

[352] Wang X-X, Wang X-X, Chen T (2019). Association between the CD40 rs1883832 polymorphism and Graves’ disease risk: a meta-analysis. Excli J..

[353] Casto C (2021). Hashimoto’s thyroiditis and graves’ disease in genetic syndromes in pediatric age. Genes.

[354] Zawadzka-Starczewska K (2022). Actual associations between HLA haplotype and Graves’ disease development. J. Clin. Med..

[355] Speletas M (2022). The rs1883832 Polymorphism (CD40-1C> T) Affects the Intensity of IgA Responses after BNT162b2 Vaccination. Int J. Mol. Sci..

[356] Brinkhaus M (2022). The Fab region of IgG impairs the internalization pathway of FcRn upon Fc engagement. Nat. Commun..

[357] Chu K-Y, Yu H-S, Yu S (2022). Current and innovated managements for autoimmune bullous skin disorders: An overview. J. Clin. Med..

[358] Wyckoff SL, Hudson KE (2021). Targeting the neonatal Fc receptor (FcRn) to treat autoimmune diseases and maternal-fetal immune cytopenias. Transfusion.

[359] Hsieh A (2019). Liver enzyme profile and progression in association with thyroid autoimmunity in Graves’ disease. Endocrinol. Diabetes Metab..

[360] Lin J-D (2016). Serum BAFF and thyroid autoantibodies in autoimmune thyroid disease. Clin. Chim. Acta.

[361] Lane LC (2019). Analysis of BAFF gene polymorphisms in UK Graves’ disease patients. Clin. Endocrinol..

[362] Lin J-D (2016). Analysis of associations of human BAFF gene polymorphisms with autoimmune thyroid diseases. PLoS One.

[363] Faulkner J, Varadharajan K, Choudhury N (2019). A UK reported case of Graves’ disease with thyroid hemiagenesis. BMJ Case Rep. Cp..

[364] RossDouglas S (2016). 2016 American Thyroid Association guidelines for diagnosis and management of hyperthyroidism and other causes of thyrotoxicosis. Thyroid.

[365] Brito JP (2020). Patterns of use, efficacy, and safety of treatment options for patients with Graves’ disease: a nationwide population-based study. Thyroid.

[366] Kahaly GJ (2020). Management of Graves thyroidal and extrathyroidal disease: an update. J. Clin. Endocr. Metab..

[367] Lillevang-Johansen M (2019). Duration of hyperthyroidism and lack of sufficient treatment are associated with increased cardiovascular risk. Thyroid.

[368] Avery JC, Hoffmann PR (2018). Selenium, selenoproteins, and immunity. Nutrients.

[369] Gallo D (2020). Immunomodulatory effect of vitamin D and its potential role in the prevention and treatment of thyroid autoimmunity: a narrative review. J. Endocrinol..

[370] Winther KH, Rayman MP, Bonnema SJ, Hegedüs L (2020). Selenium in thyroid disorders—essential knowledge for clinicians. Nat. Rev. Endocrinol..

[371] Xu M-Y (2015). Vitamin D and Graves’ disease: a meta-analysis update. Nutrients.

[372] Bouillon R (2019). Skeletal and extraskeletal actions of vitamin D: current evidence and outstanding questions. Endocr. rev..

[373] Dankers W, Colin EM, Van Hamburg JP, Lubberts E (2016). Vitamin D in autoimmunity: molecular mechanisms and therapeutic potential. Front Immunol..

[374] Gallo D (2022). Add-on effect of selenium and vitamin D combined supplementation in early control of graves’ disease hyperthyroidism during methimazole treatment. Front Endocrinol. (Lausanne).

[375] Xie C, He C, Gao J, Jia S (2020). Efficacy and safety of tripterygium glycosides in the treatment of hyperthyroidism: A systemic review and meta-analysis. Med. (Baltim.)..

[376] Choudhury AA, Rajeswari VD (2021). Gestational diabetes mellitus-A metabolic and reproductive disorder. Biomed. Pharmacother..

[377] Mukhtar Y, Galalain A, Yunusa U (2020). A modern overview on diabetes mellitus: a chronic endocrine disorder. Eur. J. Cell Biol..

[378] Bolli GB, Porcellati F, Lucidi P, Fanelli CG (2021). The physiological basis of insulin therapy in people with diabetes mellitus. Diabetes Res Clin. Pr..

[379] Jwad SM, AL-Fatlawi HY (2022). Types of diabetes and their effect on the immune system. J. Adv. Pharm. Pract..

[380] Powers MA (2020). Diabetes self-management education and support in adults with type 2 diabetes: a consensus report of the American Diabetes Association, the Association of Diabetes Care & Education Specialists, the Academy of Nutrition and Dietetics, the American Academy of Family Physicians, the American Academy of PAs, the American Association of Nurse Practitioners, and the American Pharmacists Association. Diabetes Care..

[381] Syed FZ (2022). Type 1 diabetes mellitus. Ann. Intern Med..

[382] Padhi S, Nayak AK, Behera A (2020). Type II diabetes mellitus: a review on recent drug based therapeutics. Biomed. Pharmacother..

[383] Fu J, Retnakaran R (2022). The life course perspective of gestational diabetes: An opportunity for the prevention of diabetes and heart disease in women. EClinicalMedicine.

[384] Liu J (2019). Weight retention at six weeks postpartum and the risk of gestational diabetes mellitus in a second pregnancy. BMC Pregnancy Child..

[385] Sousa M, Bruges-Armas J (2020). Monogenic diabetes: genetics and relevance on diabetes mellitus personalized medicine. Curr. Diab Rep..

[386] Skoczek D, Dulak J, Kachamakova-Trojanowska N (2021). Maturity onset diabetes of the young—new approaches for disease modelling. Int. J. Mol. Sci..

[387] Dewanjee S (2021). The emerging role of HDACs: pathology and therapeutic targets in diabetes mellitus. Cells.

[388] Sun X, Wang L, Obayomi SB, Wei Z (2021). Epigenetic regulation of β cell identity and dysfunction. Front Endocrinol..

[389] Sonthalia M (2022). Histone deacetylase inhibitors as antidiabetic agents: Advances and opportunities. Eur. J. Pharm..

[390] Makkar R, Behl T, Arora S (2020). Role of HDAC inhibitors in diabetes mellitus. Curr. Res Transl. Med..

[391] Kaimala S (2022). Epigenetic modifications in pancreas development, diabetes, and therapeutics. Med Res Rev..

[392] Zhang W (2021). Rosmarinic acid prevents refractory bacterial pneumonia through regulating Keap1/Nrf2-mediated autophagic pathway and mitochondrial oxidative stress. Free Radic. Biol. Med..

[393] David JA, Rifkin WJ, Rabbani PS, Ceradini DJ (2017). The Nrf2/Keap1/ARE pathway and oxidative stress as a therapeutic target in type II diabetes mellitus. J. Diabetes Res..

[394] Liu Q, Gao Y, Ci X (2019). Role of Nrf2 and its activators in respiratory diseases. Oxid. Med. Cell Longev..

[395] Li G, Zhang Y, Fan Z (2021). Cellular signal transduction pathways involved in acute lung injury induced by intestinal ischemia-reperfusion. Oxid. Med Cell Longev..

[396] Adelusi TI (2020). Keap1/Nrf2/ARE signaling unfolds therapeutic targets for redox imbalanced-mediated diseases and diabetic nephropathy. Biomed. pharmacother..

[397] Casas AI (2020). On the clinical pharmacology of reactive oxygen species. Pharm. Rev..

[398] Juszczyk G (2021). Chronic stress and oxidative stress as common factors of the pathogenesis of depression and Alzheimer’s disease: The role of antioxidants in prevention and treatment. Antioxidants.

[399] Mahboob A (2022). An investigation into the potential action of polyphenols against human Islet Amyloid Polypeptide aggregation in type 2 diabetes. Int J. Biol. Macromol..

[400] Ulasov AV, Rosenkranz AA, Georgiev GP, Sobolev AS (2022). Nrf2/Keap1/ARE signaling: Towards specific regulation. L Sci..

[401] Krüger-Genge A, Blocki A, Franke R-P, Jung F (2019). Vascular endothelial cell biology: an update. Int. J. Mol. Sci..

[402] Wołoszyn-Durkiewicz A, Myśliwiec M (2019). The prognostic value of inflammatory and vascular endothelial dysfunction biomarkers in microvascular and macrovascular complications in type 1 diabetes. J. Pediatr. Endocr. Diabetes Met..

[403] Sahu B, Bal NC (2022). Adipokines from white adipose tissue in regulation of whole body energy homeostasis. Biochimie.

[404] Mamdouh M (2017). Adipokines: potential therapeutic targets for vascular dysfunction in type II diabetes mellitus and obesity. J. Diabetes Res..

[405] Pathak V (2019). Therapies for type 1 diabetes: current scenario and future perspectives. Exp. Clin. Endocrinol..

[406] Rosner B, Roman-Urrestarazu A (2019). Health-related quality of life in paediatric patients with Type 1 diabetes mellitus using insulin infusion systems. A systematic review and meta-analysis. PLoS One.

[407] Association AD (2010). Diagnosis and classification of diabetes mellitus. Diabetes care..

[408] Taheri S (2020). Effect of intensive lifestyle intervention on bodyweight and glycaemia in early type 2 diabetes (DIADEM-I): an open-label, parallel-group, randomised controlled trial. Lancet Diabetes Endocrinol.

[409] Yang W (2022). Dorzagliatin add-on therapy to metformin in patients with type 2 diabetes: a randomized, double-blind, placebo-controlled phase 3 trial. Nat. Med..

[410] Cosentino F (2020). 2019 ESC Guidelines on diabetes, pre-diabetes, and cardiovascular diseases developed in collaboration with the EASD: The Task Force for diabetes, pre-diabetes, and cardiovascular diseases of the European Society of Cardiology (ESC) and the European Association for the Study of Diabetes (EASD). Eur. Heart J..

[411] Rena G, Hardie DG, Pearson ER (2017). The mechanisms of action of metformin. Diabetologia.

[412] Chen L, Zhang J, Yang R, Feng L (2021). 117-LB: Glucokinase Activator Dorzagliatin (HMS5552) Regulates GLP-1 Release in T2D Patients and Is Synergistic with Sitagliptin and Empagliflozin in Optimizing Beta-Cell Function. Diabetes.

[413] Zhu D (2018). Dorzagliatin monotherapy in Chinese patients with type 2 diabetes: a dose-ranging, randomised, double-blind, placebo-controlled, phase 2 study. Lancet Diabetes Endo..

[414] Vallon V, Thomson SC (2020). The tubular hypothesis of nephron filtration and diabetic kidney disease. Nat. Rev. Nephrol..

[415] Yakovleva T (2019). Comparison of the urinary glucose excretion contributions of SGLT2 and SGLT1: A quantitative systems pharmacology analysis in healthy individuals and patients with type 2 diabetes treated with SGLT2 inhibitors. Diabetes Obes. Metab..

[416] Tentolouris A (2019). SGLT2 inhibitors: a review of their antidiabetic and cardioprotective effects. Int. J. Environ. Res. Public Health.

[417] Tahara A, Takasu T (2019). SGLT2 inhibitor ipragliflozin alone and combined with pioglitazone prevents progression of nonalcoholic steatohepatitis in a type 2 diabetes rodent model. Physiol. Rep..

[418] Sarkar S, Kabir ME, Kalita J, Manna P (2023). Mesoporous silica nanoparticles: Drug delivery vehicle for antidiabetic molecules. ChemBioChem.

[419] Xu B (2017). H2O2-responsive mesoporous silica nanoparticles integrated with microneedle patches for the glucose-monitored transdermal delivery of insulin. J. Mater. Chem. B..

[420] Müller TD (2019). Glucagon-like peptide 1 (GLP-1). Mol. Metab..

[421] Shrestha N (2016). Oral hypoglycaemic effect of GLP-1 and DPP4 inhibitor based nanocomposites in a diabetic animal model. J. Control Release.

[422] Ma J (2020). Thymoquinone inhibits the proliferation and invasion of esophageal cancer cells by disrupting the AKT/GSK‐3β/Wnt signaling pathway via PTEN upregulation. Phytother. Res..

[423] Lo Vasco VR (2023). Emerging roles of signal transduction pathways in neurodegenerative diseases. Hunting new possible therapeutic molecular targets. OBM Geriatrics..

[424] Scheiblich H, Trombly M, Ramirez A, Heneka MT (2020). Neuroimmune connections in aging and neurodegenerative diseases. Trends immunol..

[425] Schmidt MF, Gan ZY, Komander D, Dewson G (2021). Ubiquitin signalling in neurodegeneration: mechanisms and therapeutic opportunities. Cell Death Differ..

[426] Lane CA, Hardy J, Schott JM (2018). Alzheimer’s disease. Eur. J. Neurol..

[427] Soto C, Pritzkow S (2018). Protein misfolding, aggregation, and conformational strains in neurodegenerative diseases. Nat. Neurosci..

[428] Tanaka M, Toldi J, Vécsei L (2020). Exploring the etiological links behind neurodegenerative diseases: inflammatory cytokines and bioactive kynurenines. Int J. Mol. Sci..

[429] Smeyers J, Mordes DA (2023). Running up that pill for amyotrophic lateral sclerosis. Brain.

[430] Lamptey RNL (2022). A review of the common neurodegenerative disorders: current therapeutic approaches and the potential role of nanotherapeutics. Int. J. Mol. Sci..

[431] Akhtar A (2021). Neurodegenerative diseases and effective drug delivery: A review of challenges and novel therapeutics. J. Control Release.

[432] Li D, Liu C (2022). Conformational strains of pathogenic amyloid proteins in neurodegenerative diseases. Nat. Rev. Neurosci..

[433] Culig L, Chu X, Bohr VA (2022). Neurogenesis in aging and age-related neurodegenerative diseases. Ageing Res. Rev..

[434] Shadfar S, Brocardo M, Atkin JD (2022). The complex mechanisms by which neurons die following DNA damage in neurodegenerative diseases. Int. J. Mol. Sci..

[435] Rauf A (2022). Neuroinflammatory markers: Key indicators in the pathology of neurodegenerative diseases. Molecules.

[436] Sahoo S, Padhy AA, Kumari V, Mishra P (2022). Role of ubiquitin–proteasome and autophagy-lysosome pathways in α-synuclein aggregate clearance. Mol. Neurobiol..

[437] Bogár F, Fülöp L, Penke B (2022). Novel therapeutic target for Prevention of neurodegenerative diseases: Modulation of neuroinflammation with Sig-1R ligands. Biomolecules.

[438] Bai X, Bian Z, Zhang M (2022). Targeting the Nrf2 signaling pathway using phytochemical ingredients: a novel therapeutic road map to combat neurodegenerative diseases. Phytomedicine.

[439] Martín-Cámara O (2022). Multitarget Hybrid Fasudil Derivatives as a New Approach to the Potential Treatment of Amyotrophic Lateral Sclerosis. J. Med Chem..

[440] Bloomingdale P (2022). Hallmarks of neurodegenerative disease: A systems pharmacology perspective. CPT.

[441] Pardo-Moreno T (2022). Therapeutic approach to Alzheimer’s disease: Current treatments and new perspectives. Pharmaceutics.

[442] Kabir MT (2020). Combination drug therapy for the management of Alzheimer’s disease. Int. J. Mol. Sci..

[443] Prasad EM, Hung S-Y (2021). Current therapies in clinical trials of Parkinson’s disease: A 2021 update. Pharmaceuticals.

[444] Dash RP, Babu RJ, Srinivas NR (2018). Two decades-long journey from riluzole to edaravone: revisiting the clinical pharmacokinetics of the only two amyotrophic lateral sclerosis therapeutics. Clin. Pharmacokinet..

[445] Aillaud I, Funke SA (2022). Tau aggregation inhibiting peptides as potential therapeutics for Alzheimer disease. Cell Mol. Neurobiol..

[446] Arora S, Kanekiyo T, Singh J (2022). Functionalized nanoparticles for brain targeted BDNF gene therapy to rescue Alzheimer’s disease pathology in transgenic mouse model. Int. J. Biol. Sci..

[447] Oumata N (2022). Molecular mechanisms in Alzheimer’s disease and related potential treatments such as structural target convergence of antibodies and simple organic molecules. Eur. J. Med. Chem..

[448] Shi M, Chu F, Zhu F, Zhu J (2022). Impact of anti-amyloid-β monoclonal antibodies on the pathology and clinical profile of Alzheimer’s disease: a focus on aducanumab and lecanemab. Front Aging Neurosci..

[449] Cao Y, Zhang R (2022). The application of nanotechnology in treatment of Alzheimer’s disease. Front Bioeng. Biotech..

[450] Yang H (2020). A novel targeted and high‐efficiency nanosystem for combinational therapy for Alzheimer’s disease. Adv. Sci..

[451] Ferrer‐Donato A, Contreras A, Fernandez P, Fernandez‐Martos CM (2022). The potential benefit of leptin therapy against amyotrophic lateral sclerosis (ALS). Brain Behav..

[452] Liu Y (2020). Enhanced anti-amyloid effect of combined leptin and pioglitazone in APP/PS1 transgenic mice. Curr. Alzheimer Res..

[453] Díaz-García D (2022). Design of mesoporous silica nanoparticles for the treatment of amyotrophic lateral sclerosis (ALS) with a therapeutic cocktail based on leptin and pioglitazone. Acs Biomater. Sci. Eng..

[454] Alabrahim OAA, Azzazy HME-S (2022). Polymeric nanoparticles for dopamine and levodopa replacement in Parkinson’s disease. Nanoscale Adv..

[455] Tran TN, Vo TN, Frei K, Truong DD (2018). Levodopa-induced dyskinesia: clinical features, incidence, and risk factors. J. Neural Transm..

[456] Yang X (2012). Controlled-release levodopa methyl ester/benserazide-loaded nanoparticles ameliorate levodopa-induced dyskinesia in rats. Inte J. Nanomed..

[457] Cheng G (2022). Anti-Parkinsonian therapy: strategies for crossing the blood–brain barrier and nano-biological effects of nanomaterials. Nano-micro Lett..

[458] Mogharbel BF (2022). Biodegradable nanoparticles loaded with levodopa and curcumin for treatment of Parkinson’s disease. Molecules.

[459] Lindqvist A (2013). Enhanced brain delivery of the opioid peptide DAMGO in glutathione pegylated liposomes: a microdialysis study. Mol. Pharm..

[460] Jia J (2009). Mechanisms of drug combinations: interaction and network perspectives. Nat. Rev. Drug Discov..

[461] Gao J (2022). Overcoming barriers for intra-articular delivery of disease-modifying osteoarthritis drugs. Trends Pharm. Sci..

[462] Preis E (2021). The chorioallantoic membrane as a bio-barrier model for the evaluation of nanoscale drug delivery systems for tumour therapy. Adv. Drug Deliv. Rev..

[463] Barui AK (2020). Cancer‐targeted nanomedicine: Overcoming the barrier of the protein corona. Adv. Ther..

[464] Wang Y (2021). Multistage adaptive nanoparticle overcomes biological barriers for effective chemotherapy. Small.

[465] Wang L-M, Wang Y-T, Yang W-X (2021). Engineered nanomaterials induce alterations in biological barriers: focus on paracellular permeability. Nanomedicine.

[466] Magar KT (2021). Liposome-based delivery of biological drugs. Chin. Chem. Lett..

[467] Jaeckle KA (2002). An open label trial of sustained-release cytarabine (DepoCyt™) for the intrathecal treatment of solid tumor neoplastic meningitis. J. Neuro Oncol..

[468] Glantz MJ (1999). A randomized controlled trial comparing intrathecal sustained-release cytarabine (DepoCyt) to intrathecal methotrexate in patients with neoplastic meningitis from solid tumors. Clin. Cancer Res..

[469] Guaglianone P (1994). Phase I and pharmacologie study of liposomal daunorubicin (DaunoXome). Invest N. Drug..

[470] Forssen E (1996). Fluorescence imaging studies for the disposition of daunorubicin liposomes (DaunoXome) within tumor tissue. Cancer Res..

[471] Judson I (2001). Randomised phase II trial of pegylated liposomal doxorubicin (DOXIL®/CAELYX®) versus doxorubicin in the treatment of advanced or metastatic soft tissue sarcoma: a study by the EORTC Soft Tissue and Bone Sarcoma Group. Eur. J. Cancer.

[472] Löwenberg B (2009). High-dose daunorubicin in older patients with acute myeloid leukemia. N. Engl. J. Med..

[473] Xiao Q (2018). A drug-delivering-drug strategy for combined treatment of metastatic breast cancer. Nanomed. Nanotechnol..

[474] Huang F (2022). A nanocrystal platform based on metal-phenolic network wrapping for drug solubilization. AAPS PharmSciTech..

[475] Xin X (2017). Rod‐shaped active drug particles enable efficient and safe gene delivery. Adv. Sci..

[476] Xin X (2018). Drug-delivering-drug platform-mediated potent protein therapeutics via a non-endo-lysosomal route. Theranostics.

[477] Mohammad IS (2019). Drug-delivering-drug approach-based codelivery of paclitaxel and disulfiram for treating multidrug-resistant cancer. Int J. Pharm..

[478] Aitipamula S (2012). Polymorphs, salts, and cocrystals: what’s in a name?. Cryst. Growth Des..

[479] Bolla G, Sarma B, Nangia AK (2022). Crystal engineering of pharmaceutical cocrystals in the discovery and development of improved drugs. Chem. Rev..

[480] Tang Q (2023). ROS-responsive prodrug micelle co-delivery system for synergistic antiatherosclerotic therapy. Mol. Pharm..

[481] Yin W (2022). Co-delivery systems of paclitaxel prodrug for targeted synergistic therapy of breast cancer. J. Drug Deliv. Sci. Tec..

[482] Dang Y, Guan J (2020). Nanoparticle-based drug delivery systems for cancer therapy. Smart Mater. Med..

[483] Gao C (2020). Treatment of atherosclerosis by macrophage-biomimetic nanoparticles via targeted pharmacotherapy and sequestration of proinflammatory cytokines. Nat. Commun..

[484] Singh AP, Biswas A, Shukla A, Maiti P (2019). Targeted therapy in chronic diseases using nanomaterial-based drug delivery vehicles. Signal Transduct. Target Ther..

[485] Chi KN (2018). Patient-reported outcomes following abiraterone acetate plus prednisone added to androgen deprivation therapy in patients with newly diagnosed metastatic castration-naive prostate cancer (LATITUDE): an international, randomised phase 3 trial. Lancet Oncol..

[486] Fizazi K (2019). Abiraterone acetate plus prednisone in patients with newly diagnosed high-risk metastatic castration-sensitive prostate cancer (LATITUDE): final overall survival analysis of a randomised, double-blind, phase 3 trial. Lancet Oncol..

[487] Wu Y (2011). Impact of lapatinib plus trastuzumab versus single-agent lapatinib on quality of life of patients with trastuzumab-refractory HER2+ metastatic breast cancer. Ann. Oncol..

[488] Blackwell KL (2012). Overall survival benefit with lapatinib in combination with trastuzumab for patients with human epidermal growth factor receptor 2-positive metastatic breast cancer: final results from the EGF104900 Study. J. Clin. Oncol..

[489] Mehta RS (2019). Overall Survival with Fulvestrant plus Anastrozole in Metastatic Breast Cancer. N. Engl. J. Med..

[490] Blumenschein GR (2012). Sunitinib plus erlotinib for the treatment of advanced/metastatic non-small-cell lung cancer: a lead-in study. J. Thorac. Oncol..

[491] Wang L, Lei X, Wang X (2022). Efficacy and Safety of PD-1/PD-L1 Inhibitor Chemotherapy Combined with Lung Cancer Fang No. 1 in Relapsed and Refractory SCLC: A Retrospective Observational Study. Comput Math. Methods Med..

[492] Wang J (2022). Efficacy of bevacizumab and gemcitabine in combination with cisplatin in the treatment of esophageal cancer and the effect on the incidence of adverse reactions. Biomed. Res Int..

[493] Montesinos P (2022). Ivosidenib and azacitidine in IDH1-mutated acute myeloid leukemia. N. Engl. J. Med..

[494] Schmid P (2018). Atezolizumab and nab-paclitaxel in advanced triple-negative breast cancer. N. Engl. J. Med..

[495] Batist G (2009). Safety, pharmacokinetics, and efficacy of CPX-1 liposome injection in patients with advanced solid tumors. Clin. Cancer Res..

[496] Lancet JE (2021). CPX-351 versus 7+3 cytarabine and daunorubicin chemotherapy in older adults with newly diagnosed high-risk or secondary acute myeloid leukaemia: 5-year results of a randomised, open-label, multicentre, phase 3 trial. Lancet Haematol..

[497] Swisher EM (2022). Impact of homologous recombination status and responses with veliparib combined with first-line chemotherapy in ovarian cancer in the Phase 3 VELIA/GOG-3005 study. Gynecol. Oncol..

[498] Wolchok JD (2017). Overall survival with combined nivolumab and ipilimumab in advanced melanoma. N. Engl. J. Med..

[499] Larkin J (2019). Five-year survival with combined nivolumab and ipilimumab in advanced melanoma. N. Engl. J. Med..

[500] Eikelboom JW (2017). Rivaroxaban with or without aspirin in stable cardiovascular disease. N. Engl. J. Med..

[501] Anand SS (2018). Rivaroxaban with or without aspirin in patients with stable peripheral or carotid artery disease: an international, randomised, double-blind, placebo-controlled trial. Lancet.

[502] Ballantyne CM (2018). Efficacy and safety of bempedoic acid added to ezetimibe in statin-intolerant patients with hypercholesterolemia: A randomized, placebo-controlled study. Atherosclerosis.

[503] Brunner G (2013). The Effect of Lipid Modification on Peripheral Artery Disease after Endovascular Intervention Trial (ELIMIT). Atherosclerosis.

[504] Ballantyne CM (2003). Effect of ezetimibe coadministered with atorvastatin in 628 patients with primary hypercholesterolemia: a prospective, randomized, double-blind trial. Circulation.

[505] Nicholls SJ (2011). Effects of the CETP inhibitor evacetrapib administered as monotherapy or in combination with statins on HDL and LDL cholesterol: a randomized controlled trial. JAMA.

[506] Bays HE (2010). Effects of prescription omega-3-acid ethyl esters on non–high-density lipoprotein cholesterol when coadministered with escalating doses of atorvastatin. Mayo Clin. Proc..

[507] Goldenberg NA, Krantz MJ, Hiatt WR (2012). L-Carnitine plus cilostazol versus cilostazol alone for the treatment of claudication in patients with peripheral artery disease: a multicenter, randomized, double-blind, placebo-controlled trial. Vasc. Med..

[508] Ballantyne CM (2020). Bempedoic acid plus ezetimibe fixed-dose combination in patients with hypercholesterolemia and high CVD risk treated with maximally tolerated statin therapy. Eur. J. Prev. Cardiol..

[509] Kereiakes DJ (2015). Efficacy and safety of the proprotein convertase subtilisin/kexin type 9 inhibitor alirocumab among high cardiovascular risk patients on maximally tolerated statin therapy: The ODYSSEY COMBO I study. Am. Heart J..

[510] Robinson JG (2015). Efficacy and safety of alirocumab in reducing lipids and cardiovascular events. N. Engl. J. Med..

[511] Simonneau G (2008). Addition of sildenafil to long-term intravenous epoprostenol therapy in patients with pulmonary arterial hypertension A randomized trial. Ann. Intern Med..

[512] Chin KM (2021). Three- versus two-drug therapy for patients with newly diagnosed pulmonary arterial hypertension. J. Am. Coll. Cardiol..

[513] McLaughlin V (2015). Bosentan added to sildenafil therapy in patients with pulmonary arterial hypertension. Eur. Respir. J..

[514] Pulido T (2013). Macitentan and morbidity and mortality in pulmonary arterial hypertension. N. Engl. J. Med..

[515] Sitbon O (2015). Selexipag for the treatment of pulmonary arterial hypertension. N. Engl. J. Med..

[516] White RJ (2019). Clinical outcomes stratified by baseline functional class after initial combination therapy for pulmonary arterial hypertension. Respir. Res..

[517] Tapson VF (2012). Oral treprostinil for the treatment of pulmonary arterial hypertension in patients on background endothelin receptor antagonist and/or phosphodiesterase type 5 inhibitor therapy (the FREEDOM-C study): a randomized controlled trial. Chest.

[518] Archer SL (2013). Riociguat for pulmonary hypertension–a glass half full. N. Engl. J. Med..

[519] Simonneau G (2014). Long-term sildenafil added to intravenous epoprostenol in patients with pulmonary arterial hypertension. J. Heart Lung Transplant..

[520] Genovese MC (2013). Efficacy and safety of secukinumab in patients with rheumatoid arthritis: a phase II, dose-finding, double-blind, randomised, placebo controlled study. Ann. Rheum. Dis..

[521] Genovese MC (2014). One-year efficacy and safety results of secukinumab in patients with rheumatoid arthritis: phase II, dose-finding, double-blind, randomized, placebo-controlled study. J. Rheumatol..

[522] Keystone EC (2004). Radiographic, clinical, and functional outcomes of treatment with adalimumab (a human anti-tumor necrosis factor monoclonal antibody) in patients with active rheumatoid arthritis receiving concomitant methotrexate therapy: a randomized, placebo-controlled, 52-week trial. Arthritis Rheum..

[523] Smolen JS (2014). Adjustment of therapy in rheumatoid arthritis on the basis of achievement of stable low disease activity with adalimumab plus methotrexate or methotrexate alone: the randomised controlled OPTIMA trial. Lancet.

[524] Strand V (2012). Health-related quality of life outcomes of adalimumab for patients with early rheumatoid arthritis: results from a randomized multicenter study. J. Rheumatol..

[525] Takeuchi T (2019). Efficacy and safety of peficitinib (ASP015K) in patients with rheumatoid arthritis and an inadequate response to methotrexate: results of a phase III randomised, double-blind, placebo-controlled trial (RAJ4) in Japan. Ann. Rheum. Dis..

[526] Taylor PC (2017). Baricitinib versus placebo or adalimumab in rheumatoid arthritis. N. Engl. J. Med..

[527] Emery P (2017). Certolizumab pegol in combination with dose-optimised methotrexate in DMARD-naïve patients with early, active rheumatoid arthritis with poor prognostic factors: 1-year results from C-EARLY, a randomised, double-blind, placebo-controlled phase III study. Ann. Rheum. Dis..

[528] Colombel JF (2010). Infliximab, azathioprine, or combination therapy for Crohn’s disease. N. Engl. J. Med..

[529] Rubin DT (2017). Budesonide multimatrix is efficacious for mesalamine-refractory, mild to moderate ulcerative colitis: A randomised, placebo-controlled trial. J. Crohns Colitis.

[530] Lanzolla G (2021). Statins for Graves’ orbitopathy (STAGO): a phase 2, open-label, adaptive, single centre, randomised clinical trial. Lancet Diabetes Endocrinol..

[531] Cheetham TD (2022). Adjuvant rituximab-exploratory trial in young people with graves disease. J. Clin. Endocrinol. Metab..

[532] Cole M (2019). Adjuvant rituximab, a potential treatment for the young patient with Graves’ hyperthyroidism (RiGD): study protocol for a single-arm, single-stage, phase II trial. BMJ open..

[533] Kahaly GJ (2018). Mycophenolate plus methylprednisolone versus methylprednisolone alone in active, moderate-to-severe Graves’ orbitopathy (MINGO): a randomised, observer-masked, multicentre trial. Lancet Diabetes Endocrinol.

[534] Bhatt DL (2020). Role of combination antiplatelet and anticoagulation therapy in diabetes mellitus and cardiovascular disease: insights from the COMPASS trial. Circulation.

[535] Matthews DR (2019). Glycaemic durability of an early combination therapy with vildagliptin and metformin versus sequential metformin monotherapy in newly diagnosed type 2 diabetes (VERIFY): a 5-year, multicentre, randomised, double-blind trial. Lancet.

[536] Mordi NA (2020). Renal and cardiovascular effects of SGLT2 inhibition in combination with loop diuretics in patients with type 2 diabetes and chronic heart failure: the RECEDE-CHF trial. Circulation.

[537] Atri A, Shaughnessy LW, Locascio JJ, Growdon JH (2008). Long-term course and effectiveness of combination therapy in Alzheimer disease. Alz Dis. Assoc. Dis..

[538] Sadowsky CH, Dengiz A, Meng X, Olin JT (2010). Switching from oral donepezil to rivastigmine transdermal patch in Alzheimer’s disease: 20-week extension phase results. Prim. Care Companion J. Clin. Psychiatry.

[539] Nirogi R (2022). Effect of concurrent use of memantine on the efficacy of masupirdine (SUVN-502): A post hoc analysis of a phase 2 randomized placebo-controlled study. Neurol. Ther..

[540] Standaert DG (2017). Effect of Levodopa-carbidopa Intestinal Gel on Non-motor Symptoms in Patients with Advanced Parkinson’s Disease. Mov. Disord. Clin. Pr..

[541] Hauser RA (2009). Double-blind trial of levodopa/carbidopa/entacapone versus levodopa/carbidopa in early Parkinson’s disease. Mov. Disord..

[542] Olanow CW (2021). Continuous subcutaneous levodopa delivery for Parkinson’s disease: A randomized study. J. Parkinsons Dis..

[543] Klivenyi P (2004). Additive neuroprotective effects of creatine and cyclooxygenase 2 inhibitors in a transgenic mouse model of amyotrophic lateral sclerosis. J. Neurochem..

[544] Ramirez P (2022). Pathogenic tau accelerates aging-associated activation of transposable elements in the mouse central nervous system. Prog. Neurobiol..

